# Remotely sensed forest understory density and nest predator occurrence interact to predict suitable breeding habitat and the occurrence of a resident boreal bird species

**DOI:** 10.1002/ece3.6062

**Published:** 2020-02-05

**Authors:** Julian Klein, Paul J. Haverkamp, Eva Lindberg, Michael Griesser, Sönke Eggers

**Affiliations:** ^1^ Department of Ecology Swedish University of Agricultural Sciences Uppsala Sweden; ^2^ Department of Evolutionary Ecology and Environmental Studies University of Zurich Zurich Switzerland; ^3^ Department of Forest Resource Management Swedish University of Agricultural Sciences Umeå Sweden; ^4^ Department of Anthropology University of Zurich Zurich Switzerland

**Keywords:** airborne laser scanning, forest thinning, habitat suitability models, LiDAR, nest predation, *Perisoreus infaustus*, understory

## Abstract

Habitat suitability models (HSM) based on remotely sensed data are useful tools in conservation work. However, they typically use species occurrence data rather than robust demographic variables, and their predictive power is rarely evaluated. These shortcomings can result in misleading guidance for conservation. Here, we develop and evaluate a HSM based on correlates of long‐term breeding success of an open nest building boreal forest bird, the Siberian jay. In our study site in northern Sweden, nest failure of this permanent resident species is driven mainly by visually hunting corvids that are associated with human settlements. Parents rely on understory nesting cover as protection against these predators. Accordingly, our HSM includes a light detection and ranging (LiDAR) based metric of understory density around the nest and the distance of the nest to the closest human settlement to predict breeding success. It reveals that a high understory density 15–80 m around nests is associated with increased breeding success in territories close to settlements (<1.5 km). Farther away from human settlements breeding success is highest at nest sites with a more open understory providing a favorable warmer microclimate. We validated this HSM by comparing the predicted breeding success with landscape‐wide census data on Siberian jay occurrence. The correlation between breeding success and occurrence was strong up to 40 km around the study site. However, the HSM appears to overestimate breeding success in regions with a milder climate and therefore higher corvid numbers. Our findings suggest that maintaining patches of small diameter trees may provide a cost‐effective way to restore the breeding habitat for Siberian jays up to 1.5 km from human settlements. This distance is expected to increase in the warmer, southern, and coastal range of the Siberian jay where the presence of other corvids is to a lesser extent restricted to settlements.

## Graphical abstract

A LiDAR informed habitat suitability model for breeding success based on biotic interactions and verified with large scale occurrence data, showed that corvids around human settlements suppress breeding success in a Taiga specialist, the Siberian jay. However, dense understory around the nest alleviated the nest predation pressure by corvids while an open understory led to highest breeding success, in the absence of corvids. This study suggests that redistributing forest thinning efforts according to the distance of the forest stand from human settlements may provide a cost‐neutral opportunity to restore high‐quality breeding habitat for Siberian jays.

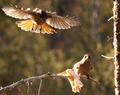

## 1 INTRODUCTION

Habitat suitability models (HSMs) relate environmental parameters to the likelihood of species occurrence. This makes them valuable tools for guiding management to minimize human impacts on biodiversity (Guisan et al., 2013). HSMs are, for example, used to assess the effect of land use changes on species richness (Rondinini et al., 2011; Vogeler et al., 2014) or to predict the distribution of suitable habitat at the landscape scale (Angelieri, Adams‐Hosking, Ferraz, de Souza, & McAlpine, 2016; Vierling, Swift, Hudak, Vogeler, & Vierling, 2014). To ensure that HSMs are reliable, we need a solid understanding of the factors that impact demography and thereby define suitable habitat (Krebs, 2002; Rushing, Ryder, & Marra, 2016). However, we often lack detailed large‐scale environmental and demographic data, which is required to assess how, for example, limiting resources (food supplies, nest sites, shelter) interact with disturbances (natural enemies) to determine suitable habitat and population numbers (incorporation of biotic interactions; Hirzel & Le Lay, 2008). In large ecosystems such as forests, land cover classification maps are often used to create HSMs (Bradley et al., 2012). However, these classifications are usually broad, and do not account for the horizontal and vertical distribution of forest vegetation, which are key drivers of biodiversity in forests (Franklin, 2002; MacArthur & MacArthur, 1961; McElhinny, Gibbons, Brack, & Bauhus, 2005). Hence, even if local field studies reveal a strong effect of the vertical distribution of forest vegetation for suitable breeding habitat, it can be difficult to scale this information up to the landscape level, hampering effective conservation actions (Garabedian, Moorman, Nils Peterson, & Kilgo, 2017). Here, Light Detection and Ranging (LiDAR) provides detailed 3D data on the forest vegetation and is therefore a useful tool to overcome this lack of large‐scale forest structural information (Davies & Asner, 2014). LiDAR has facilitated the identification and spatial quantification of ecological variables in HSMs for birds (Goetz et al., 2010; Hinsley, Hill, Gaveau, & Bellamy, 2002; Vogeler, Hudak, Vierling, & Vierling, 2013), mammals (Palminteri, Powell, Asner, & Peres, 2012), and invertebrates (Lindberg, Roberge, Johansson, & Hjältén, 2015; Vierling et al., 2011).

Habitat suitability models principally use occupancy data at a specific place and time. This approach may provide misleading indicators of habitat suitability if the mechanism that determines population numbers varies over time (Rushing et al., 2017), with the spatial scale (Chase & Leibold, 2002), or if habitat preferences reflect the integration of multiple environmental factors across multiple spatial scales (Chalfoun & Martin, 2007). Additionally, the presence or absence of individuals does not necessarily reflect suitable habitat in terms of demographic rates due to social interactions, predation, or the presence of nonbreeders (Horne, 1983; Johnson, 2007; Wheatley, Fisher, Larsen, Litke, & Boutin, 2005). Because of these uncertainties, LiDAR‐derived HSMs need to be validated against independently collected data at the relevant spatial scale to determine their applicability to conservation work. To our knowledge, only few studies have attempted to verify predictions of suitable habitat by comparing them with independently collected occurrence or demographic data (Unglaub, Steinfartz, Drechsler, & Schmidt, 2015; but see Anderson et al., 2016; Law et al., 2017).

In this study, we (a) test the usefulness of a LiDAR‐based metric of understory vegetation in assessing habitat suitability in terms of breeding success in the Siberian jay *Perisoreus infaustus*, (b) investigate if, and at which local scale understory density provides cover against the Siberian jay's main nest predators (other corvids), and if this depends on the distance from the nest to the closest human settlement, (c) extrapolate results from our local habitat model for breeding success to the broader regional scale, and (d) validate if our predictions of breeding success match independent occurrence data of Siberian jays as an indicator of habitat suitability. We used long‐term data of breeding success of Siberian jays and detailed knowledge of their breeding ecology, along with large‐scale population surveys to investigate these questions. Our goal is to empirically identify environmental parameters that reflect suitable breeding habitat for Siberian jays and provide recommendations for future forestry practices in boreal forests. Previous work has shown that forestry‐induced reductions in protective nesting cover interact with the high occurrence of corvid nest predators close to human settlements to reduce breeding success and group size (Eggers, Griesser, & Ekman, 2005; Griesser, Nystrand, Eggers, & Ekman, 2007; Muukkonen, Angervuori, Virtanen, Kuparinen, & Merila, 2012). Siberian jays rely on cryptic behavior and select nest sites within high understory density forests to reduce the risk of nest failure (Eggers, Griesser, & Ekman, 2008; Eggers, Griesser, Nystrand, & Ekman, 2006). This strategy, however, may incur higher thermoregulatory costs due to the colder microclimate in denser vegetation (Eggers et al., 2006). In absence of corvids, reproductive output is presumably increased in more open forests (Eggers, Griesser, Andersson, & Ekman, 2005). Hence, forest management that reconciles environmental and wood biomass production objectives is expected to be highly location and context dependent.

## 2 MATERIAL AND METHODS

### 2.1 Study system

We collected nesting data from 1998 to 2004 and 2011 to 2013 from an individually color‐ringed population of Siberian jays in northern Sweden, near Arvidsjaur (65°40′N, 19°10′E). This year‐round territorial, open nest building bird lives in family groups centered around a dominant breeding pair and occurs throughout the northern Palearctic (Ekman & Griesser, 2016). The reproductive season spans from the end of March to the end of May, where temperatures can fall below −20°C (SMHI, 2018).

The study site includes a managed (53 km^2^) and an unmanaged area (30 km^2^). The managed area is dominated by even‐aged forest patches with management cycles of about 100 years (Griesser & Lagerberg, 2012). A cycle involves clear‐cut harvesting, replanting, and repeated thinning, where the understory vegetation is often completely removed (Holm, 2015). The unmanaged area is part of a nature reserve and has been unaffected by forestry for at least 200 years. The density of human settlements in the study site is generally very low, but higher in the managed (1.34 persons/km^2^) than in the unmanaged (0.1/km^2^) area (SCB, 2014).

### 2.2 Breeding success data

Each March, females were radio‐tagged before egg laying (Holohil BD‐2G, Telenax TBX‐006; 1.8 g, corresponding to 2.0% of body mass; attachment of radio‐tags was done under the license of Umeå ethics board A80‐99, A45‐04, A50‐11). Once nests had been located during egg incubation, they were visited repeatedly to count the number of eggs, nestlings, and fledglings (for details of field methods see Griesser, Wagner, Drobniak, & Ekman, 2017). Locations of nests were recorded with a GPS. We categorized each breeding event (*n* = 251) as successful (i.e., at least one fledged offspring) or unsuccessful. Brood reduction or partial losses to predators are very rare (Eggers, Griesser, Andersson, et al., 2005). Renesting attempts in the same year are uncommon (*n* = 5) and were disregarded to assure comparability with all other nests with only one attempt. We excluded nests from breeders that were experimentally treated with brucellosis (*n* = 11) in 2012, leading to high nest‐failure rates (Griesser et al., 2017).

### 2.3 Corvid nest predation risk data

Detailed corvid distribution data are not available for the study site or larger regions within the Siberian jay's distribution range. We therefore developed a proxy for nest predation pressure by corvids for our HSM based on the habitat use of Eurasian jays *Garrullus glandarius* within our study site. Siberian jay territories with high reproductive success are typically located farther away from human settlements (i.e., one or more neighboring territories away; Ekman, Eggers, Griesser, & Tegelstrom, 2001). Evidence from natural nests indicates that the lower breeding success close to human settlements is caused mainly by Eurasian jays (Eggers, Griesser, Andersson, et al., 2005). Eurasian jays survive winter only by feeding on bird feeders located at most settlements in our study site. These feeders also attract other corvids (Eurasian magpie *Pica pica*, hooded crow *Corvus cornix*), particularly before snowmelt in early May, during the Siberian jay's reproductive season (Ekman et al., 2001). However, it remains unclear at which distance from human settlements Eurasian jays and other corvids influence breeding success of Siberian jays. Thus, we collected occurrence data of Eurasian jays near (≤50 m) Siberian jay nest (30 min long observation bouts), in spring 1998 and 2002 within 34 territories over 26 ± 2 hr (Eggers, Griesser, & Ekman, 2005). Then, we tested to what extent the observed change in Eurasian jay occurrence with distance to human settlements is linked to breeding success of Siberian jays. We modeled the effect of distance from nest sites to human settlements on breeding success using binned distances across 80% of the measured range (excluding the smallest and largest 10% of the distances to prevent effects of small sample sizes) of the nests from 500 to 3,500 m at intervals of 50 m and compared the models using AICc (Burnham & Anderson, 2002). The models' structure was the same as described under *statistical analysis* below. We expected the negative effect of corvid occurrence on breeding success to decrease sharply at a certain distance from human settlements, creating distinct “hot spots” of high nest predation risk close to settlements (see also Ekman et al., 2001). The locations of year‐round human settlements in the study site were determined from aerial photographs in QGIS (Lantmäteriet, 2016; QGIS Development Team, 2015).

### 2.4 Light detection and ranging data

Light detection and ranging (LiDAR) data are gathered by sensors mounted on airplanes that emit and receive laser pulses that sweep across the landscape below. The 3D coordinates of the point of reflection are informed by the measured distance and direction of the laser pulse combined with the position and orientation of the aircraft (Davies & Asner, 2014). At our study site, LiDAR data were collected on 29–30 September 2010, while LiDAR data for the landscape prediction of breeding success were collected between September 2009 and 2015 as part of a nationwide scanning scheme (Lantmäteriet, 2016). The point density was 0.5–1 point/m^2^ with 3 cm vertical and 25 cm horizontal precision. The elevation of the LiDAR data points was normalized based on a digital elevation model derived from the LiDAR data. To test the usefulness of LiDAR in explaining suitable breeding habitat, we extracted a forest understory density metric from the LiDAR data using Fusion software (McGaughey, 2015) with a 12.5 m raster pixel resolution (Swedish forestry maps standard). Data were extracted for forested areas only as Siberian jays do not use open areas (e.g., lakes, clear cuts, mires, roads, and power line corridors) as nesting sites. We chose 5 m, that is, half of the mean forest height at the study site (10 m) as the cutoff between the understory and the canopy. We defined density as the percentage of laser returns within a height interval, relative to all returns, and understory density as the percentage of all laser returns between 0.5 and 5 m. LiDAR data provide a measurement of forest vegetation for a specific point of time, but as vegetation growth is very slow in northern Sweden (Table A1), we regard the data from autumn 2010 representative for the whole study period (1998–2013). The LiDAR data were imported into R (R Development Core Team, 2017) and processed using the *raster* package (Hijmans et al., 2011). Throughout the study period, the spatial extent and type of forestry interventions (thinning, clear cutting) were recorded in the field. Using this information, we assigned a “no data” value to forest pixels that were clear‐cut before LiDAR acquisition, or where thinning occurred before or after acquisition. To test at what distance understory density influences breeding success, we calculated the understory density at the nest (nest location + GPS uncertainty = 15 m) and within 100 radii, cantered at the nest, representing 1%–100% of the average size of a Siberian jay territory (460 ha; Nystrand, Griesser, Eggers, & Ekman, 2010), resulting in 101 measurements of understory density for each nest. If more than 5% of the pixels within a certain radius around the nest had been assigned, a “no data” value of LiDAR data was not used for this nest at that particular radius (*n* = 10 excluded at the nest [15 m] to *n* = 44 at the territory border [460 m]). The percentage of the sampled area with forest cover was calculated for all radii.

### 2.5 Statistical analysis

All statistical models were built in R (R Development Core Team, 2017). We evaluated whether the effect of the understory density around the nest on Siberian jay breeding success depends on whether the nest was located close or far from human settlements, using GLMMs (binomial error structure, logit link function) with the *glmer* function from the *lme4* package (Bates & Maechler, 2010). We added the study site (managed, unmanaged) as a fixed effect because the presence of only two levels did not justify using this factor as a random effect (Bolker et al., 2009). The random effects were, year (1998–2013), and the ring number of both breeders. Model assumptions were verified in the *DHARMa* package using scaled residuals (Hartig, 2017), and the function *vif* in the package *car* (Fox et al., 2018) was used to test for multicollinearity among predictors. We used *MuMIn* (Barton, 2015) to calculate marginal (R^2^GLMM_(m)_) and conditional *R* ^2^ values (R^2^GLMM_(c)_) (Nakagawa & Schielzeth, 2013) for the evaluation of model fit. We expect that the same absolute change in understory density has a greater effect on breeding success at low understory densities compared to high ones, so we log‐transformed the LiDAR metric for understory density at the nest. Based on AICc values calculated with the *MuMIn* package (Barton, 2015), we checked whether this model with log‐transformed understory density was more parsimonious than models with quadratic and linear relationships between understory density and breeding success. We plotted the predictions using back‐transformed data and based on the model coefficients with the *ggplot2* package (Wickham, 2009). We tested whether the model coefficients were sensitivity to the breakpoint of the distance between the nest and human settlements.

Because we wanted to know within which radius around the nest understory density interacts with the distance of the nest to the closest human settlement to influence breeding success, we performed the analysis above with the understory densities within all radii from 15 to 460 m. However, results from neighboring radii could be similar merely due to the correlation of LiDAR data at neighboring radii and not due to the actual effect of understory density. In order to resolve this issue, we plotted the correlation coefficients of the understory densities at all radii with the understory density at the nest against the *R* ^2^ values of the respective models, using *ggplot2* (Wickham, 2009). In this analysis, we only used nests for which LiDAR data for all radii from the nest to the territory borders were available (*n* = 203). The analytical procedure was as above for the models at all radii, except that the models also contained the percentage forest cover within a certain radius as a fixed effect, as the same value for understory density could be based on different areal amount of forest at larger radii.

### 2.6 The landscape prediction

To extrapolate our results from the local habitat model to the broader regional scale, we selected municipalities with a similar climate (average yearly temperature ± 1°C; SMHI, 2019) that also lie away from the coast and the mountain range. This resulted in a region of 18,290 km^2^, including, from north to south, the municipalities Arvidsjaur, where our study site is located, Malå, Lycksele, and Åsele. The climate within this region becomes milder toward the south and toward the coast in the east (+2°C higher a.y.t. than the study site; SMHI, 2019). LiDAR data used for the landscape prediction were collected between September 2009 and 2015 (Lantmäteriet, 2016). Shapefiles with the location of human settlements within this region were taken from the year 2013 (SCB, 2014) to get the best temporal overlap with the Siberian jay census, human settlement census, and LiDAR data. We validated our HSM using a map with regional information on the probability of Siberian jay occurrence which was developed by Bradter et al. (2018; SBS distribution raster on p.1670). The map is based on data from the Swedish national bird survey during which censuses were performed on 2 km squares every 25 km throughout Sweden between 2000 and 2013 at approximately 5‐year intervals (Ottwall et al., 2009). Siberian jay breeding success is a good predictor of next year's group size (Eggers, Griesser, Andersson, et al., 2005). We therefore regard using data on the probability of occurrence as suitable for validating predicted probability of breeding success in the case of the Siberian jay. We calculated the predicted probability of breeding success based on our model of the interaction between understory density and the distance to the closest human settlement for every 12.5 m pixel with forest cover in this region. Here, LiDAR data within 15 m around every pixel center were used, but the same analysis was done with the largest radius around the nest that still explained reproductive success and compared. Because sites not chosen for nesting cannot result in a successful reproduction, we set the expected breeding success to zero for all pixels that were nonforest or outside the range of understory densities chosen for nesting (understory density range used for nesting close to human settlements: 0.6%–33.3%, and far from human settlements 3.1%–24.5%). To compare the predicted probability of breeding success raster (12.5 m pixels) to the probability of Siberian jay occurrence raster (2 km pixels), we aggregated the predicted values for breeding success for every 2 km pixel of the probability of occurrence raster. To validate model predictions of breeding success with independent national survey data on the probability of Siberian jay occurrence, we normalized (*z*_i_ = (*x*_i_ − min(*x*))/(max(*x*) − min(*x*)) the values of both rasters to the continuous 0–1 range and subtracted them from one another. We visualized the differences for the whole region in a map using QGIS 2.18 (QGIS Development Team, 2015) and in a density plot for all four municipalities.

## 3 RESULTS

### 3.1 Distance to human settlements and predation risk

**Figure A1:**
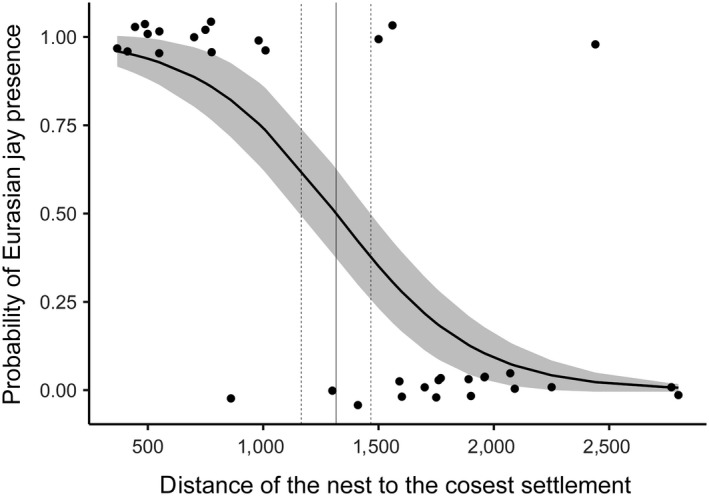
The probability of observing at least one Eurasian jay within 50 m from a Siberian jay nesting site during 26 ± 2 hr observation time. The probability is modeled as a response to the distance of the Siberian jay nest to the closest human settlement. The distance at which the predicted occurrence of Eurasian jays switches from 0 to 1 was 1,316 ± 151 m. The *R* ^2^‐value is .63

The occurrence of Eurasian jays near Siberian jay nests was closely associated with human settlements (mean ± *SE* = −0.003 ± 0.001, Pr(>|*z*|) = 0.001; Figure A1) and 63% of the variance in presence/absence was explained by this relationship. The distance at which the predicted occurrence of Eurasia jays switches from 0 to 1 was 1,316 ± 151 m. The comparisons of the effect of the binned distances of Siberian jay nests to human settlements on breeding success, indicated 1,450 m as the distance at which breeding success was substantially lower closer to human settlements and higher farther away (Table A2). The distribution of understory densities in the two distance categories was comparable which indicates no correlation between these potentially confounding factors (distance = far: mean ± *SD* = 13.1 ± 4.9, distance = close: mean ± *SD* = 12.6 ± 7.2).

### 3.2 Breeding success HSM

**Figure 1:**
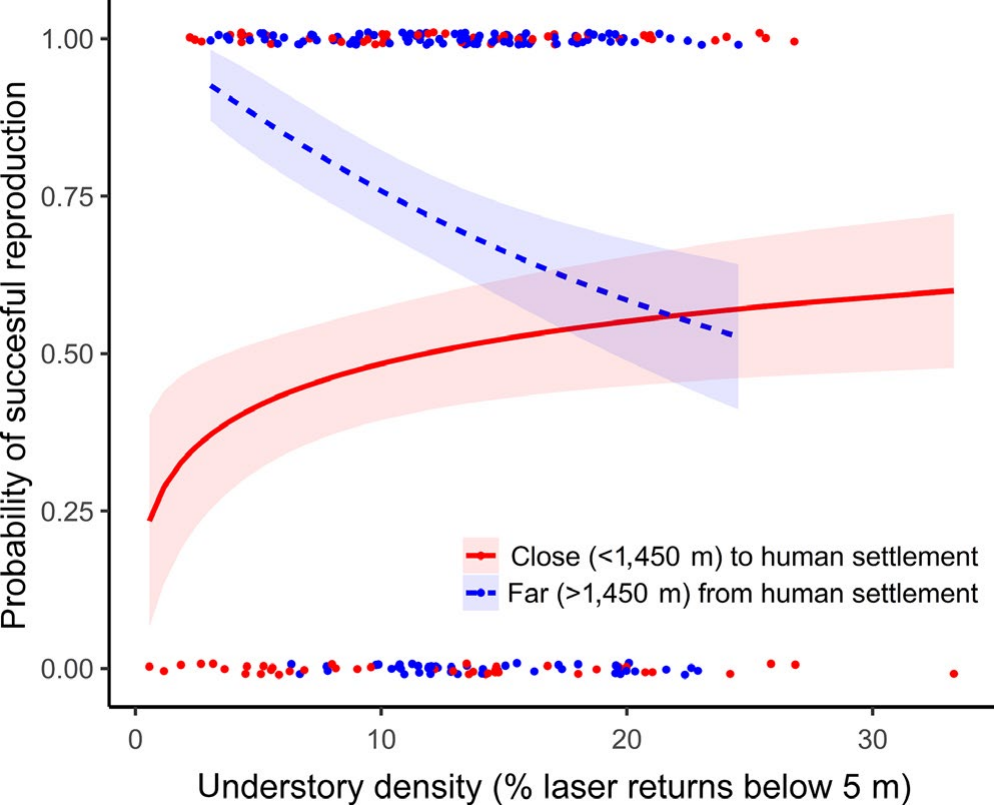
The effect of understory density at the nest on breeding success is shown for both high and low distance of the nest to the closest human settlement. The lines show predicted estimates while the ribbons show the standard error of the estimates. Data are back‐transformed for ease of viewing. Marginal and conditional *R* ^2^‐values = .10 resp. .18. Values of observed breeding success (0 = failure, 1 = success) of *n* = 235 nests are jittered to increase the visibility of individual data points

**Figure 2:**
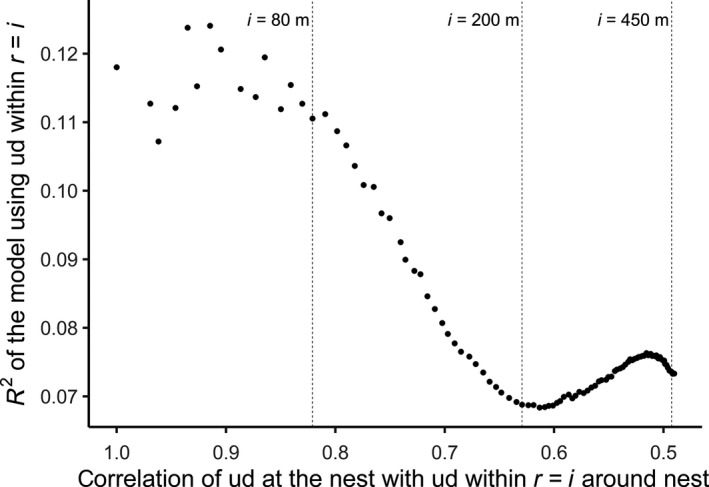
The marginal *R* ^2^‐values of the model for breeding success with average understory density (ud) at *i* = nest (15 m) to within radius *i* = territory border (460 m) around the nest plotted against the correlation of the understory density at radius *i*, with understory density at the nest (*n* = 203 nests). The distance of the nest to the closest human settlement in the models for breeding success is the same for all radii. Understory density within radii <80 m has an effect on Siberian jay breeding success

The model investigating the interaction between understory density and the distance to human settlements partially explained breeding success in the Siberian jay (*R* ^2^_(m)_ = .10, *R* ^2^_(c)_ = .18; Table 1). Very low understory density was associated with a decreased probability of breeding success only in areas close to human settlements. In contrast, low understory density was associated with successful reproduction in areas further away from settlements. In forests with very dense understory, breeding success was not affected by distance to human settlements (Figure 1). This pattern was consistent when using understory density values from 15 to 80 m around the nest (Figure 2). The explanatory power of models using understory density >80 m around the nest decreased in line with the decreasing correlation between understory densities at increasing radii with the understory density at 15 m (Figure 2). The most parsimonious model for breeding success included the log‐transformed understory density as a predictor (Table A3). The analysis which evaluated the sensitivity of this model's coefficients to the breakpoint between nests categorized as close or far from settlements yielded qualitatively (estimate + *SE* does not cross 0) the same results using distances between 1,350 m and 1,800 m (Table A4).

**Table 1:** Summary of the GLMM (binomial error structure, logit link function) with breeding success (0 = failure, 1 = success) as the response and an interaction between the distance of the nest to the closest human settlement and understory density at the nest together with the study area as covariates

Fixed effects	Estimate	*SE*	*z* Value	Pr(>|*z*|)
Intercept	0.24	0.30	0.81	0.417
Study area (unmanaged)	−0.52	0.36	−1.46	0.143
Distance to settlement (close)	**1.04**	**0.35**	**2.98**	**0.003**
Log(understory density)	0.40	0.31	1.30	0.194
Distance to settlement (close) × log(understory density)	**−1.57**	**0.60**	**−2.60**	**0.009**

Random effects	Variance
Male ID	0
Female ID	0
Year	0.33

Note

The year (1998–2013) and individual ID of both breeders were included as random effects. Significant (Pr(>|*z*|) < 0.05) effects are highlighted in bold (*n* = 235 nests).

### 3.3 Landscape prediction

**Figure 3:**
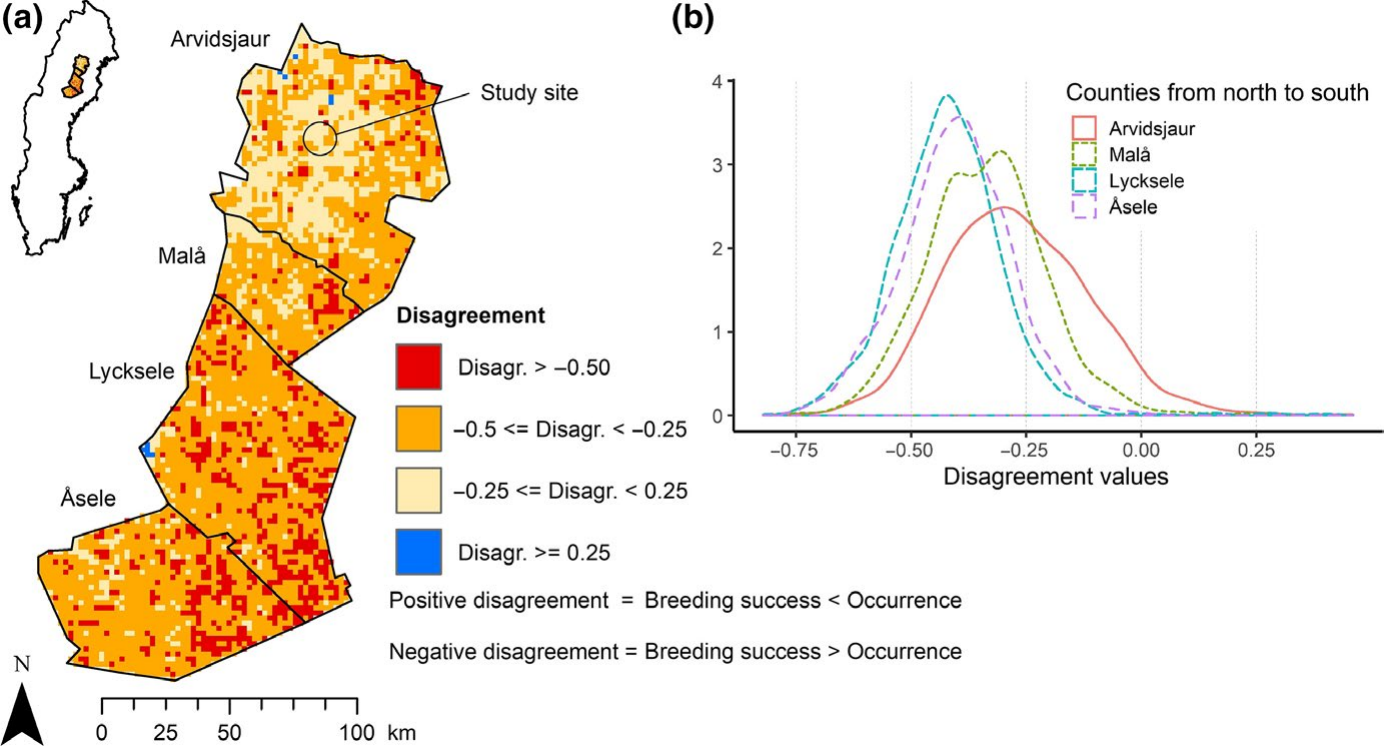
(a) The difference between the normalized predictions of Siberian jay occurrence and probability of successful reproduction in a large part of its Swedish distribution. Disagreement is, in almost all cases, based on an overestimation of breeding success. The disagreement calculation in this map is based on understory density data within 15 m around the center of every pixel and is highly correlated (pmcc = 0.84) with using data within 80 m. (b) The density of the disagreement values split by the four municipalities used for this landscape comparison

The landscape scale predictions of breeding success yielded values that were in line with the occurrence values from the independent national survey data in the region around the study site (Arvidsjaur municipality, within ca. 40 km radius; Figure 3). The agreement between predicted breeding success and Siberian jay occurrence data decreased in the southern part and closer to the coast in the east (Pearson's product‐moment correlation (ppmc) between the non‐normalized maps for the whole area = 0.38). Disagreement in most locations is due to relatively higher values for predicted breeding success in areas where Siberian jays are less commonly observed. The disagreement when using mean understory density data within 15 m was highly (ppmc = 0.84) correlated with the disagreement when using mean understory density data within 80 m.

## 4 DISCUSSION

### 4.1 Breeding success HSM & Landscape prediction

Our results show that the potential breeding success of Siberian jays is limited close to human settlements, particularly in forests with an open understory. This is most likely because of visually oriented corvid nest predators (primarily Eurasian jay), which we show occur more likely close to human settlements. The observed higher breeding success in open forests far away from human settlements is consistent with the idea that the amount of nesting cover reflects a trade‐off between predator protection gained from denser vegetation and thermoregulatory costs. Denser vegetation increases thermoregulatory costs due to a colder microclimate and reduces egg hatchability (Eggers et al., 2006; Marzluff, 1988; Wiebe & Martin, 1998), but decreases the likelihood of nest predation. Open forests, in contrast, are associated with a warmer microclimate. This leads to earlier snowmelt with an increased access to food in spring and reduced incubation costs, with a subsequently higher breeding success (Layton‐Matthews, Ozgul, & Griesser, 2018). Our results suggest that forest understory density within ca. 15–80 m around the nest affects breeding success. Most previous studies have only assessed the effect of proximal nest concealment on breeding success (Forstmeier & Weiss, 2004; Wiebe & Martin, 1998). We suggest that larger scale habitat structure (patch‐level) is relevant for species where visually hunting predators follow parents to locate nests, as is the case with Siberian jays (Eggers et al., 2008). Assessing nest‐site selection at larger scales may therefore improve our understanding of how exposure to nest predators and habitat structure interact to influence breeding success. If only proximal nest concealment is believed to be relevant, managers may underestimate the required spatial extent of a habitat factor crucial for conservation. Clearly, the spatial requirements of nest cover are species and context specific, and hence, the critical scale can be expected to vary across species (Bellamy, Scott, & Altringham, 2013; Manzer & Hannon, 2005).

LiDAR has become a standard tool in building HSMs for forest dwelling organisms (Davies & Asner, 2014). While LiDAR metrics associated with suitable habitat vary with the focal species, many avian studies have found the vertical distribution of the canopy biomass to be critical for breeding success (Goetz et al., 2010), adult survival (Eggers & Low, 2014), occupancy (Vogeler et al., 2013), and species richness (Lindberg et al., 2015). Similar to our study, Rechsteiner, Zellweger, Gerber, Breiner, and Bollmann (2017) reported LiDAR metrics related to early successional forest structure (understory) as the crucial factor for the conservation of the hazel grouse (*Tetrastes bonasia*), with high understory density associated with higher occurrence. For great tits (*Parus major*), vegetation density has been found to shape breeding microclimate, explaining nesting body mass variation (Hinsley, Hill, Bellamy, & Balzter, 2006). Most HSMs lack both a detailed mechanistic understanding as well as demographic data and are therefore often based on a list of potentially important noninteracting explanatory variables and species occurrence data (Hirzel & Le Lay, 2008). In contrast, we use a previously established biotic interaction between nest concealment and a proxy for nest predator occurrence to build a LiDAR‐based HSM for demographic rates. To our knowledge, no previous study has attempted to use a similar approach. Moreover, HSMs are rarely evaluated for their spatial and temporal predictive power against independently collected information, probably as a consequence of lacking detailed data (but see Anderson et al., 2016; Law et al., 2017; Rittenhouse, Thompson, Dijak, Millspaugh, & Clawson, 2010; Unglaub et al., 2015). This evaluation is crucial, because HSMs can only be used for management recommendations if they can be applied on a larger scale (Graf, Mathys, & Bollmann, 2009; Rechsteiner et al., 2017). While Rittenhouse et al. (2010) and Unglaub et al. (2015) evaluated occupancy‐based HSMs based on predictability of independently collected demographic rates, our study uses the opposite approach: We show how independently collected occupancy data can evaluate demographic rates based on a predicted HSM.

Our HSM showed good agreement between predicted breeding success and occupancy in the region (40 km) around the study site, but the breeding success was overestimated compared to occurrence, particularly in the southern and the eastern part of the area included in the HSM. The climate is milder further south and east of the study site. A higher temperature is associated with a generally higher occurrence of the more temperate species, including hooded crows, Eurasian jays, and Eurasian magpies (Artdatabanken, 2019; Valkama, Vepsäläinen, & Lehikoinen, 2011), and possibly also with a higher occurrence of these species further away from human settlements. Presumably, this has negative consequences for Siberian jay breeding success. A better understanding of the distribution of corvids in the whole region might therefore improve our model. A common limitation of developing HSMs for large regional scales is constraining factors (i.e., keeping variables constant) that are not integrated into the model (Guisan & Thuiller, 2005), as was necessary for climate in our model. In our model, the breeding year was indeed the factor explaining most variance. Breeding conditions vary drastically among years, and thus, the annual breeding success varies between 10% and 94% (Griesser, Halvarsson, Sahlman, & Ekman, 2014). Still, we see a high agreement between the compared maps in the region around the study site. We might make this observation because Siberian jay population size, which is the base of the probability of occurrence data, is across many years shaped by the underlying mechanism for breeding success which we describe in this study. Some of the discrepancy between predicted breeding success and Siberian jay occupancy might also be due to variables not always being correlated in reality. While breeding success is a good predictor of census population size (Eggers, Griesser, Andersson, et al., 2005), data on the probability of occurrence can be based on the observation of a single individual. In Siberian jays, breeders can remain in their territory for many years, even if reproduction consistently fails (Griesser et al., 2007), which can bias the correlation between probability of occurrence and breeding success (Griesser & Lagerberg, 2012).

### 4.2 Conservation implications

Our results provide clear and relevant suggestions for forest management in boreal forests where forestry practices are similar to the practices at the study site. When thinning occurs close to human settlements (<1.5 km), Siberian jays require the retention of dense understory patches with a radius of 15–80 m radius to increase breeding success. In contrast, thinning of very dense patches of the same size far away from human settlements can lead to higher breeding success. Thus, redistributing forest thinning from being random in relation to human settlements toward the above recommendations throughout the landscape should increase overall breeding success. Likewise, as corvids are more abundant in the warmer and more populated southernmost and coastal parts of the Siberian jay's Swedish distribution range (Artdatabanken, 2019), moderate thinning intensities (e.g., “Understory Retention Thinning” URT; Eggers & Low, 2014) are suggested in this region at larger distances to human settlements than in our study region. Informed planning of the location and intensity of forest thinning considering locations of settlements and local climate is therefore the key to useful conservation work. Our HSM, if applied to regions with a similar climate, can be a useful planning tool for the Siberian jay and other threatened taiga specialist birds. Extrapolating to regions with a different climate could be possible with detailed knowledge on the spatial and temporal distribution of corvid nest predators in relation to human settlements. The importance of understory rich forests is furthermore likely to increase in the future, considering that the corvids in this system are expected to expand their range toward the north and inland with a warmer climate (Thomas, 2010).

### 4.3 Conclusion

A LiDAR metric for understory density, in interaction with a proxy for nest predator occurrence, explained breeding success in Siberian jays and provides insight into the radius around the nest within which understory vegetation affects reproduction. The good agreement between predicted breeding success and independently collected occurrence data ca. 40 km around the study site demonstrates a low bias of our HSM's in areas with similar climatic conditions. The LiDAR‐based HSM for demographic rates presented here goes beyond the widespread use of occurrence data for HSMs. It is based on the interaction between two biotic factors previously known to influence Siberian jay demography. Our results have clear management implications and show how redistributing forest thinning in the landscape from being random to being strategic in relation to the distance from human settlements could increase overall breeding success in Siberian jays. We suggest that this can be achieved by reducing thinning close to human settlements on patches with a radius of ca. 15–80 m and increasing the thinning intensity in very dense stands far away from human settlements on patches of ca. 15–80 m. Extrapolation of this HSM to regions with different climatic conditions is possible with more detailed knowledge on the spatial and temporal distribution of corvid nest predators in relation to human settlements.

## CONFLICT OF INTEREST

We the authors of this study declare that no competing interests exist.

## AUTHORS' CONTRIBUTIONS

SE and MG collected the data. JK, PH, and EL analyzed the data, and JK led the writing of the manuscript. All authors contributed to ideas, discussions, and revision of the manuscript.

## Data Availability

LiDAR and human settlement data can be downloaded at https://maps.slu.se/. The Siberian jay occurrence map can be required from its authors (https://doi.org/10.1111/2041-210X.13012). Other data used in the analysis is on dryad (https://doi.org/10.5061/dryad.p2ngf1vmq). The code for the analysis and its executions are provided in the Appendix S1.

## Appendix 1

### LiDAR data acquisition specifications

The exact specification varies slightly for different areas of the landscape prediction map as different scanning units were used. We here give the specification for the study site. The deviation relative to the landscape prediction map is expected to be marginal. The scanning unit used in the study site was a Leica ALS50‐II system with a scanning angle of ±20°. The flight altitude varied between 1,700 and 2,300 m.a.s., but was constant for each flight line which had an overlap of 20%. The resulting laser footprint had a diameter of 0.4–0.8 m. The raw data consist of a point cloud with each point classified as ground, water, bridge, or unclassified points. Classification was done by means of TerraScan 010.020 V8i (Original classification: 2011‐03‐14; Latest classification: 2016‐04‐23) (Lantmäteriet, 2016. Product description: Laser data [LiDAR], 2.1, pp.1–11).

**Table A1:** Annual increase in volume on productive stands (>1 m^3^ ha^‐1^ yr^‐1^) in the study site, supporting that ALS data from autumn 2010 were representative for the whole study period (Swedish NFI, 2015)

Age class	0–20	21–40	41–60	61–80	81–100	101–120	121+	Total
Change in m^3^/ha	0.5	2.6	3.6	3.1	2.8	1.9	1.6	2.4

**Table A2:** Corvid nest predators are observed mostly near human settlements during the breeding season of Siberian jays. Thus, their negative effect on Siberian jay breeding success is expected to decrease abruptly at a certain distance from year‐round inhabited human settlements. This creates clearly distinct areas of high and low nest predation risk. In the table below, the results of the categorisation of the distance of the Siberian jay nest to the closest settlement into *close* (high nest predation risk) and *far* (low nest predation risk) are shown. We selected this threshold distance according to the lowest AIC value. In order to not have an unbalanced data set, the threshold analysis included 80% of all nests, excluding the 10% closest and 10% furthest away

Categorisation distance (m)	Estimate	*SE*	*z* value	Pr(>|z|)	$R_{\text{marginal}}^{2}]]>$	AICc
1,450	0.95	0.33	2.93	0.003	0.047	314.82
1,700	0.95	0.33	2.85	0.004	0.046	315.16
1,650	0.90	0.34	2.66	0.008	0.041	315.94
1,500	0.88	0.32	2.74	0.006	0.041	315.97
2,250	1.05	0.41	2.59	0.010	0.042	316.06
1,800	0.93	0.36	2.63	0.009	0.042	316.07
1,750	0.92	0.35	2.62	0.009	0.042	316.09
1,400	0.86	0.32	2.65	0.008	0.038	316.48
2,300	0.99	0.41	2.43	0.015	0.036	317.11
1,850	0.87	0.36	2.43	0.015	0.035	317.29
1,550	0.80	0.32	2.48	0.013	0.034	317.39
2,350	0.97	0.41	2.35	0.019	0.034	317.57
1,350	0.79	0.32	2.45	0.014	0.032	317.59
1,900	0.81	0.36	2.27	0.023	0.031	318.11
1,950	0.83	0.37	2.26	0.024	0.031	318.15
2,450	0.96	0.44	2.21	0.027	0.030	318.27
2,200	0.83	0.38	2.20	0.027	0.029	318.43
1,600	0.73	0.33	2.22	0.026	0.028	318.49
2,500	0.94	0.44	2.12	0.034	0.028	318.71
2,400	0.88	0.42	2.11	0.035	0.027	318.81
2,100	0.75	0.36	2.09	0.036	0.025	319.05
2,150	0.75	0.36	2.08	0.038	0.025	319.07
1,250	0.67	0.32	2.10	0.035	0.024	319.23
1,300	0.67	0.32	2.10	0.035	0.024	319.23
800	0.81	0.41	1.99	0.047	0.021	319.63
750	0.82	0.41	1.98	0.048	0.020	319.71
2,050	0.68	0.36	1.88	0.060	0.020	319.98
2,000	0.62	0.36	1.72	0.085	0.017	320.62
1,200	0.56	0.33	1.69	0.090	0.015	320.81
2,550	0.69	0.42	1.63	0.104	0.015	320.87
550	0.76	0.47	1.61	0.108	0.013	321.10
650	0.67	0.43	1.54	0.124	0.012	321.32
700	0.67	0.43	1.54	0.124	0.012	321.32
600	0.66	0.45	1.47	0.141	0.011	321.53
2,900	0.70	0.50	1.40	0.163	0.012	321.56
850	0.56	0.39	1.43	0.151	0.011	321.6
2,750	0.62	0.46	1.37	0.172	0.011	321.68
1,000	0.47	0.35	1.37	0.172	0.010	321.80
900	0.49	0.37	1.34	0.182	0.009	321.88
1,150	0.44	0.33	1.34	0.179	0.010	321.89
2,850	0.60	0.47	1.26	0.206	0.009	321.98
500	0.68	0.53	1.27	0.203	0.008	322.05
2,800	0.56	0.46	1.22	0.223	0.009	322.09
950	0.43	0.36	1.19	0.232	0.008	322.23
2,600	0.50	0.43	1.16	0.244	0.008	322.25
1,050	0.37	0.34	1.10	0.273	0.006	322.46
1,100	0.35	0.34	1.05	0.294	0.006	322.56
2,950	0.48	0.50	0.95	0.340	0.005	322.69
2,700	0.41	0.45	0.91	0.362	0.005	322.78
3,300	0.42	0.51	0.83	0.404	0.004	322.92
3,000	0.33	0.50	0.67	0.504	0.003	323.17
3,050	0.33	0.50	0.67	0.504	0.003	323.17
3,100	0.33	0.50	0.67	0.504	0.003	323.17
3,150	0.33	0.50	0.67	0.504	0.003	323.17
3,200	0.33	0.50	0.67	0.504	0.003	323.17
2,650	0.28	0.45	0.63	0.531	0.002	323.23
3,250	0.28	0.50	0.55	0.581	0.002	323.32
3,350	0.17	0.52	0.33	0.739	0.000	323.51
3,400	0.17	0.52	0.33	0.739	0.000	323.51
3,450	0.17	0.52	0.33	0.739	0.000	323.51
3,500	0.11	0.52	0.21	0.834	0.000	323.58

**Table A3:** We log‐transformed understory density since we expected that the same absolute change in understory density has a greater effect on breeding success at low understory densities compared to high densities. The results below verify that the model with log‐transformed understory density was more parsimonious than models with quadratic and linear relationships between understory density and breeding success. The table shows the evaluation results for the models with LiDAR data at the nest. Parsimony was measured with Akaike’s information criterion (*n* = 235 nests)

Explanatory model	AICc	Delta
Distance to settlement * log(understory density) + study site	302.0	0
Distance to settlement * understory density + study site	304.4	2.36
Distance to settlement * understory density^2^ + study site	305.1	3.08
Distance to settlement + study site	305.3	3.22
Intercept	309.0	6.96
Study site	311.1	9.04
Understory density + study site	312.7	10.64

**Table A4:** We performed a sensitivity analysis of the main results to the categorisation of the distance of the Siberian jay nests to the closest human settlement, to verify that our results are not overly sensitive to the calculated threshold distance. The results of the interaction term between understory density and distance to settlement (close/far) is shown below. The results are sorted according to the AICc value

Categorisation distance (m)	Estimate	*SE*	*z* value	Pr(>|z|)	$R_{\text{marginal}}^{2}]]>$	AICc
1,450	−1.57	0.60	−2.60	0.009	0.10	301.4
1,500	−1.65	0.61	−2.71	0.007	0.09	301.8
1,550	−1.53	0.61	−2.51	0.012	0.08	304.5
1,600	−1.62	0.61	−2.64	0.008	0.08	304.7
1,400	−1.22	0.56	−2.18	0.029	0.07	305.4
1,650	−1.22	0.61	−1.99	0.046	0.07	305.7
1,700	−0.93	0.62	−1.51	0.132	0.06	306.8
1,800	−0.74	0.64	−1.16	0.246	0.05	308.7
1,350	−0.81	0.53	−1.54	0.124	0.04	309.1
1,750	−0.61	0.63	−0.97	0.333	0.05	309.1
1,850	−0.62	0.66	−0.94	0.346	0.04	310.4
2,250	−0.56	0.81	−0.69	0.491	0.04	310.4
2,300	−0.62	0.82	−0.76	0.449	0.04	310.6
1,900	−0.65	0.65	−1.00	0.317	0.03	311.1
2,450	−0.64	0.86	−0.74	0.458	0.03	311.1
2,500	−0.64	0.86	−0.74	0.458	0.03	311.1
2,350	−0.54	0.84	−0.63	0.526	0.03	311.1
1,250	−0.68	0.51	−1.33	0.183	0.03	311.3
1,300	−0.68	0.51	−1.33	0.183	0.03	311.3
1,950	−0.59	0.66	−0.90	0.370	0.03	311.3
800	−0.20	0.53	−0.38	0.704	0.03	311.9
750	−0.08	0.53	−0.16	0.873	0.02	312.1
2,400	−0.67	0.86	−0.78	0.436	0.03	312.1
2,200	−0.19	0.76	−0.25	0.801	0.02	313.1
2,550	−0.71	0.86	−0.83	0.409	0.02	313.2
550	−0.31	0.56	−0.56	0.575	0.02	313.2
1,200	−0.55	0.50	−1.10	0.270	0.02	313.3
2,100	−0.19	0.72	−0.26	0.792	0.02	313.6
650	−0.20	0.54	−0.38	0.705	0.02	313.7
700	−0.20	0.54	−0.38	0.705	0.02	313.7
2,150	0.00	0.73	0.01	0.995	0.02	313.7
600	−0.22	0.54	−0.41	0.683	0.02	313.9
2,750	−0.74	0.94	−0.78	0.433	0.02	314.1
2,900	−0.75	1.02	−0.73	0.463	0.02	314.1
2,000	−0.36	0.68	−0.53	0.596	0.01	314.2
850	−0.05	0.51	−0.09	0.927	0.01	314.2
2,600	−0.85	0.88	−0.97	0.334	0.01	314.3
2,050	−0.34	0.69	−0.48	0.629	0.01	314.3
2,800	−0.88	0.97	−0.91	0.364	0.02	314.3
2,850	−0.73	0.99	−0.74	0.461	0.01	314.5
900	0.13	0.51	0.26	0.797	0.01	314.5
1,000	0.11	0.50	0.23	0.818	0.01	314.5
500	−0.07	0.57	−0.12	0.903	0.01	314.5
1,150	−0.30	0.49	−0.61	0.539	0.01	314.6
950	0.26	0.51	0.51	0.610	0.01	314.7
2,650	−0.96	0.90	−1.07	0.283	0.01	315.1
2,950	−0.84	1.03	−0.82	0.413	0.01	315.1
3,300	−0.99	1.07	−0.92	0.358	0.01	315.2
2,700	−0.72	0.90	−0.79	0.428	0.01	315.2
3,250	−0.97	1.05	−0.92	0.356	0.01	315.6
1,100	−0.07	0.49	−0.15	0.884	0.01	315.6
1,050	−0.05	0.49	−0.10	0.923	0.01	315.6
3,000	−0.81	1.02	−0.80	0.425	0.01	315.7
3,050	−0.81	1.02	−0.80	0.425	0.01	315.7
3,100	−0.81	1.02	−0.80	0.425	0.01	315.7
3,150	−0.81	1.02	−0.80	0.425	0.01	315.7
3,200	−0.81	1.02	−0.80	0.425	0.01	315.7
3,350	−0.34	1.17	−0.29	0.768	0.00	316.6
3,400	−0.34	1.17	−0.29	0.768	0.00	316.6
3,450	−0.34	1.17	−0.29	0.768	0.00	316.6
3,500	−0.18	1.18	−0.15	0.881	0.00	316.7
