# Supplementary material for: Remotely sensed forest understory density and nest predator occurrence interact to predict suitable breeding habitat and the occurrence of a resident boreal bird species
Source: Ecol Evol. 2020 Feb 5;10(4):2238–52. doi: 10.1002/ece3.6062 (PMC7042737; doi:10.1002/ece3.6062)
Supplement: Supplementary file 1 [file ECE3-10-2238-s001.pdf]

Remotely sensed forest understory density and nest predator occurrence interact to predict suitable breeding habitat and the occurrence of a resident boreal bird species - Part 1

Julian Klein

14 May 2019

Part 1: LiDAR data and distance to settlement calculation

The LiDAR data is based on rasters extracted from .las files with FUSION. Raw Lidar data can be downloaded at http://maps.slu.se or www.lantmateriet.se. The download is not free unless you have free institutional access.

The shape files showing the study area and the forestry interventions as well as the positions of the nest and the year round human settlement in the study area will be archived on https://datadryad.org/. Specifications in the manuscript.

Some terms in this analysis:

ALS = LiDAR data, vd = vegetation density, dts = distance to closest human setllement

Start:

1. Load all packages:

```
library(data.table)
library(raster)
library(dplyr)
```

2. Define all functions: This function extracts the mean of all data from ALS_by_year within i metres.

```
extract_around_nest <- function(x) {

  ## We want to extract all data in ALS_by_year within i metres around each nest
  all <- extract(ALS_by_year[[paste0("y_", x$year)]], x[, c("X", "Y")], buffer = i)
  all <- as.data.frame(all)

  ## dts is extracted only at the nest since it is the same for all
  dts <- extract(ALS_by_year[[paste0("y_", x$year)]]$dts, x[, c("X", "Y")])

  ## We need the ratio of no data within i metres around the nest
  nd_ratio <- sum(na.omit(all[, 1] == -9999))/sum(!is.na(all[, 1]))
```

```
  if(nd_ratio < nd_thresh) {
    ## If less than nd_thresh the mean for all ALS metrics is calculated
    all[all == -9999] <-  NA
    out <- c(colMeans(all[, - length(all)], na.rm = TRUE), "dts" = dts)
  } else {
    ## If than nd_thresh NA is returned, except for dts
    all[] <- NA
    out <- c(colMeans(all[, - length(all)]), "dts" = dts)
  }

  ## The data for nest x is returned
  return(cbind(x, t(out)))

}
```

3. Load all data:

```
## Text files
nest_pos <- read.csv("data/nest_positions.csv")
settl <- read.csv("data/settlement_positions.csv")

## Raster files
ALS <- merge(stack(c("data/unmanaged_ElevP95.asc",
                     "data/unmanaged_5_Perc_above.asc",
                     "data/unmanaged_0.5_Perc_above.asc")),
             stack(c("data/managed_ElevP95.asc",
                     "data/managed_5_Perc_above.asc",
                     "data/managed_0.5_Perc_above.asc")))
names(ALS) <- c("height", "vd_5to", "vd_0to")

## Shape files
study_area <- shapefile("data/120ha_buffer_study.shp")
forestry <- shapefile("data/forestry.shp")

head(nest_pos)
```

```
##   territory year          name        X       Y
## 1 akkaliden 2002 akkaliden2002 691713.7 7288923
## 2 akkatjarn 2000 akkatjarn2000 691294.5 7288788
## 3 akkatjarn 2002 akkatjarn2002 691355.4 7288798
## 4 akkatjarn 2003 akkatjarn2003 691295.8 7288768
## 5 akkatjarn 2004 akkatjarn2004 691633.6 7288936
## 6 akkatjarn 2012 akkatjarn2012 691537.1 7288900
```

```
plot(ALS)
```

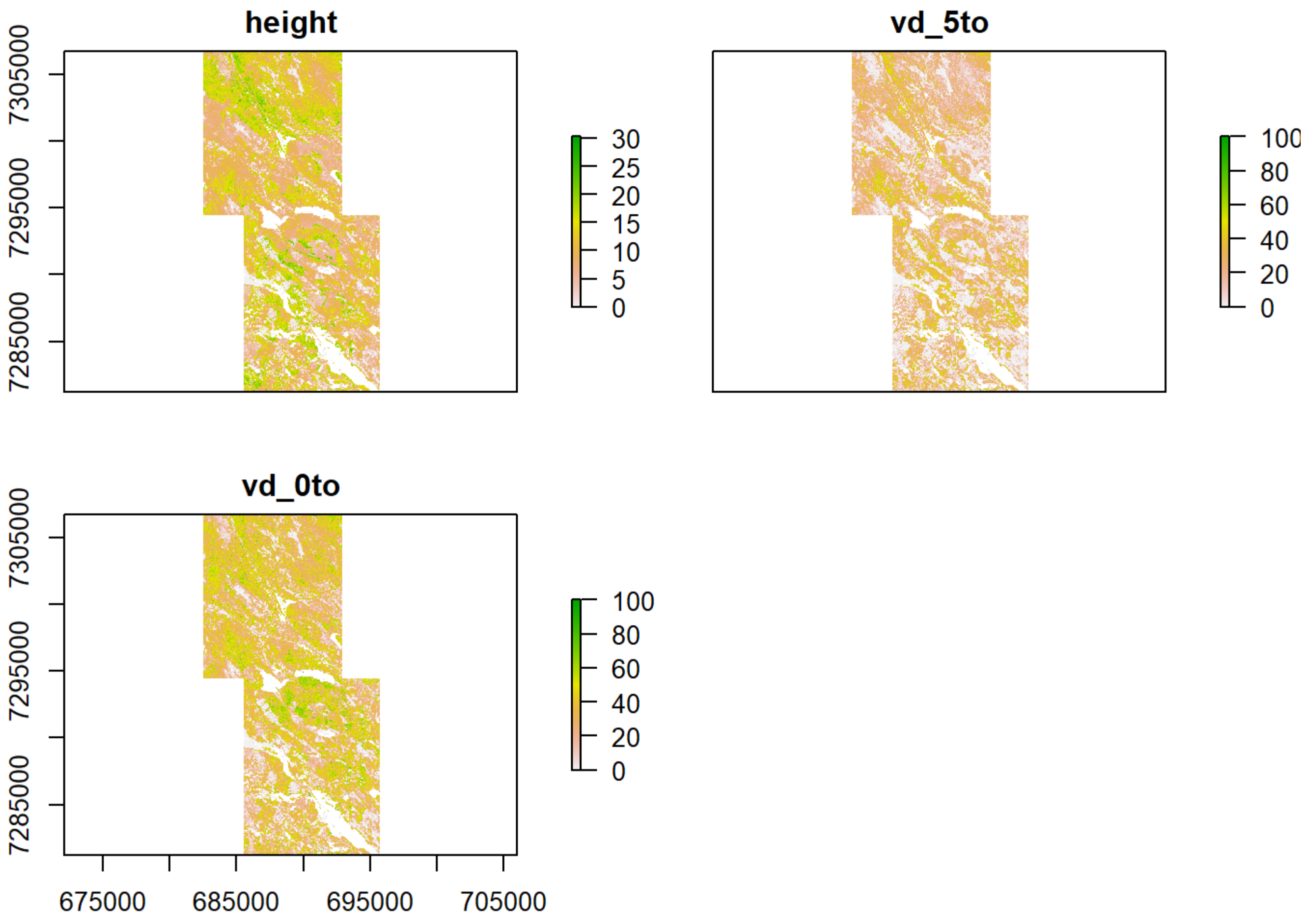


```
plot(study_area)
```

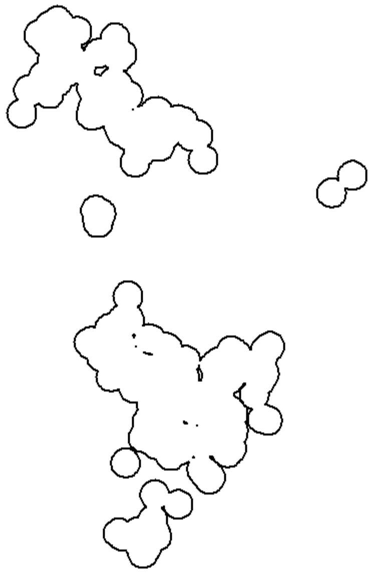

```
plot(forestry)
```

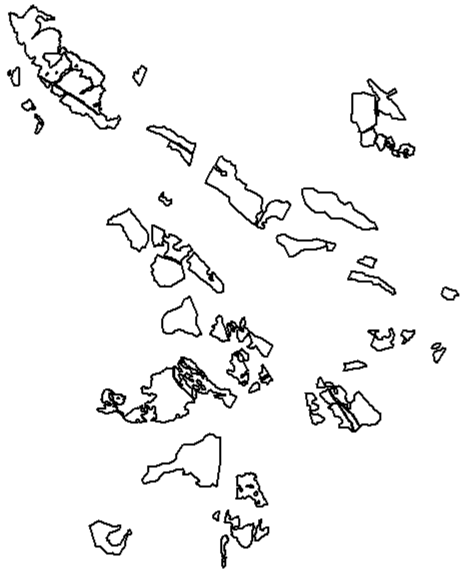

4. Process ALS data and adjust it to forestry interventions:

```
## Calculate absolute percentage returns between 0.5 and 5m
ALS$vd_0to5 <- ALS$vd_0to - ALS$vd_5to

## Reduce ALS data to needed layers
ALS <- ALS[[c(1, 4)]]

## Reduce ALS data set to study area
ALS <- mask(ALS, study_area)

## Add forest area layer: All pixels with forest height below 2 m or NA(water)
ALS$area <- !(ALS[["height"]][] < 2 | is.na(ALS[["height"]][]))

## Create a ALS data sample from all clear cuts in the study area. We use this later on in the
 script
sample_cc <- mask(ALS, forestry[forestry@data$GRIDCODE %in% as.character(1997:2010) & forestry
@data$action == "cut", ])

## Duplicate ALS data from collection year in 2010 onwards for all years we have nest data for

## Create list for yearly ALS data for which nest data exists and store ALS data in it
ALS_by_year <- vector("list", length(c(1998:2004, 2011:2013)))
ALS_by_year[1:length(ALS_by_year)] <- ALS ## Ignore warning!
names(ALS_by_year) <- paste0("y_", c(1998:2004, 2011:2013))
```

```
## In the following loop we replace pixels that were cut after collection year 2010 with rando
m samples from clear cuts from 1997 to 2009.

T1 <- NULL

for(i in 2011:2013) {

  ## Store ALS data used in this loop in T1 to make understanding easier
  T1 <- ALS_by_year[[paste0("y_", i)]]

  ## Which forestry shapes were cut between collection year 2010 and year i
  B1 <- forestry@data$GRIDCODE %in% as.character(2010:i) & forestry@data$action == "cut"

  ## Replace all pixels not NA which were cut after collection year 2010 with
  ## random samples from clear cuts from 1997 to 2009.
  if(sum(B1) > 0) {
    B2 <- !is.na(mask(T1, forestry[B1, ])[[1]][])
    T1[B2] <- sampleRandom(sample_cc, sum(B2))
  }

  ## Update forest are layer with new height data on modelled clear cuts
  T1$area <- !(T1[["height"]][] < 2 | is.na(T1[["height"]][]))

  ## Store T1 in respective place in ALS_by_year list
  ALS_by_year[[paste0("y_", i)]] <- T1

  T1 <- NULL

}
```

5. Add no data (-9999) to ALS layers. Replace pixels with -9999 where no data exists. These pixels are forests older than a clear cut or thinnings that occured before data acquisition in 2010 as well as forests after thinnings that happened after 2010. No data pixels bear the value -9999 because NA is used for water.

```
## layer names needed for after loop
names <- names(ALS_by_year[[1]])

for(i in unique(nest_pos$year)) {

  if(i < 2010) {
    ## All cc and thinnings after year i until 2010 are no data
    ## i+1 because it is the interventions in the years after i that matter
    B3 <- forestry@data$GRIDCODE %in% as.character((i+1):2010)
  } else {
    ## All thinnings after 2010 become no data
    B3 <- forestry@data$GRIDCODE %in% as.character(2010:i) & forestry@data$action == "thinned"
  }

  if(sum(B3) > 0) {
    for(j in 1:length(ALS[1])) {
      ## All forestry shapes which are TRUE in B3 need to become no data
      ALS_by_year[[paste0("y_", i)]][[j]] <- rasterize(forestry[B3, ], ALS_by_year[[paste0("y_
", i)]][[j]], update = TRUE, field = "value")
```

```
    }
  }

  names(ALS_by_year[[paste0("y_", i)]]) <- names

}
```

6. Create and add settlement layer to the ALS_by_year. In one location a settlement was abandoned in 2004. Therefore there are different layers until Spring 2004 and after.

```
## Until 2004, because one settlement disappeard after 2004
dts_u04 <- distanceFromPoints(ALS[[1]], as.matrix(settl[, 1:2]))
names(dts_u04) <- "dts"

dts_a04 <- distanceFromPoints(ALS[[1]], as.matrix(settl[settl$Settlement != "Fika_until_2004",
 1:2]))
names(dts_a04) <- "dts"

## Add to ALS_by_year
ALS_by_year[paste0("y_", 1998:2004)] <- lapply(ALS_by_year[paste0("y_", 1998:2004)], FUN = fun
ction(x) stack(x, dts_u04))
ALS_by_year[paste0("y_", 2011:2013)] <- lapply(ALS_by_year[paste0("y_", 2011:2013)], FUN = fun
ction(x) stack(x, dts_a04))
```

7. Extract ALS data from ALS_by_year for different radiuses around the nest and export.

```
nest_pos <- as.data.table(nest_pos)

## Calculate radiuses for fractions of territory size 6.6E5m2
rad <- c(15, ceiling(sqrt(6.6E5/pi)*seq(0.04, 1, 0.01)))

## Define no data threshhold accepted (now 5% no data)
nd_thresh <- 0.05

## Extract data for all radiuses around the nest

ALS_out <- NULL
T2 <- NULL

for(i in rad) {

  T2 <- nest_pos[, extract_around_nest(.SD), by = 1:nrow(nest_pos)]
  T2$sample_rad <- i

  ALS_out <- rbind(ALS_out, T2)

}

head(ALS_out); str(ALS_out)
```

```
##    nrow territory year          name        X       Y   height   vd_0to5
## 1:    1 akkaliden 2002 akkaliden2002 691713.7 7288923 15.59658  6.830820
```

```
## 2:    2 akkatjarn 2000 akkatjarn2000 691294.5 7288788 16.32880 11.581750
## 3:    3 akkatjarn 2002 akkatjarn2002 691355.4 7288798 17.56212  8.217848
## 4:    4 akkatjarn 2003 akkatjarn2003 691295.8 7288768 14.19360 12.302175
## 5:    5 akkatjarn 2004 akkatjarn2004 691633.6 7288936 16.05760 13.422980
## 6:    6 akkatjarn 2012 akkatjarn2012 691537.1 7288900       NA        NA
##    area      dts sample_rad
## 1:    1 1919.864         15
## 2:    1 1934.433         15
## 3:    1 1982.094         15
## 4:    1 1919.599         15
## 5:    1 1981.411         15
## 6:   NA 2028.321         15
```

```
## Classes 'data.table' and 'data.frame':   26166 obs. of  11 variables:
##  $ nrow      : int  1 2 3 4 5 6 7 8 9 10 ...
##  $ territory : Factor w/ 72 levels "akkaliden","akkatjarn",..: 1 2 2 2 2 2 2 3 3 3 ...
##  $ year      : int  2002 2000 2002 2003 2004 2012 2013 2000 2001 2013 ...
##  $ name      : Factor w/ 267 levels "akkaliden2002",..: 1 2 3 4 5 6 7 8 9 10 ...
##  $ X         : num  691714 691295 691355 691296 691634 ...
##  $ Y         : num  7288923 7288788 7288798 7288768 7288936 ...
##  $ height    : num  15.6 16.3 17.6 14.2 16.1 ...
##  $ vd_0to5   : num  6.83 11.58 8.22 12.3 13.42 ...
##  $ area      : num  1 1 1 1 1 NA NA 1 1 1 ...
##  $ dts       : num  1920 1934 1982 1920 1981 ...
##  $ sample_rad: num  15 15 15 15 15 15 15 15 15 15 ...
##  - attr(*, ".internal.selfref")=<externalptr>
```

The data set “ALS_rep_succ.csv” is merged with nest data and used in Part 3. The territory names seen in the data presented here are encoded in the data which is published.

Remotely sensed forest understory density and nest predator occurrence interact to predict suitable breeding habitat and the occurrence of a resident boreal bird species - Part 2

Julian Klein

26 November 2019

Part 2: The analysis pf Eurasian jay activity near Siberian jay nests in relation to the distance from this nest to the closest human settlement.

Start:

1. Load all packages:

```
library(lme4)
library(MuMIn)
library(DHARMa)
library(ltm)
library(ggplot2)
library(MASS)
```

2. Define all functions:

```
## Test model assumptions
test_my_model <- function(m.out) {

  sim <- simulateResiduals(m.out)
  plot(sim)
  print(testUniformity(sim))
  print(testZeroInflation(sim))
  print(testDispersion(sim))

}
```

3. Load all data:

```
## Eurasian jay data:
pred <- read.csv("data/corvid_data.csv"); head(pred)
```

```
##      territory cj_presence  dts
## 1        Kikki           1  367
## 2      Stabmok           1  550
## 3  Dyrasbäcken           0 1770
## 4     Hjelmers           0 1300
## 5 Guortestjärn           0 1760
## 6      Nyliden           0 1700
```

4. Build a GLM with a logit link.

```
m <- glm(cj_presence ~ dts, data = pred, family = binomial)

summary(m); r.squaredGLMM(m)
```

```
## 
## Call:
## glm(formula = cj_presence ~ dts, family = binomial, data = pred)
## 
## Deviance Residuals: 
##     Min       1Q   Median       3Q      Max  
## -1.8563  -0.6031  -0.1211   0.4634   2.7501  
## 
## Coefficients:
##              Estimate Std. Error z value Pr(>|z|)   
## (Intercept)  4.402842   1.473935   2.987  0.00282 **
## dts         -0.003345   0.001020  -3.280  0.00104 **
## ---
## Signif. codes:  0 '***' 0.001 '**' 0.01 '*' 0.05 '.' 0.1 ' ' 1
## 
## (Dispersion parameter for binomial family taken to be 1)
## 
##     Null deviance: 47.016  on 33  degrees of freedom
## Residual deviance: 25.573  on 32  degrees of freedom
## AIC: 29.573
## 
## Number of Fisher Scoring iterations: 5
```

```
##                   R2m       R2c
## theoretical 0.6315899 0.6315899
## delta       0.5842223 0.5842223
```

```
## Test model assumptions with DHARMa.
test_my_model(m)
```

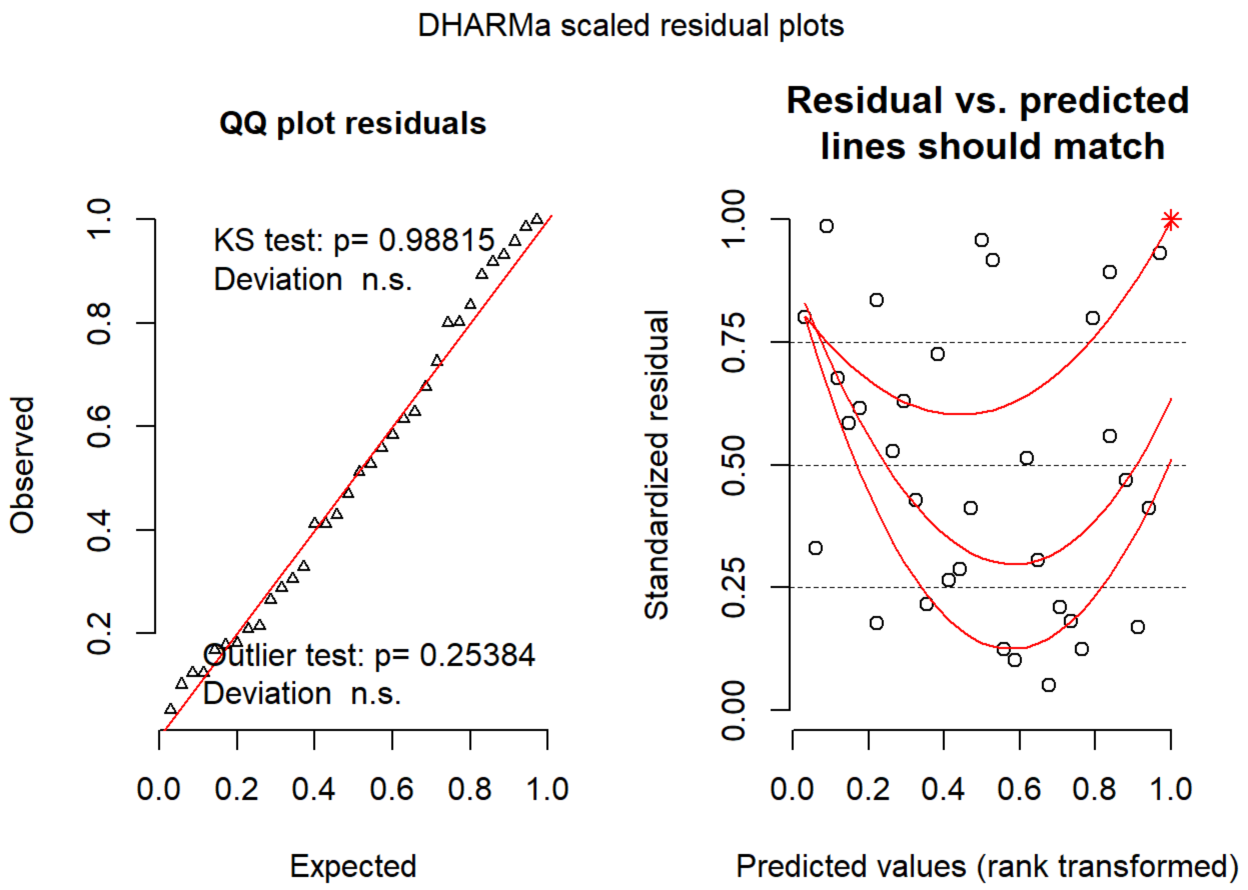
DHARMa scaled residual plots
QQ plot residuals
KS test: p= 0.98815
Deviation n.s.
Outlier test: p= 0.25384
Deviation n.s.
Observed
Expected
0.0
0.2
0.4
0.6
0.8
1.0
Residual vs. predicted
lines should match
Standardized residual
Predicted values (rank transformed)
0.00
0.25
0.50
0.75
1.00

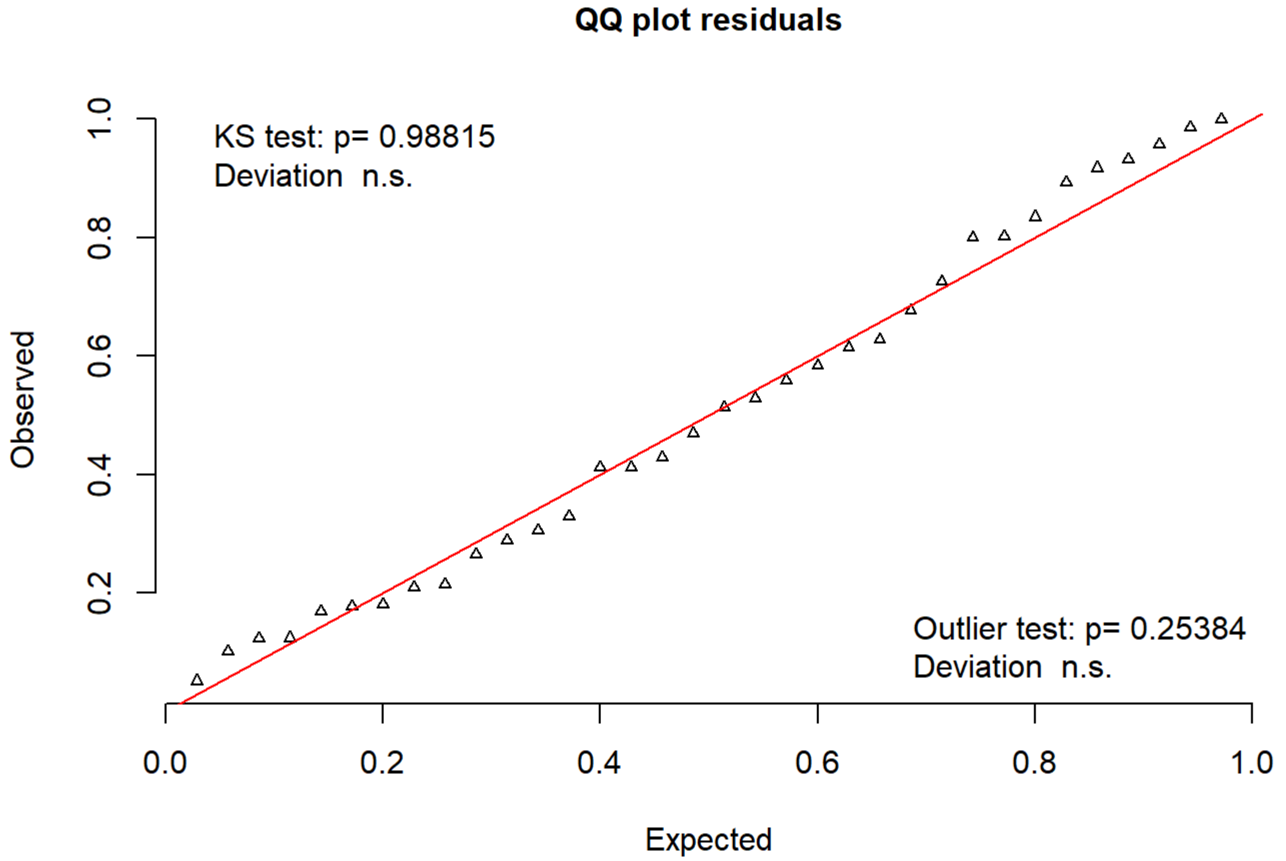
QQ plot residuals
KS test: p= 0.98815
Deviation n.s.
Outlier test: p= 0.25384
Deviation n.s.
Observed
Expected
0.0
0.2
0.4
0.6
0.8
1.0

```
##
##  One-sample Kolmogorov-Smirnov test
##
## data:  simulationOutput$scaledResiduals
## D = 0.07258, p-value = 0.9881
## alternative hypothesis: two-sided
```

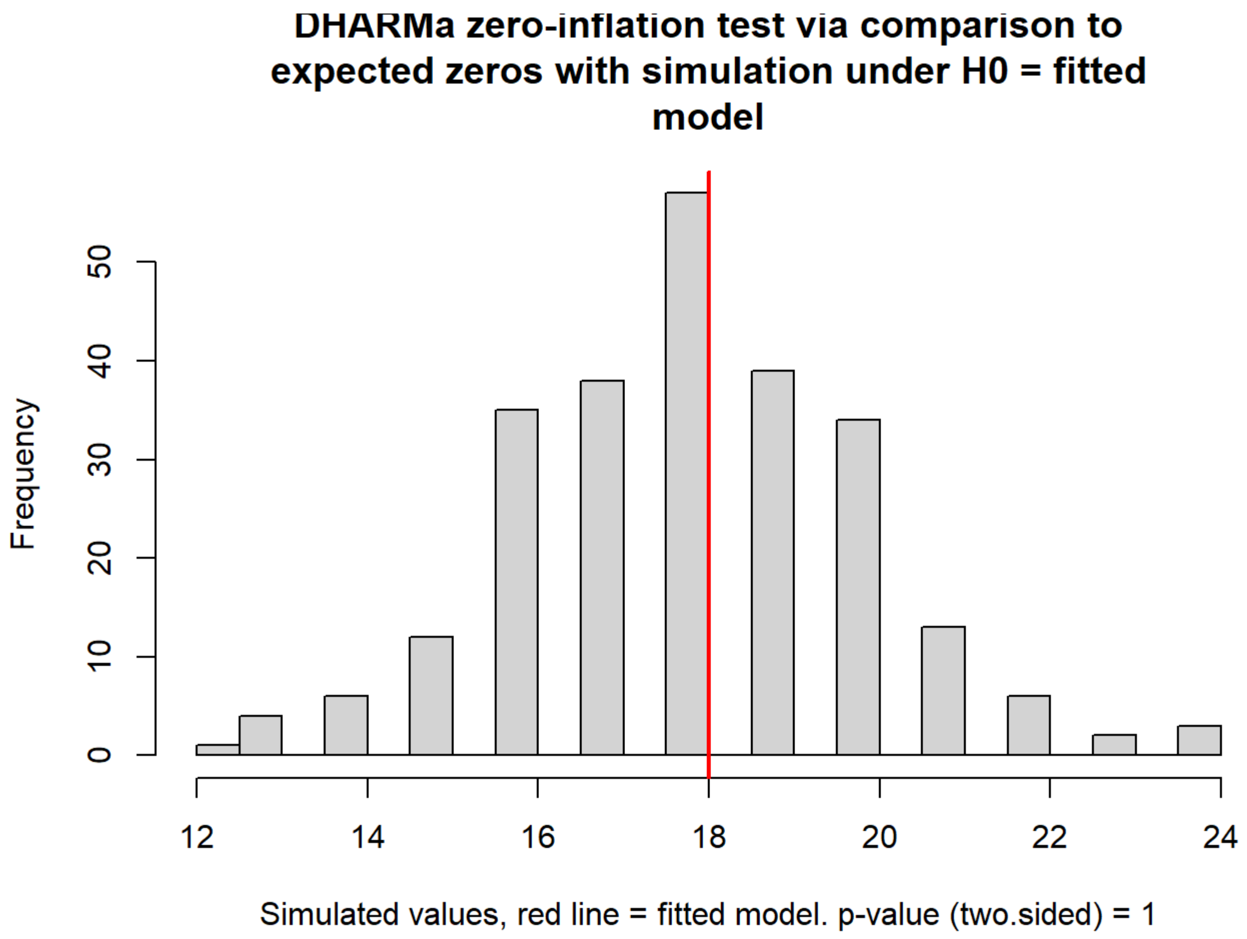


```
##
##  DHARMa zero-inflation test via comparison to expected zeros with
##  simulation under H0 = fitted model
##
## data:  simulationOutput
## ratioObsSim = 0.99911, p-value = 1
## alternative hypothesis: two.sided
```

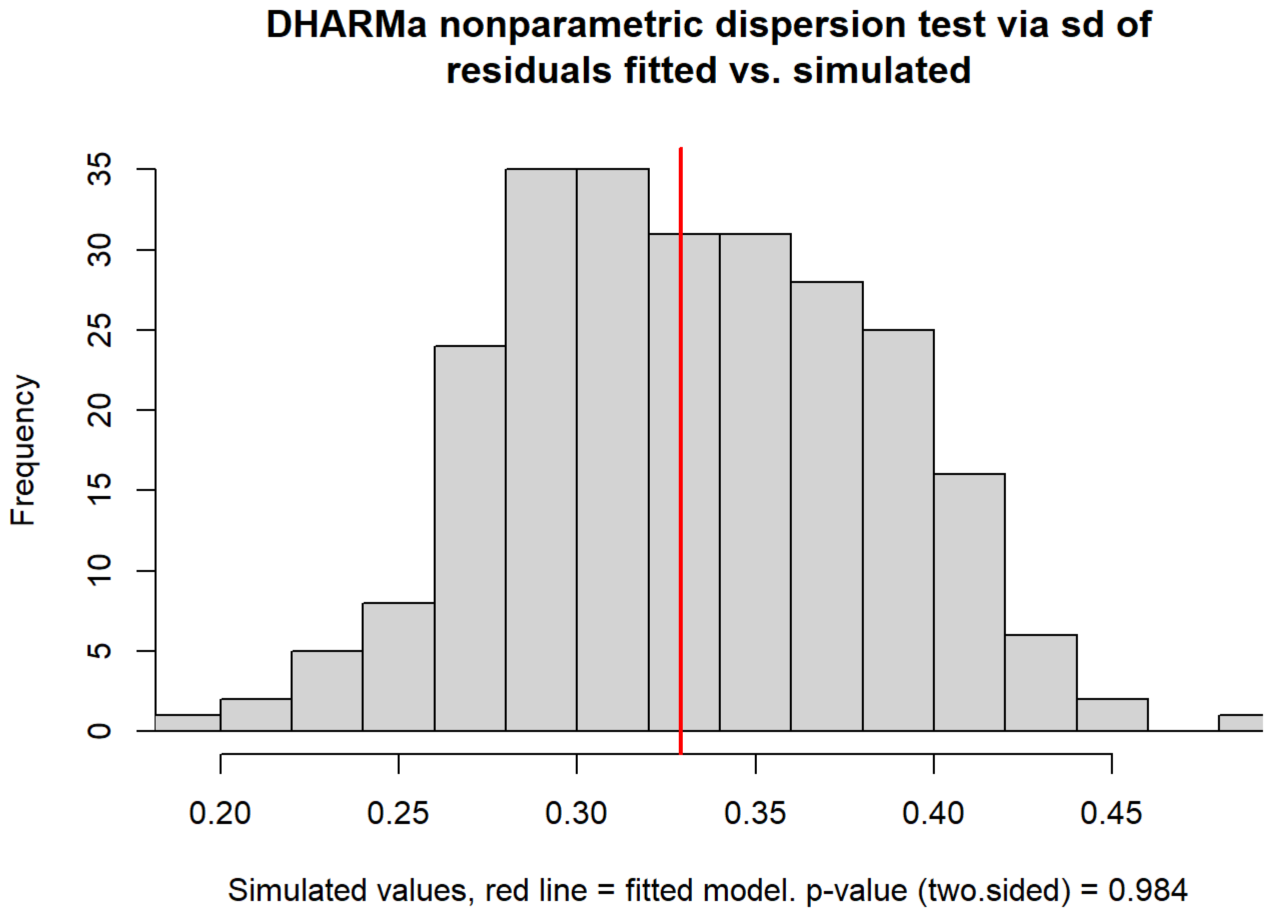


```
##
##  DHARMa nonparametric dispersion test via sd of residuals fitted
##  vs. simulated
##
## data:  simulationOutput
## ratioObsSim = 0.99229, p-value = 0.984
## alternative hypothesis: two.sided
```

5. Make predictions, calculate x for (p=0.5) and make a figure.

```
## Predict response:
pred_pred <- predict(m, re.form = NA, se.fit = TRUE, type = "response")

## Calculate LD50:
dose.p(m, p = 0.5)
```

```
##              Dose       SE
## p = 0.5: 1316.309 151.1881
```

```
## Make a plot:
ggplot() +
geom_line(aes(x = pred$dts, y = pred_pred$fit), size = 2) +
geom_ribbon(aes(x = pred$dts, ymin = pred_pred$fit - pred_pred$se.fit, ymax = pred_pred$fit +
pred_pred$se.fit), alpha = 0.2) +
```

```
geom_jitter(aes(x = pred$dts, y = pred$cj_presence), size = 2, height = 0.01) +
scale_x_continuous(breaks = c(500, 1000, 1500, 2000, 2500)) +
geom_vline(xintercept = 1316) + geom_vline(xintercept = c(1165, 1467), linetype = "dashed") +
xlab("distance of the nest to the cosest settlement") +
ylab("probability of Eurasian jay presence") +
theme_classic(15)
```

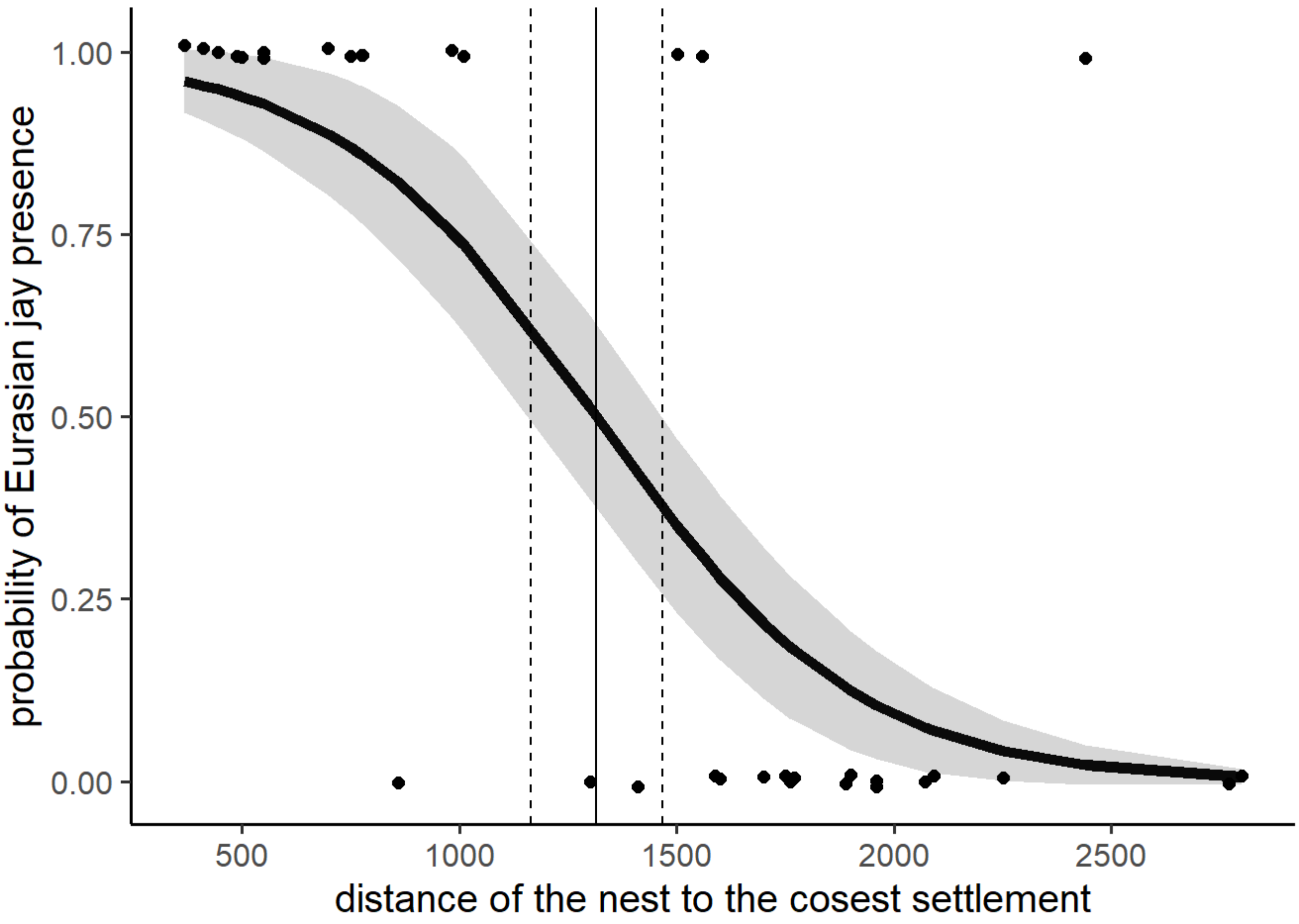

Remotely sensed forest understory density and nest predator occurrence interact to predict suitable breeding habitat and the occurrence of a resident boreal bird species - Part 3

Julian Klein

19 November 2019

Part 3: The whole analysis of breeding success is perfomred in this script. The landscape prediction comparison is in part 4.

Start:

1. Load all packages:

```
library(data.table)
library(MuMIn)
library(DHARMa)
library(lme4)
library(car)
library(ggplot2)
library(reshape)
library(MASS)
```

2. Define all functions:

```
## Specify control values for all models in this script
cont_spec <- glmerControl(optimizer = "bobyqa", optCtrl = list(maxfun = 100000))

## Test model assumptions
test_my_model <- function(m.out) {

  sim <- simulateResiduals(m.out)
  plot(sim)
  print(testUniformity(sim))
  print(testZeroInflation(sim))
  print(testDispersion(sim))
  print("Variance Inflation Factor:")
  print(vif(m.out))
```

```
}
```

3. Load all data:

```
## ALS data and nest data comnbined
nest_ALS <- read.csv("data/siberian_jay_data.csv")

head(nest_ALS); str(nest_ALS)
```

```
##    name year hab_qual female_ring male_ring rep_succ    vd_0to5      area
## 1 y2002 2002  managed        9313     57079        1 12.272976 0.9926829
## 2 y2002 2002  managed        9313     57079        1  7.861546 1.0000000
## 3 y2002 2002  managed        9313     57079        1 13.670386 0.9559038
## 4 y2002 2002  managed        9313     57079        1 12.502614 0.9801980
## 5 y2002 2002  managed        9313     57079        1 12.766219 0.9499662
## 6 y2002 2002  managed        9313     57079        1 12.820096 0.9457041
##        dts sample_rad
## 1 1919.864        202
## 2 1919.864         37
## 3 1919.864        376
## 4 1919.864        225
## 5 1919.864        276
## 6 1919.864        294
```

```
## 'data.frame':    24402 obs. of  10 variables:
##  $ name       : Factor w/ 249 levels "a1998","a1999",..: 231 231 231 231 231 231 231 231 23
1 231 ...
##  $ year       : int  2002 2002 2002 2002 2002 2002 2002 2002 2002 2002 ...
##  $ hab_qual   : Factor w/ 2 levels "managed","unmanaged": 1 1 1 1 1 1 1 1 1 1 ...
##  $ female_ring: int  9313 9313 9313 9313 9313 9313 9313 9313 9313 9313 ...
##  $ male_ring  : int  57079 57079 57079 57079 57079 57079 57079 57079 57079 57079 ...
##  $ rep_succ   : int  1 1 1 1 1 1 1 1 1 1 ...
##  $ vd_0to5    : num  12.27 7.86 13.67 12.5 12.77 ...
##  $ area       : num  0.993 1 0.956 0.98 0.95 ...
##  $ dts        : num  1920 1920 1920 1920 1920 ...
##  $ sample_rad : int  202 37 376 225 276 294 454 97 408 317 ...
```

4. Make models for different dts categorisation and categorise dts according to result.

```
## Reduce nest_ALS to one sample radius
D <- nest_ALS[nest_ALS$sample_rad == 15, ]

## Make loop through different categorisation distances and store results
r.dts_cat <- NULL
for(i in seq(500, 3500, 50)) {

  ## Categorise dts
  D$dts_cat <- ifelse(D$dts > i, "far", "close")

  m.dts_cat <- glmer(rep_succ ~ dts_cat + hab_qual + (1|female_ring) + (1|male_ring) + (1|year
),
```

```r
                     family = binomial,
                     data = D,
                     control = cont_spec)

  ## Store model output for all categorisation distances
  r.dts_cat <- rbind(r.dts_cat, cbind("AIC" = AIC(m.dts_cat), "dts_cat" = i))

}

head(r.dts_cat)
```

```
##           AIC dts_cat
## [1,] 322.0492     500
## [2,] 321.1020     550
## [3,] 321.5285     600
## [4,] 321.3227     650
## [5,] 321.3227     700
## [6,] 319.7054     750
```

```r
## Test model assumptions with DHARMa for chosen dts_cat

## Select dts_cat with lowest Estimate
R <- r.dts_cat[r.dts_cat[,"AIC"] == min(r.dts_cat[,"AIC"]), "dts_cat"]
print(R)
```

```
## dts_cat
##    1450
```

```r
D$dts_cat <- ifelse(D$dts > R, "far", "close")

m.dts_cat <- glmer(rep_succ ~ dts_cat + hab_qual + (1|female_ring) + (1|male_ring) + (1|year),

                   family = binomial,
                   data = D,
                   control = cont_spec)

test_my_model(m.dts_cat)
```

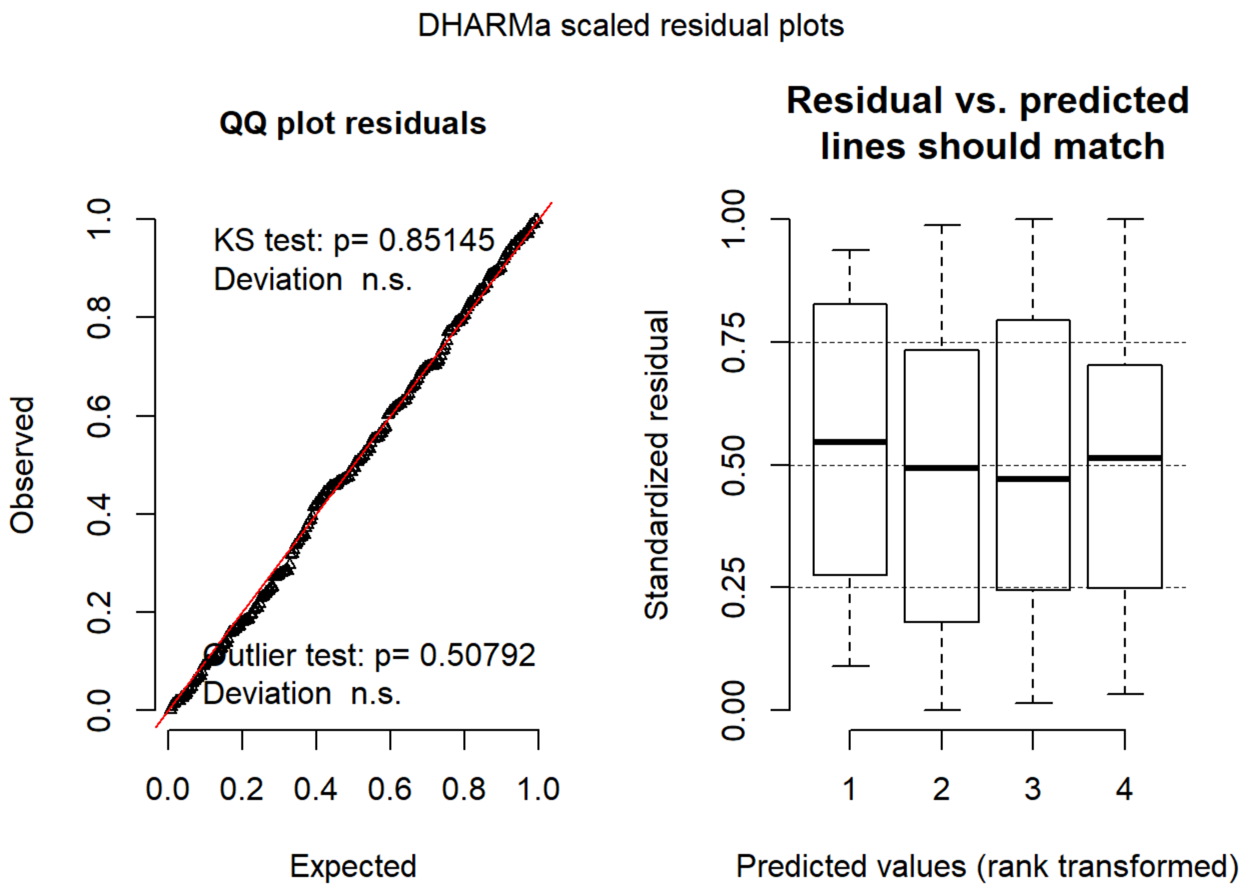
DHARMa scaled residual plots
QQ plot residuals
KS test: p= 0.85145
Deviation n.s.
Outlier test: p= 0.50792
Deviation n.s.
Observed
Expected
0.0
0.2
0.4
0.6
0.8
1.0
Residual vs. predicted
lines should match
Standardized residual
Predicted values (rank transformed)
0.00
0.25
0.50
0.75
1.00
1
2
3
4

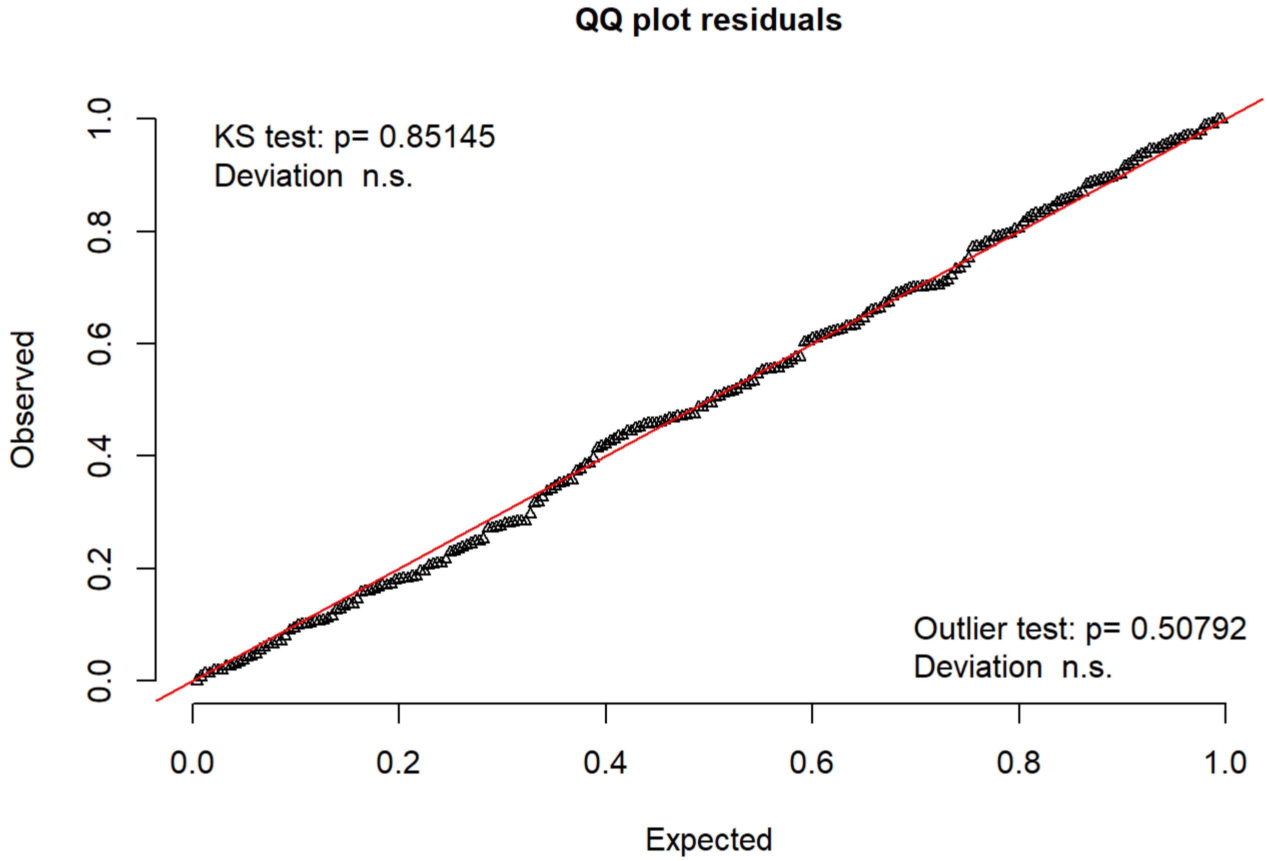
QQ plot residuals
KS test: p= 0.85145
Deviation n.s.
Outlier test: p= 0.50792
Deviation n.s.
Observed
Expected
0.0
0.2
0.4
0.6
0.8
1.0

```
##
##  One-sample Kolmogorov-Smirnov test
##
## data:  simulationOutput$scaledResiduals
## D = 0.03902, p-value = 0.8514
## alternative hypothesis: two-sided
```

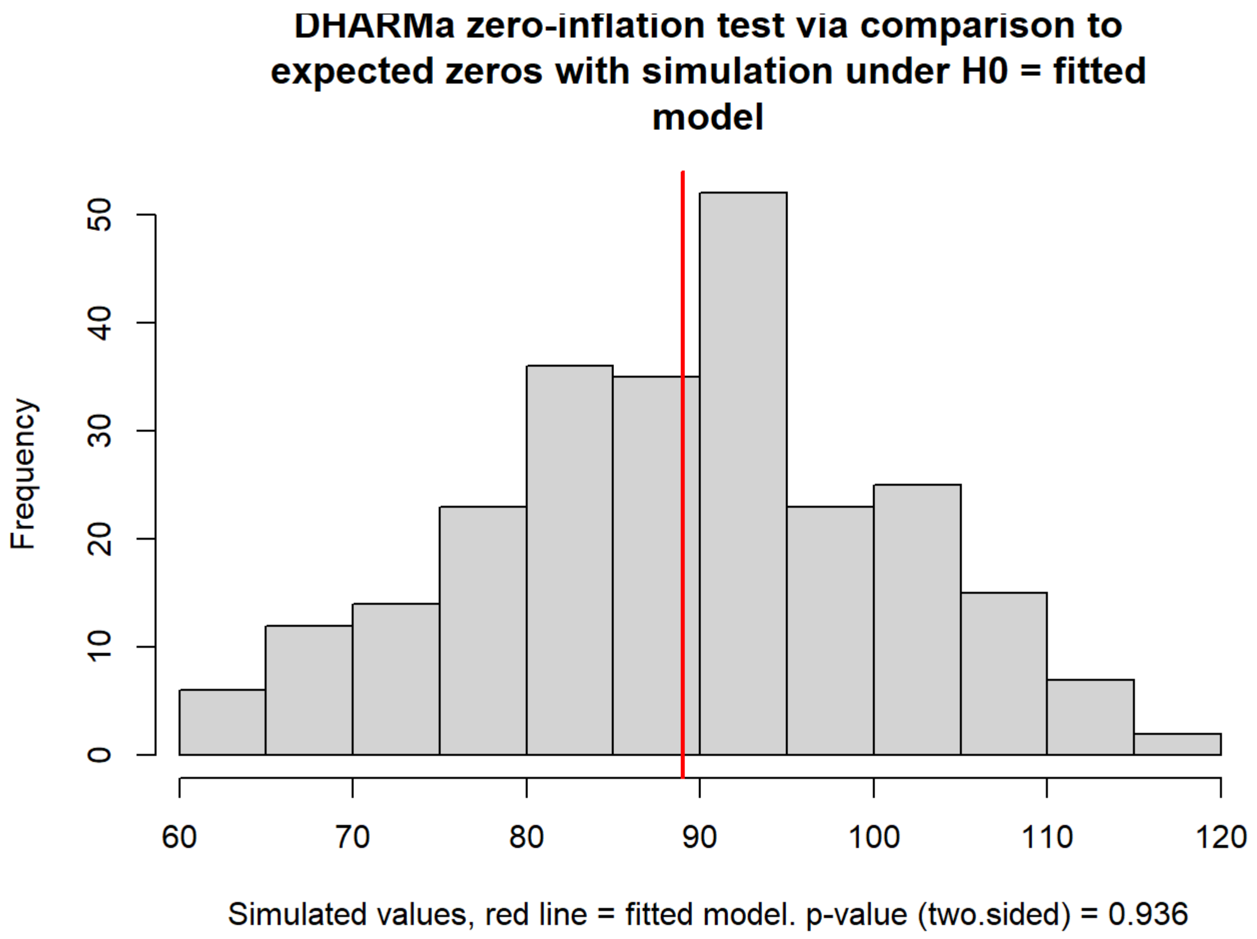


```
##
##  DHARMa zero-inflation test via comparison to expected zeros with
##  simulation under H0 = fitted model
##
## data:  simulationOutput
## ratioObsSim = 0.99242, p-value = 0.936
## alternative hypothesis: two.sided
```

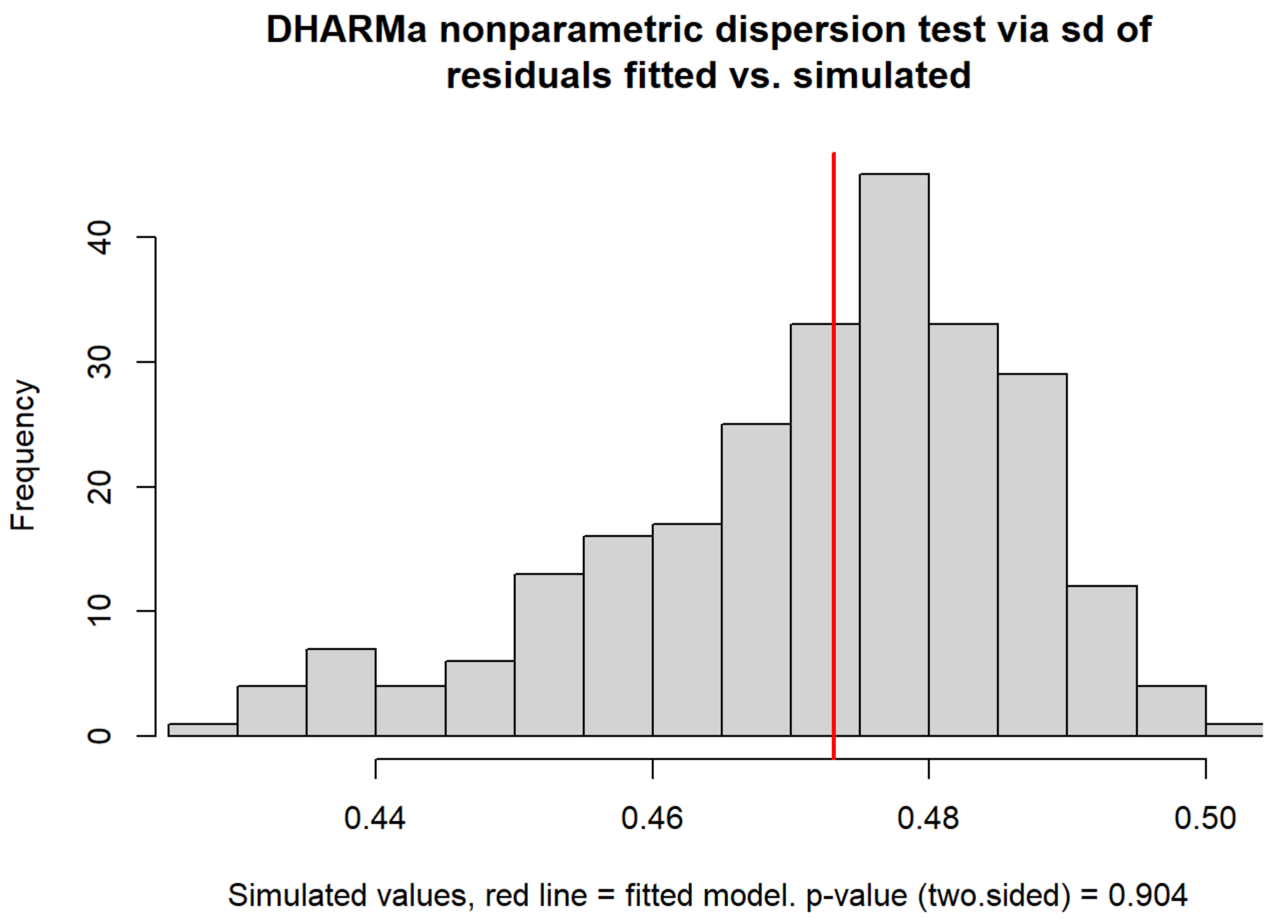


```
##
##  DHARMa nonparametric dispersion test via sd of residuals fitted
##  vs. simulated
##
## data:  simulationOutput
## ratioObsSim = 1.0027, p-value = 0.904
## alternative hypothesis: two.sided
##
## [1] "Variance Inflation Factor:"
##  dts_cat hab_qual
## 1.262607 1.262607
```

5. Make the main model testing ALS on breeding success for 15 m radius around the nest, beacuse most data for that one. Test alternative (linear and quadratic instead of logarithmic) relationships between understory density and breeding success with AIC.

```
## Categorise dts according to the results above in nest_ALS
nest_ALS$dts_cat <- ifelse(nest_ALS$dts > R, "far", "close")

## Exclude NA's in data set for use in models below
DD <- na.omit(nest_ALS)

## Reduce DD to D15
D15 <- DD[DD$sample_rad == 15, ]
```

```r
## Add logarithmic version of vd_0to5
D15$vd0t5_log <- log(D15$vd_0to5)

## Center all continuous variables to avoid covariate correlations
D15$vd0t5_log_c <- D15$vd0t5_log - mean(D15$vd0t5_log)
D15$vd0t5_c <- D15$vd_0to5 - mean(D15$vd_0to5)

## Test log model with veg density as a logarithmic predictor

m.vd0t5_log_c_15 <- glmer(rep_succ ~ dts_cat * vd0t5_log_c + hab_qual + (1|female_ring) + (1|m
ale_ring) + (1|year),
                              family = binomial,
                              data = D15,
                              control = cont_spec)

summary(m.vd0t5_log_c_15)
```

```
## Generalized linear mixed model fit by maximum likelihood (Laplace
##   Approximation) [glmerMod]
##  Family: binomial  ( logit )
## Formula:
## rep_succ ~ dts_cat * vd0t5_log_c + hab_qual + (1 | female_ring) +
##     (1 | male_ring) + (1 | year)
##    Data: D15
## Control: cont_spec
##
##      AIC      BIC   logLik deviance df.resid
##    301.4    329.1   -142.7    285.4      227
##
## Scaled residuals:
##     Min      1Q  Median      3Q     Max
## -2.4643 -0.9446  0.5007  0.7026  1.7395
##
## Random effects:
##  Groups      Name        Variance Std.Dev.
##  male_ring   (Intercept) 0.0000   0.0000
##  female_ring (Intercept) 0.0000   0.0000
##  year        (Intercept) 0.3293   0.5739
## Number of obs: 235, groups:  male_ring, 137; female_ring, 130; year, 10
##
## Fixed effects:
##                         Estimate Std. Error z value Pr(>|z|)
## (Intercept)               0.2411     0.2972   0.811  0.41721
## dts_catfar                1.0427     0.3504   2.976  0.00292 **
## vd0t5_log_c               0.3965     0.3053   1.298  0.19415
## hab_qualunmanaged        -0.5196     0.3550  -1.464  0.14330
## dts_catfar:vd0t5_log_c  -1.5702     0.6046  -2.597  0.00939 **
## ---
## Signif. codes:  0 '***' 0.001 '**' 0.01 '*' 0.05 '.' 0.1 ' ' 1
##
## Correlation of Fixed Effects:
##             (Intr) dts_ct vd05__ hb_qln
## dts_catfar  -0.417
```

```
## vd0t5_log_c  0.151 -0.026
## hab_qlnmngd -0.118 -0.476 -0.094
## dts_ct:05__ -0.076 -0.206 -0.501  0.120
## convergence code: 0
## boundary (singular) fit: see ?isSingular
```

```
r.squaredGLMM(m.vd0t5_log_c_15)
```

```
##                    R2m       R2c
## theoretical 0.09616902 0.1784091
## delta       0.07707160 0.1429803
```

```
test_my_model(m.vd0t5_log_c_15)
```

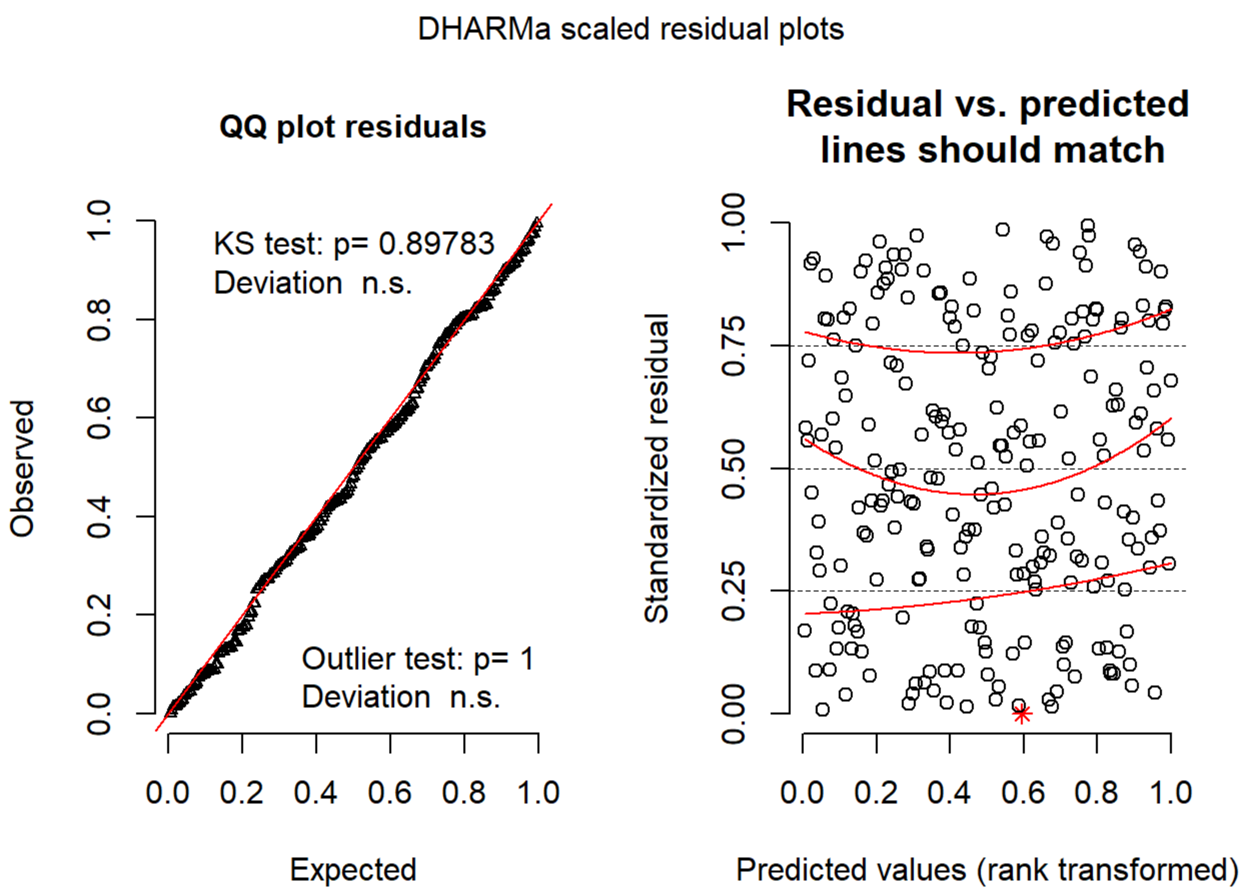

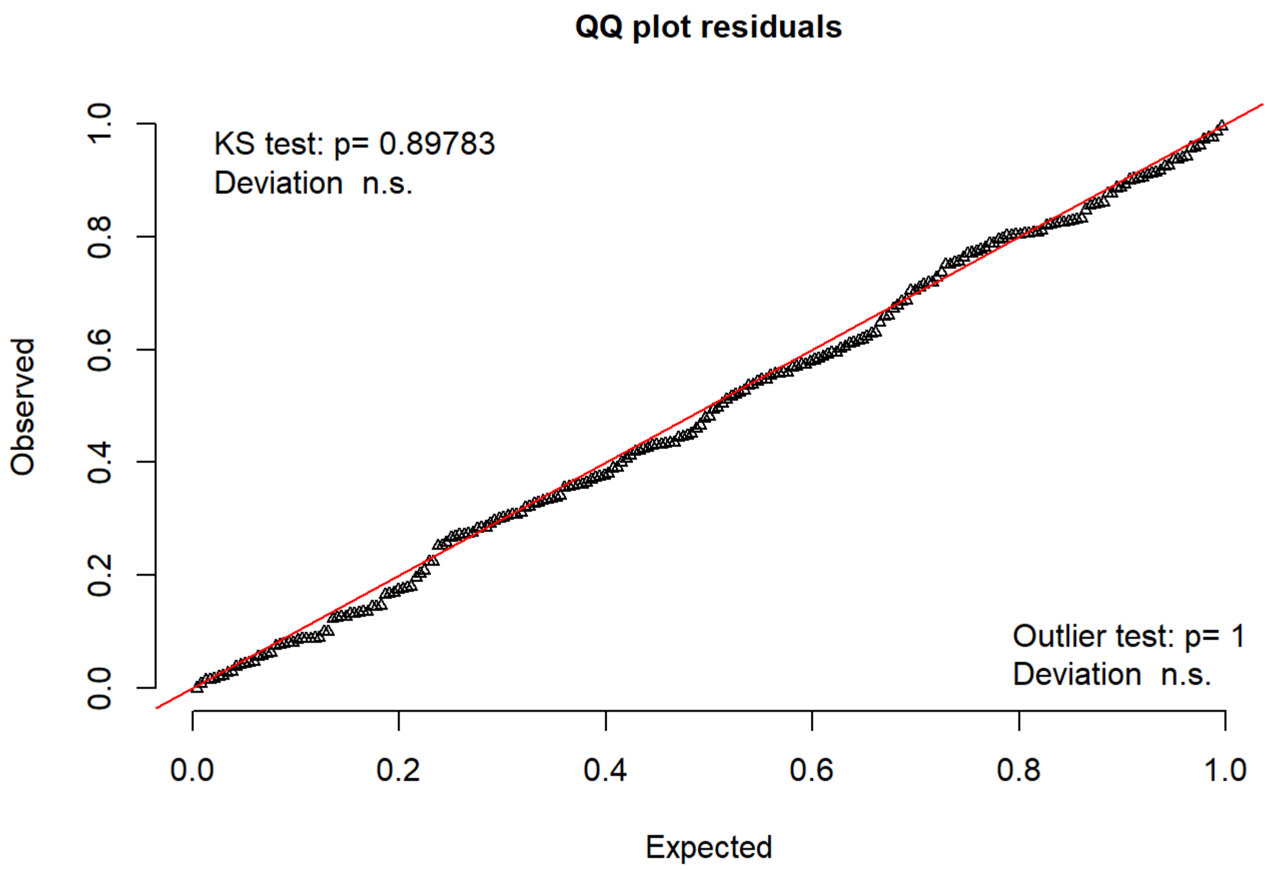


```
##
##  One-sample Kolmogorov-Smirnov test
##
## data:  simulationOutput$scaledResiduals
## D = 0.037382, p-value = 0.8978
## alternative hypothesis: two-sided
```

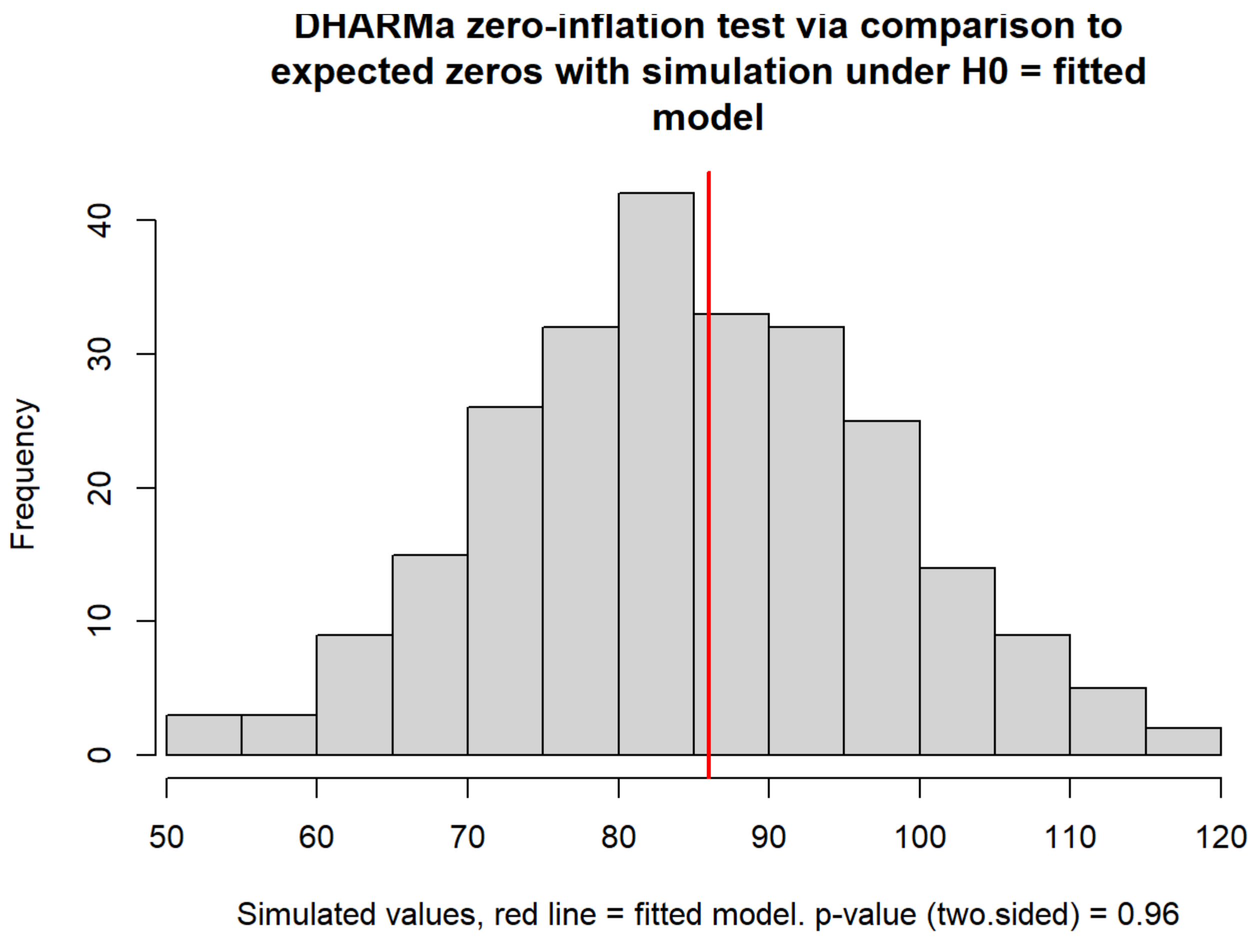


```
##
##  DHARMa zero-inflation test via comparison to expected zeros with
##  simulation under H0 = fitted model
##
## data:  simulationOutput
## ratioObsSim = 1.0079, p-value = 0.96
## alternative hypothesis: two.sided
```

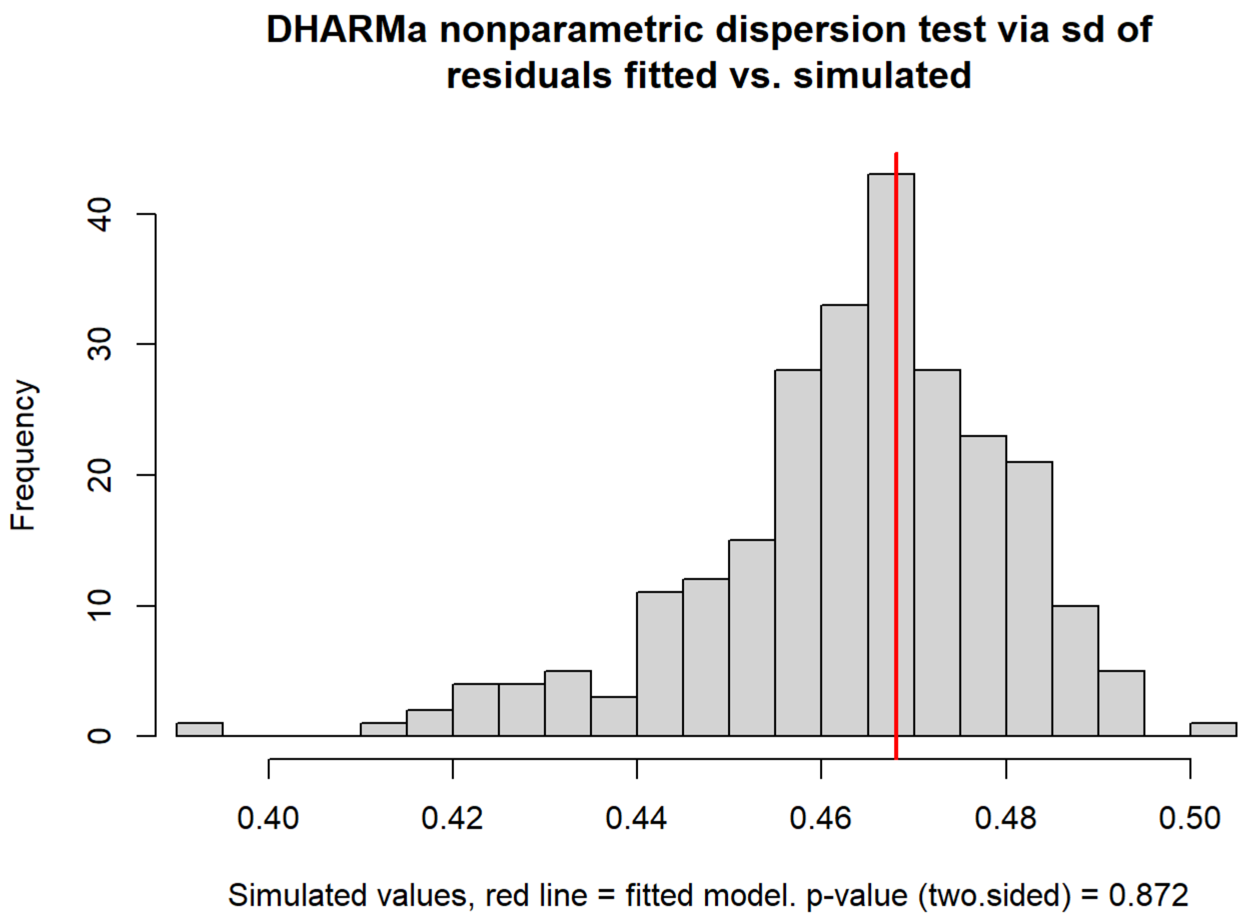


```
##
##  DHARMa nonparametric dispersion test via sd of residuals fitted
##  vs. simulated
##
## data:  simulationOutput
## ratioObsSim = 1.0096, p-value = 0.872
## alternative hypothesis: two.sided
##
## [1] "Variance Inflation Factor:"
##              dts_cat          vd0t5_log_c             hab_qual
##             1.383052             1.388480             1.314170
## dts_cat:vd0t5_log_c
##             1.428788
```

```
## Store data set with predictions for figure 1 which is produced below
newdata <- expand.grid.df(D15[, c("name", "rep_succ", "dts_cat", "vd0t5_log_c", "vd_0to5")],
                          data.frame("hab_qual" = levels(D15$hab_qual)))

fig1 <- cbind(predict(m.vd0t5_log_c_15, newdata = newdata, re.form = NA, se.fit = TRUE, type =
 "response"),
              newdata)

## Compare to quadratic, linear and intercep only model

## Only the intercept:
```

```
m.int <- glmer(rep_succ ~ (1|female_ring) + (1|male_ring) + (1|year),
               family = binomial,
               data = D15,
               control = cont_spec)

## Only the habitat quality:
m.hq <- glmer(rep_succ ~ hab_qual + (1|female_ring) + (1|male_ring) + (1|year),
              family = binomial,
              data = D15,
              control = cont_spec)

## Distance to settlement only:
m.dts_cat_15 <- glmer(rep_succ ~ dts_cat + hab_qual + (1|female_ring) + (1|male_ring) + (1|yea
r),
                      family = binomial,
                      data = D15,
                      control = cont_spec)

## With veg density only as a linear predictor:
m.vd0t5_c_15_only <- glmer(rep_succ ~ vd0t5_c + hab_qual + (1|female_ring) + (1|male_ring) + (
1|year),
                           family = binomial,
                           data = D15,
                           control = cont_spec)
## Test model assumptions:
test_my_model(m.vd0t5_c_15_only)
```

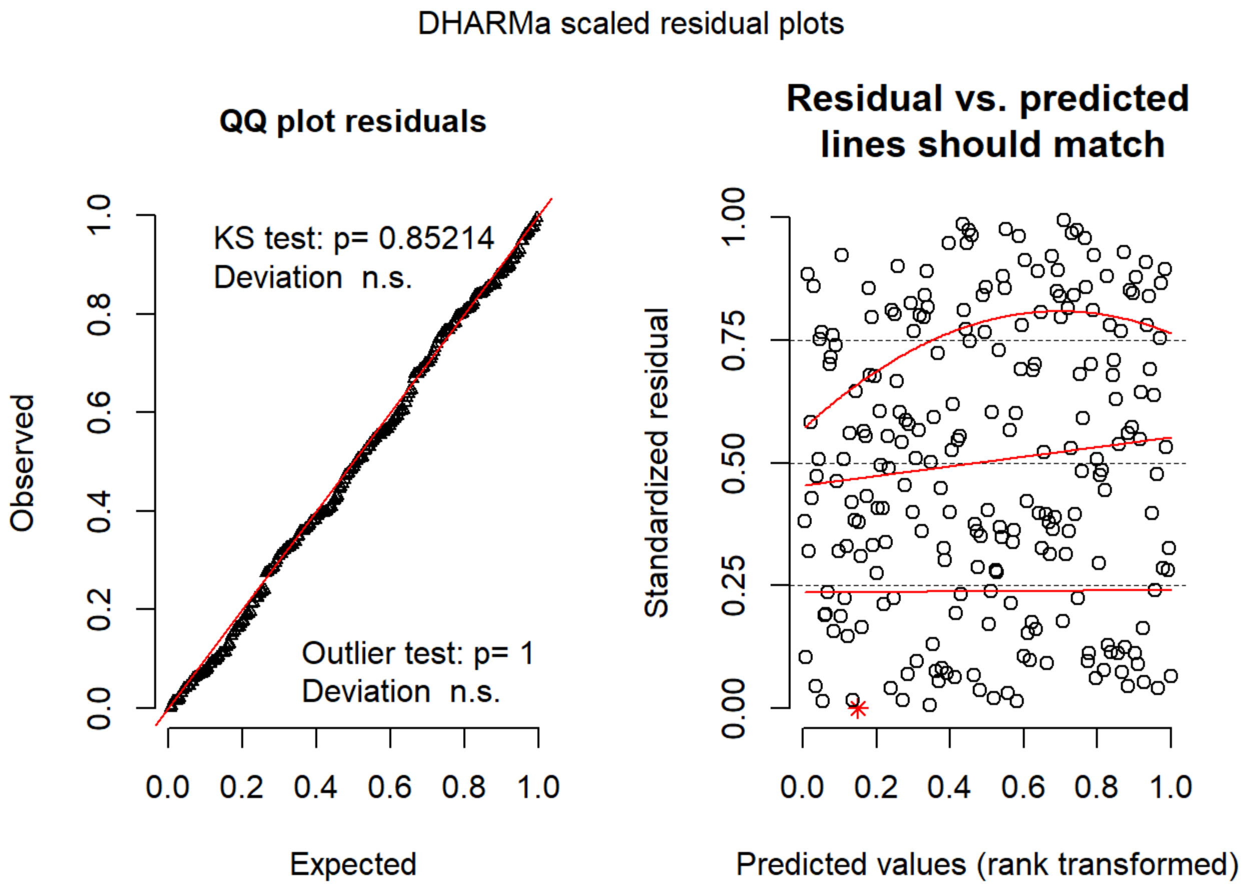

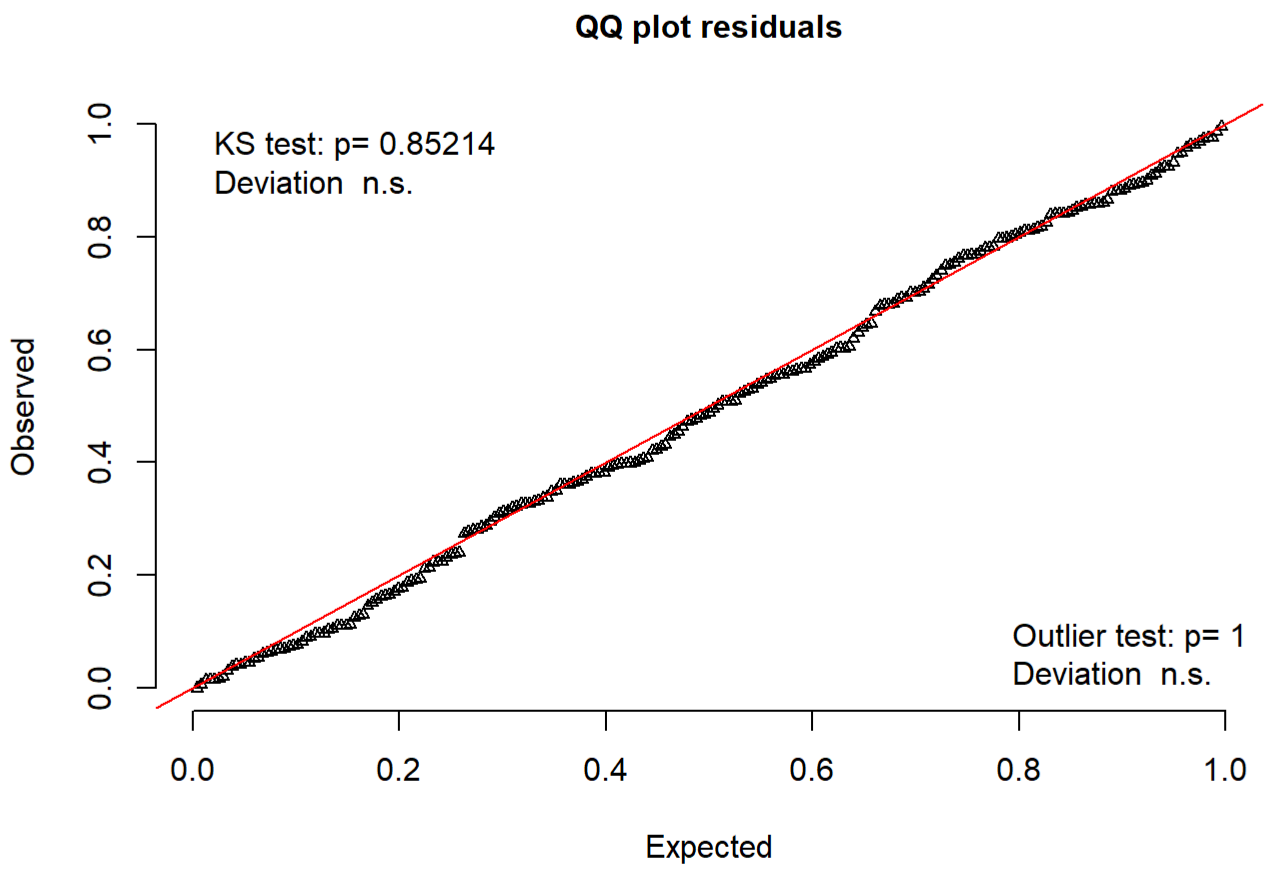


```
##
##  One-sample Kolmogorov-Smirnov test
##
## data:  simulationOutput$scaledResiduals
## D = 0.039727, p-value = 0.8521
## alternative hypothesis: two-sided
```

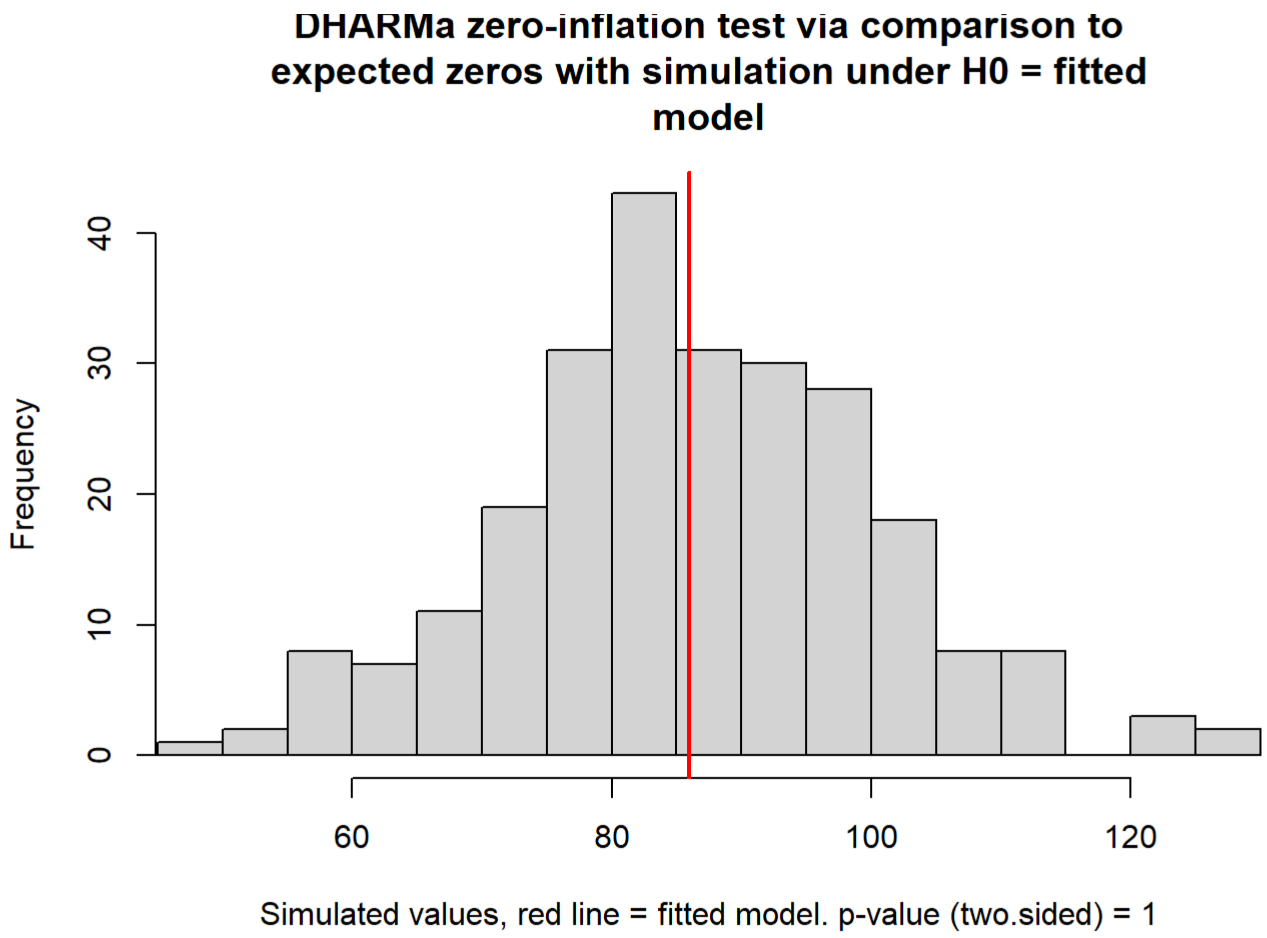


```
##
##  DHARMa zero-inflation test via comparison to expected zeros with
##  simulation under H0 = fitted model
##
## data:  simulationOutput
## ratioObsSim = 0.99078, p-value = 1
## alternative hypothesis: two.sided
```

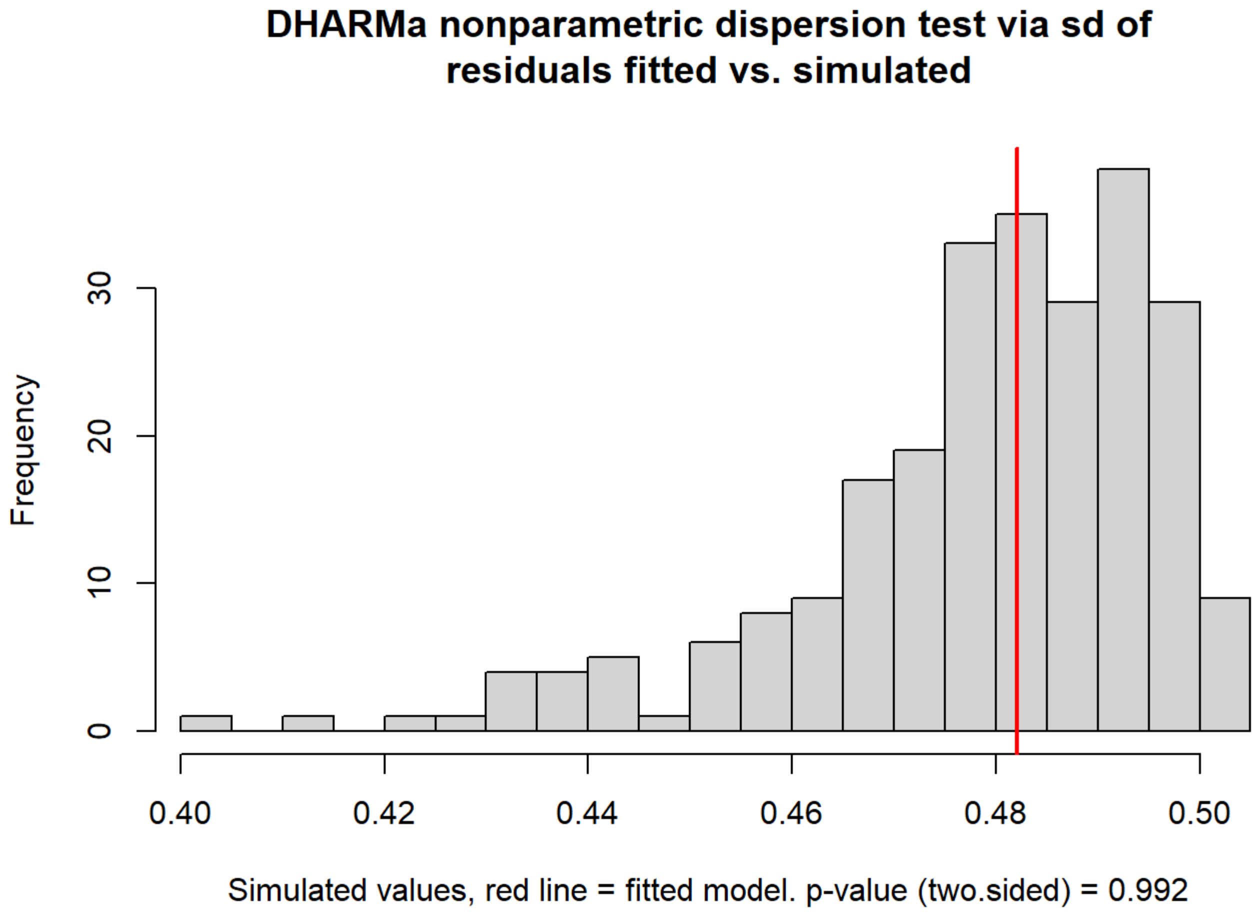


```
##
##  DHARMa nonparametric dispersion test via sd of residuals fitted
##  vs. simulated
##
## data:  simulationOutput
## ratioObsSim = 1.0065, p-value = 0.992
## alternative hypothesis: two.sided
##
## [1] "Variance Inflation Factor:"
##  vd0t5_c hab_qual
##  1.00149  1.00149
```

```
## With veg density as a linear predictor in interaction with dts:
m.vd0t5_c_15 <- glmer(rep_succ ~ dts_cat * vd0t5_c + hab_qual + (1|female_ring) + (1|male_ring
) + (1|year),
                          family = binomial,
                          data = D15,
                          control = cont_spec)
## Test model assumptions:
test_my_model(m.vd0t5_c_15)
```

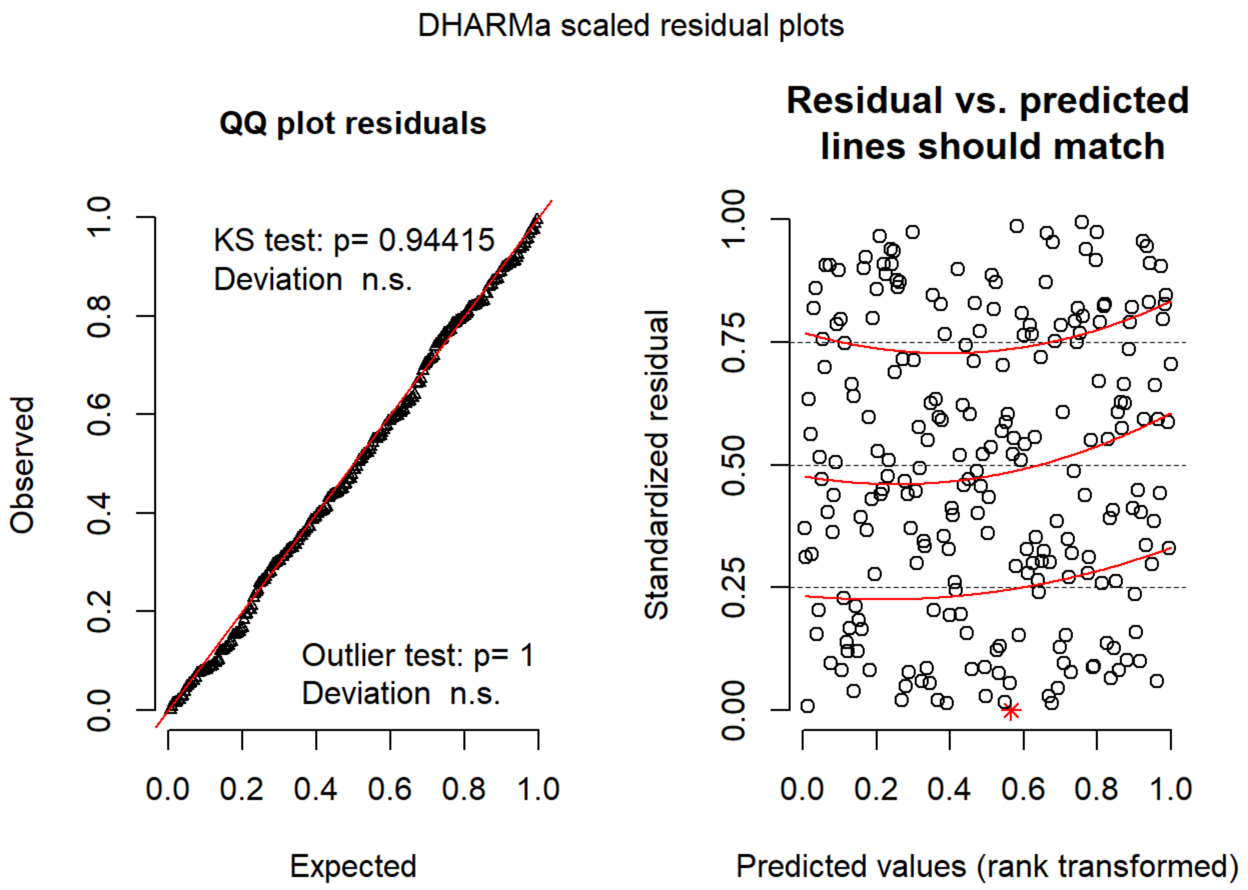
DHARMa scaled residual plots
QQ plot residuals
KS test: p= 0.94415
Deviation n.s.
Outlier test: p= 1
Deviation n.s.
Observed
Expected
0.0
0.2
0.4
0.6
0.8
1.0
Residual vs. predicted
lines should match
Standardized residual
0.00
0.25
0.50
0.75
1.00
Predicted values (rank transformed)

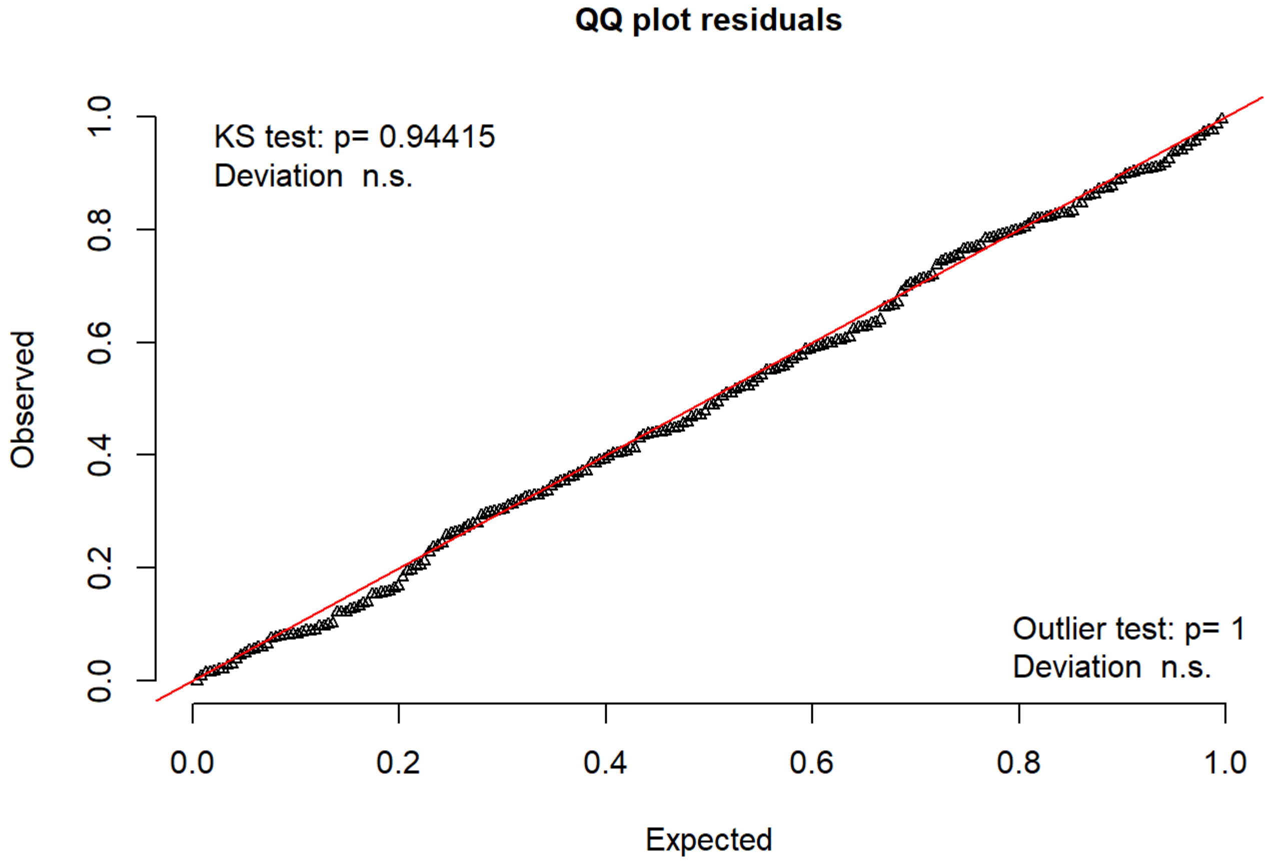
QQ plot residuals
KS test: p= 0.94415
Deviation n.s.
Outlier test: p= 1
Deviation n.s.
Observed
Expected
0.0
0.2
0.4
0.6
0.8
1.0

```
##
##  One-sample Kolmogorov-Smirnov test
##
## data:  simulationOutput$scaledResiduals
## D = 0.034367, p-value = 0.9442
## alternative hypothesis: two-sided
```

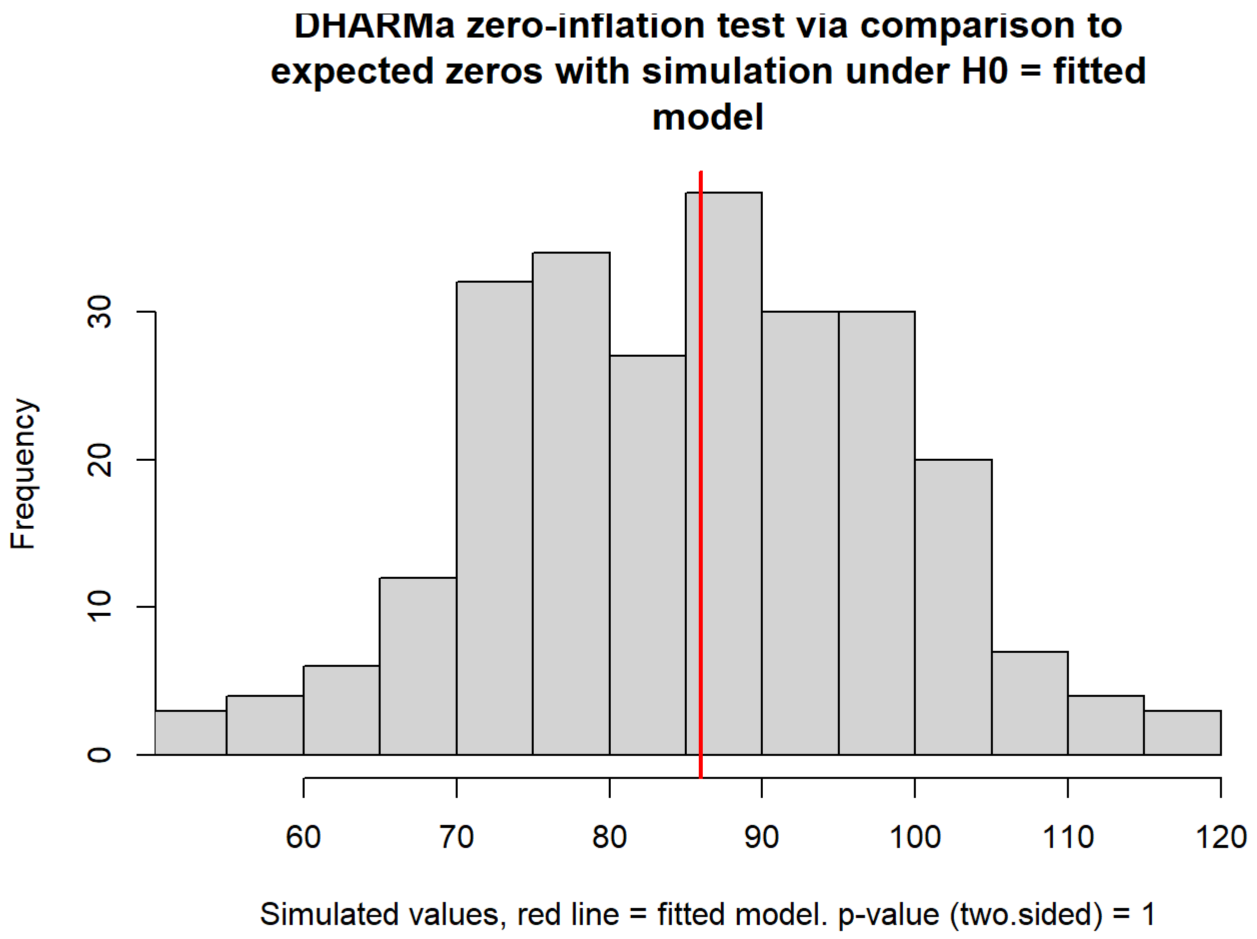


```
##
##  DHARMa zero-inflation test via comparison to expected zeros with
##  simulation under H0 = fitted model
##
## data:  simulationOutput
## ratioObsSim = 0.99833, p-value = 1
## alternative hypothesis: two.sided
```

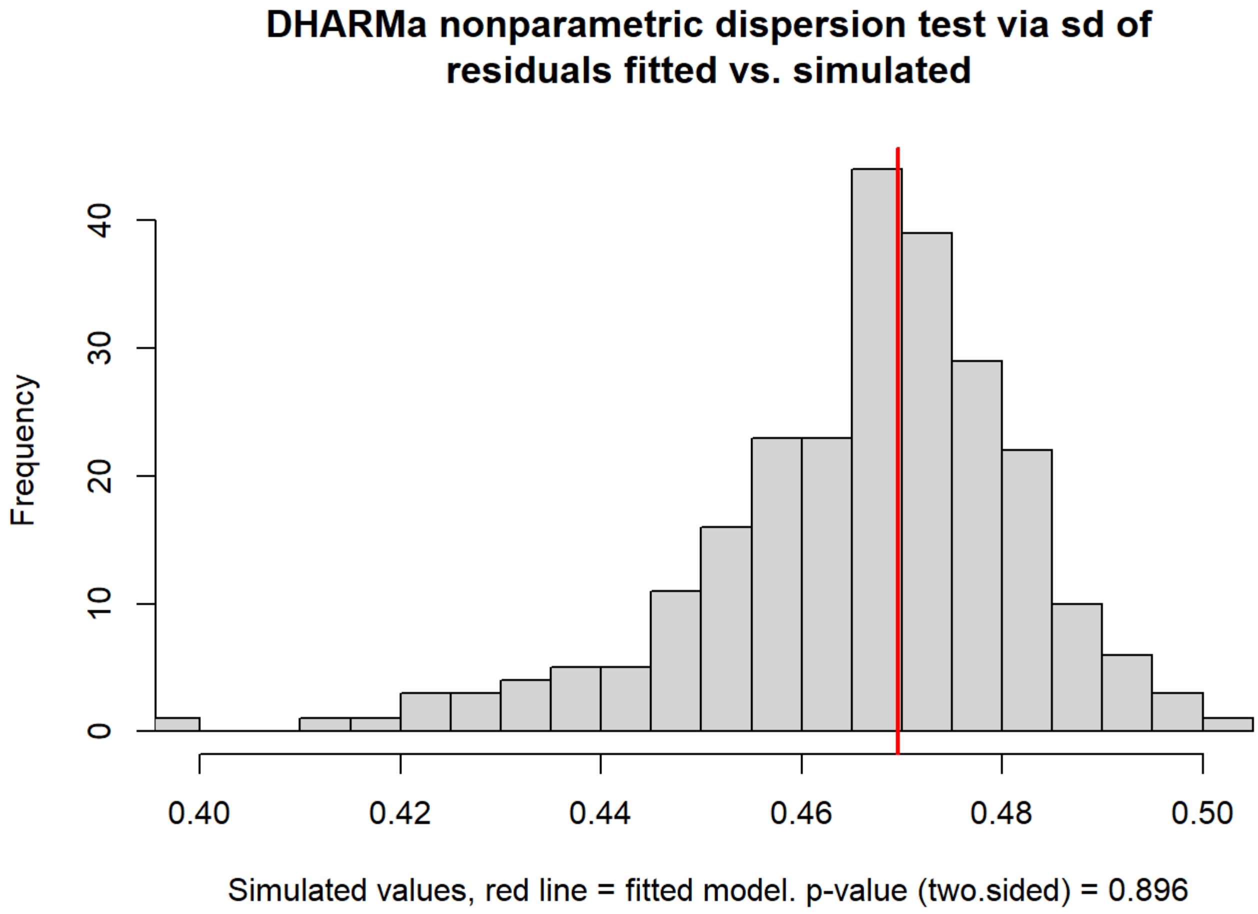


```
##
##  DHARMa nonparametric dispersion test via sd of residuals fitted
##  vs. simulated
##
## data:  simulationOutput
## ratioObsSim = 1.0073, p-value = 0.896
## alternative hypothesis: two.sided
##
## [1] "Variance Inflation Factor:"
##          dts_cat         vd0t5_c        hab_qual dts_cat:vd0t5_c
##         1.327256        1.613872        1.328984        1.646759
```

```
## With veg density as a quadratic predictor:
m.vd0t5_c_poly_15 <- glmer(rep_succ ~ dts_cat * poly(vd0t5_c, 2) + hab_qual + (1|female_ring)
+ (1|male_ring) + (1|year),
                               family = binomial,
                               data = D15,
                               control = cont_spec)
## Test model assumptions:
test_my_model(m.vd0t5_c_15)
```

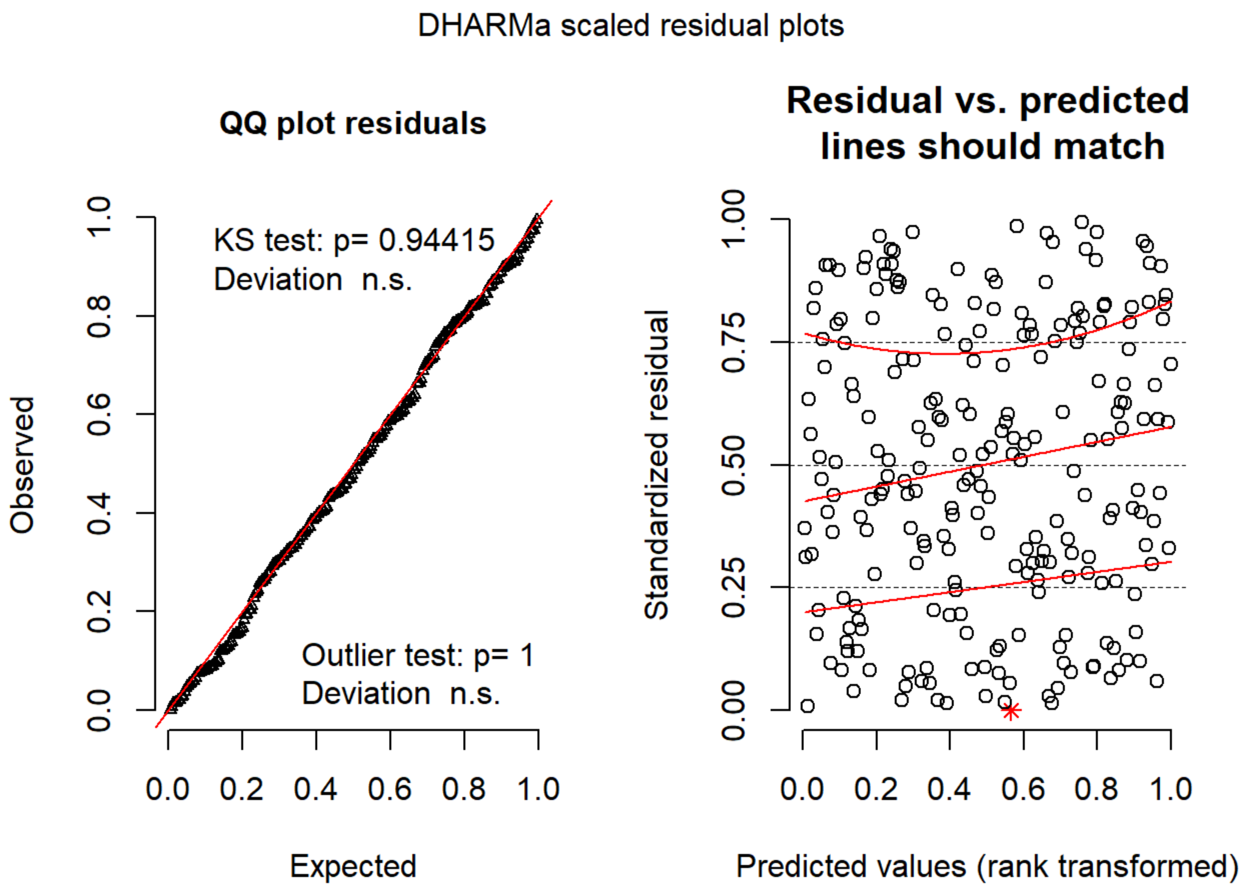
DHARMa scaled residual plots
QQ plot residuals
KS test: p= 0.94415
Deviation n.s.
Outlier test: p= 1
Deviation n.s.
Observed
Expected
Residual vs. predicted
lines should match
Standardized residual
Predicted values (rank transformed)

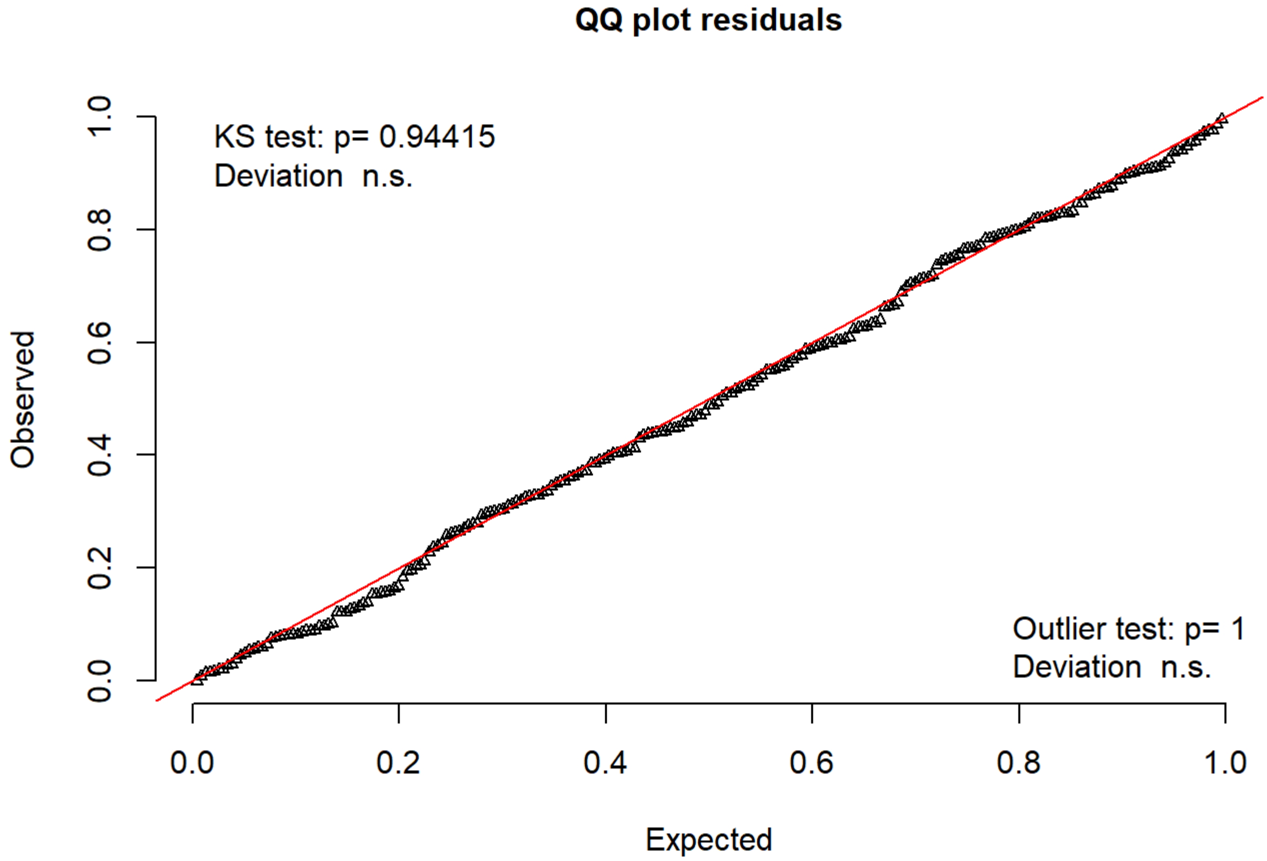
QQ plot residuals
KS test: p= 0.94415
Deviation n.s.
Outlier test: p= 1
Deviation n.s.
Observed
Expected

```
##
##  One-sample Kolmogorov-Smirnov test
##
## data:  simulationOutput$scaledResiduals
## D = 0.034367, p-value = 0.9442
## alternative hypothesis: two-sided
```

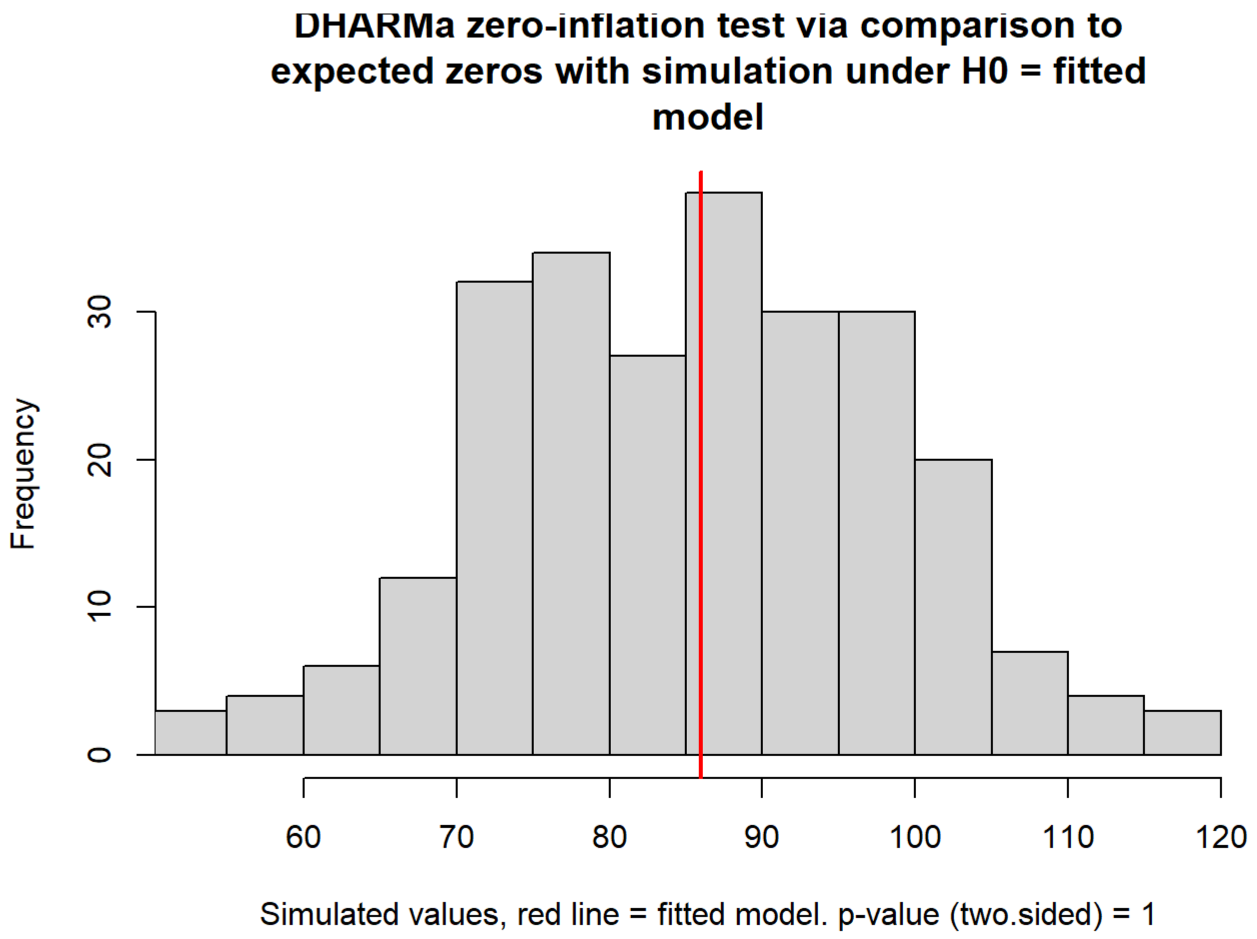


```
##
##  DHARMa zero-inflation test via comparison to expected zeros with
##  simulation under H0 = fitted model
##
## data:  simulationOutput
## ratioObsSim = 0.99833, p-value = 1
## alternative hypothesis: two.sided
```

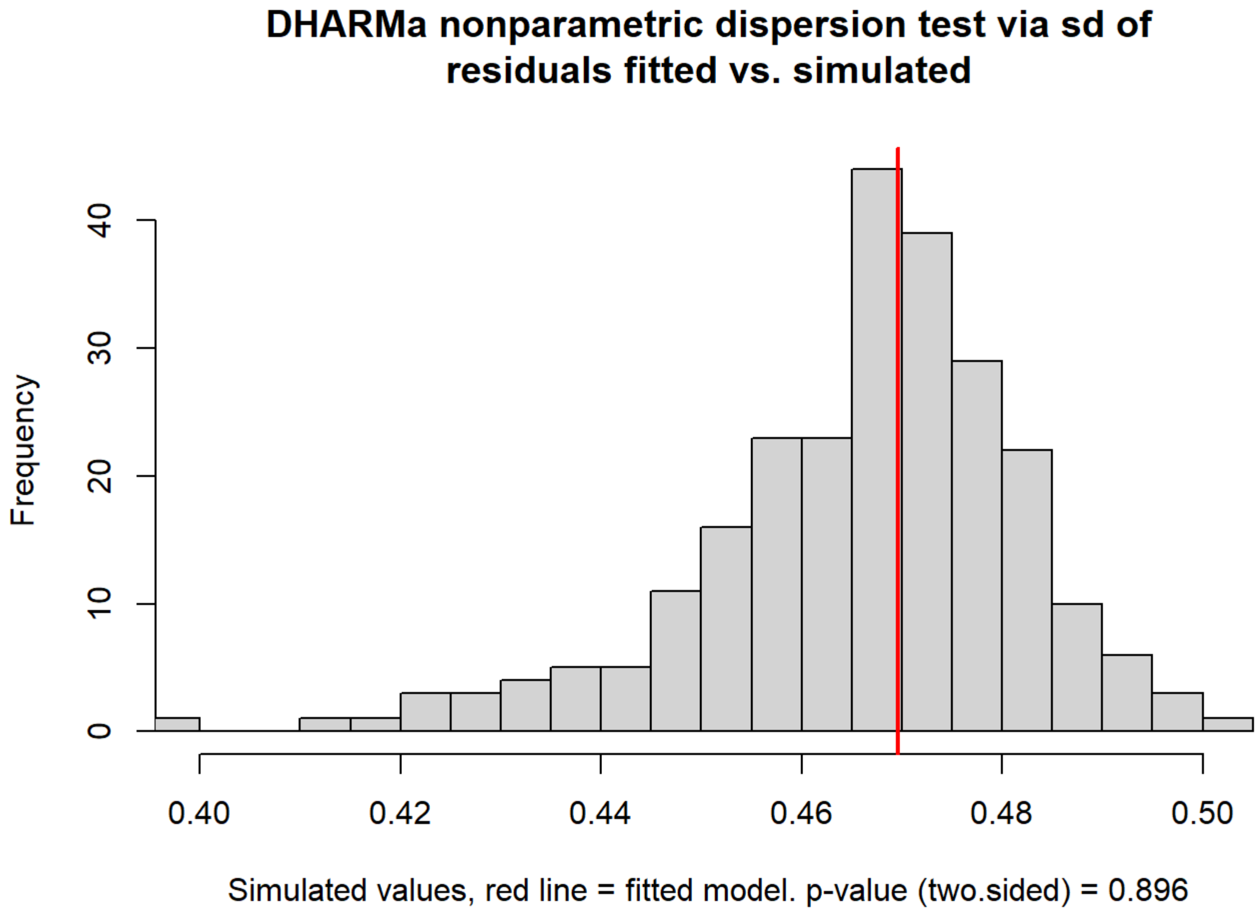


```
##
##  DHARMa nonparametric dispersion test via sd of residuals fitted
##  vs. simulated
##
## data:  simulationOutput
## ratioObsSim = 1.0073, p-value = 0.896
## alternative hypothesis: two.sided
##
## [1] "Variance Inflation Factor:"
##        dts_cat        vd0t5_c        hab_qual dts_cat:vd0t5_c
##       1.327256       1.613872        1.328984        1.646759
```

```
## List all models above in a model selection table and export results:
model.sel(m.vd0t5_log_c_15, m.hq, m.int, m.dts_cat_15, m.vd0t5_c_15_only, m.vd0t5_c_15, m.vd0t
5_c_poly_15)
```

```
## Model selection table
##                   (Int) dts_cat hab_qul v05_log_c dts_cat:v05_log_c
## m.vd0t5_log_c_15  0.2411       +       +    0.3965                 +
## m.vd0t5_c_15      0.2023       +       +
## m.vd0t5_c_poly_15 0.3095       +       +
## m.dts_cat_15      0.1860       +       +
## m.int             0.5652
## m.hq              0.5725               +
## m.vd0t5_c_15_only 0.5617               +
```

```
##                      v05_c dts_cat:v05_c ply(v05_c,2) dts_cat:ply(v05_c,2)
## m.vd0t5_log_c_15
## m.vd0t5_c_15       0.02439             +
## m.vd0t5_c_poly_15                                    +                    +
## m.dts_cat_15
## m.int
## m.hq
## m.vd0t5_c_15_only -0.01760
##                   df   logLik  AICc delta weight
## m.vd0t5_log_c_15   8 -142.697 302.0  0.00  0.566
## m.vd0t5_c_15       8 -143.877 304.4  2.36  0.174
## m.vd0t5_c_poly_15 10 -142.066 305.1  3.08  0.121
## m.dts_cat_15       6 -146.444 305.3  3.22  0.113
## m.int              4 -150.409 309.0  6.96  0.017
## m.hq               5 -150.406 311.1  9.04  0.006
## m.vd0t5_c_15_only  6 -150.151 312.7 10.64  0.003
## Models ranked by AICc(x)
## Random terms (all models):
## '1 | female_ring', '1 | male_ring', '1 | year'
```

6. Check wether the results of the main model are sensitive to the categorisation distance of the distance of the nest to the closest settlement.

```
## Make loop through different categorisation distances and store results:
r.sensitivity <- NULL
for(i in seq(500, 3500, 50)) {

  ## Categorise dts
  D15$dts_cat <- ifelse(D15$dts > i, "far", "close")

  m.sensitivity <- glmer(rep_succ ~ dts_cat * vd0t5_log_c + hab_qual + (1|female_ring) + (1|ma
le_ring) + (1|year),
                         family = binomial,
                         data = D15,
                         control = cont_spec)

  ## Store model output for all categorisation distances:
  r.sensitivity <- rbind(r.sensitivity,
                         cbind(summary(m.sensitivity)$coefficients,
                               "R2m" = r.squaredGLMM(m.sensitivity)[1, 1],
                               "AIC" = AIC(m.sensitivity),
                               "dts_cat" = i))

}

head(r.sensitivity)
```

```
##                            Estimate Std. Error     z value  Pr(>|z|)
## (Intercept)             -0.16371604  0.5673451 -0.28856517 0.7729142
## dts_catfar               0.81390402  0.5558269  1.46431198 0.1431087
## vd0t5_log_c              0.04380957  0.5039813  0.08692698 0.9307296
## hab_qualunmanaged       -0.06772085  0.3046750 -0.22227246 0.8241018
```

```
## dts_catfar:vd0t5_log_c -0.06986914  0.5737804 -0.12176984 0.9030813
## (Intercept)             -0.13499688  0.4925137 -0.27409773 0.7840095
##                                R2m      AIC dts_cat
## (Intercept)             0.01193296 314.5195     500
## dts_catfar              0.01193296 314.5195     500
## vd0t5_log_c             0.01193296 314.5195     500
## hab_qualunmanaged       0.01193296 314.5195     500
## dts_catfar:vd0t5_log_c 0.01193296 314.5195     500
## (Intercept)             0.01913569 313.2259     550
```

7. Test the logarithmic model with understory density for all radiuses around the nest and extract AIC values. Also calculate the correlation of the mean vd_0to5 of all radiuses with 15 m.

```
## Reduce data set so the same nests are used for all radiuses:

## Which radius has fewest nests?
N1 <- names(sort(table(DD$sample_rad)))[1]

## Which nests are those?
N2 <- DD[DD$sample_rad == N1 & !is.na(DD$vd_0to5), "name"]

## Select only those nests:
DD_all_rad <- DD[DD$name %in% N2, ]

## Add centered log(vd_0to5) by radius:
DD_all_rad <- as.data.table(DD_all_rad)
DD_all_rad[, "vd0t5_log_c" := log(vd_0to5) - mean(log(vd_0to5)), by = "sample_rad"]

## Make a loop through all radiuses, run the log model and store results:

r.all_rad <- NULL
for(i in unique(DD_all_rad$sample_rad)) {

  tryCatch({

    ## Calculate correlation of vd_0to5 at all radiuses with 15m:
    r.cor <- cor(DD_all_rad[DD_all_rad$sample_rad == i, "vd_0to5"],
                 DD_all_rad[DD_all_rad$sample_rad == 15, "vd_0to5"])

    m.all_rad <- glmer(rep_succ ~ dts_cat * vd0t5_log_c + area + hab_qual + (1|female_ring) +
(1|male_ring) + (1|year),
                       family = binomial,
                       data = DD_all_rad[DD_all_rad$sample_rad == i, ],
                       control = cont_spec)

    ## Store model output for all radiuses:
    r.all_rad <- rbind(r.all_rad,
                       cbind("cor" = r.cor[1],
                             "estimate" = summary(m.all_rad)$coefficients[5, 1],
                             "SE" = summary(m.all_rad)$coefficients[5, 2],
                             "pvalue" = summary(m.all_rad)$coefficients[5, 4],
                             "AIC" = AIC(m.all_rad),
                             "R2m" = r.squaredGLMM(m.all_rad)[1, 1],
```

```
                             "radius" = i))

  }, error = function(e) {cat("ERROR :", conditionMessage(e), "\n")})

}

## Add deltaAIC to results:
r.all_rad <- as.data.frame(r.all_rad)
r.all_rad$deltaAIC <- r.all_rad$AIC - min(r.all_rad$AIC)

print(r.all_rad)
```

```
##           cor   estimate        SE    pvalue      AIC        R2m radius
## 1  0.6292844 -0.5063462 0.3776122 0.1799478 265.9985 0.06878941    202
## 2  0.9265132 -0.5011742 0.3777968 0.1846506 261.0075 0.11524156     37
## 3  0.5139351 -0.5991089 0.3873726 0.1219607 265.1539 0.07600524    376
## 4  0.6049316 -0.5132911 0.3786965 0.1752852 266.0260 0.06862476    225
## 5  0.5658906 -0.5546456 0.3801025 0.1445100 265.6853 0.07130657    276
## 6  0.5525039 -0.5650852 0.3809369 0.1379658 265.5493 0.07242097    294
## 7  0.4915881 -0.5962115 0.3913466 0.1276369 265.5287 0.07330760    454
## 8  0.7822746 -0.5042544 0.3782356 0.1824742 262.0801 0.10362790     97
## 9  0.5032371 -0.6035807 0.3899287 0.1216402 265.2086 0.07572721    408
## 10 0.5396599 -0.5767748 0.3825386 0.1316170 265.3564 0.07407497    317
## 11 0.5475871 -0.5692263 0.3814129 0.1355904 265.4949 0.07289037    303
## 12 0.4926194 -0.5978186 0.3913251 0.1265926 265.4966 0.07356863    450
## 13 0.5155729 -0.5992108 0.3871263 0.1216600 265.1080 0.07631513    372
## 14 0.5023268 -0.6041692 0.3902929 0.1216249 265.2460 0.07545989    413
## 15 0.5177933 -0.5964189 0.3865603 0.1228582 265.1559 0.07592751    367
## 16 0.5694991 -0.5507841 0.3799069 0.1471179 265.7495 0.07085154    271
## 17 0.5504851 -0.5676793 0.3812810 0.1365206 265.4992 0.07282841    298
## 18 0.4986501 -0.6022772 0.3908816 0.1233612 265.3408 0.07475166    427
## 19 0.9046009 -0.4985129 0.3789116 0.1882934 260.5001 0.12061353     46
## 20 0.8866125 -0.4965442 0.3781723 0.1891795 261.1164 0.11485194     51
## 21 0.8498556 -0.4852537 0.3773858 0.1985027 261.6158 0.11191008     65
## 22 0.6483085 -0.5082961 0.3773637 0.1779912 265.7530 0.07056499    188
## 23 0.5227707 -0.5931369 0.3854749 0.1238733 265.1627 0.07574523    353
## 24 0.6236221 -0.5042245 0.3778481 0.1820520 266.0061 0.06871737    207
## 25 0.4972704 -0.6011109 0.3909748 0.1241790 265.3621 0.07459073    431
## 26 0.9146131 -0.5044490 0.3794396 0.1836969 260.1449 0.12407732     42
## 27 0.8302803 -0.4941181 0.3779576 0.1910981 261.3858 0.11269805     74
## 28 0.5866711 -0.5324661 0.3793723 0.1604543 265.8134 0.07026310    248
## 29 0.4994193 -0.6036244 0.3907222 0.1223719 265.2770 0.07522932    422
## 30 0.5566857 -0.5621962 0.3807389 0.1397849 265.5533 0.07236417    289
## 31 0.5911123 -0.5287881 0.3792459 0.1632226 265.8555 0.06993462    243
## 32 0.9461769 -0.5226965 0.3769594 0.1655600 261.3176 0.11208636     28
## 33 0.5103185 -0.6005040 0.3881841 0.1218737 265.1774 0.07590284    386
## 34 0.6411621 -0.5047524 0.3773211 0.1809857 265.8588 0.06977603    193
## 35 0.5945204 -0.5244809 0.3790187 0.1664239 265.9387 0.06931844    239
## 36 0.9617321 -0.5140342 0.3765530 0.1722202 261.5723 0.10718808     23
## 37 0.6530627 -0.5073099 0.3772709 0.1787275 265.6408 0.07137192    184
## 38 0.5305712 -0.5870343 0.3841061 0.1264349 265.1928 0.07541288    335
## 39 0.6010555 -0.5170680 0.3788249 0.1722763 266.0256 0.06866520    230
## 40 0.7741925 -0.5045558 0.3780543 0.1820034 262.3434 0.10083764    101
```

```
## 41 0.5071165 -0.6021226 0.3889672 0.1216215 265.1899 0.07583062    395
## 42 0.8645447 -0.4905761 0.3786257 0.1950872 260.7817 0.11946748     60
## 43 0.7900522 -0.5024543 0.3782778 0.1840896 261.7742 0.10661435     92
## 44 0.5090754 -0.6016134 0.3885715 0.1215574 265.1762 0.07593130    390
## 45 0.9351075 -0.5082627 0.3785522 0.1793856 259.9817 0.12379188     33
## 46 0.5372803 -0.5793030 0.3829178 0.1303139 265.3326 0.07427153    321
## 47 0.5189053 -0.5961919 0.3862847 0.1227337 265.1507 0.07592579    363
## 48 0.4960862 -0.5995351 0.3910804 0.1252702 265.4165 0.07419140    436
## 49 0.5047404 -0.6023416 0.3895581 0.1220521 265.2314 0.07554192    404
## 50 0.5207493 -0.5942811 0.3859001 0.1235636 265.1622 0.07580360    358
## 51 0.6648264 -0.5129385 0.3772990 0.1739878 265.3638 0.07348995    175
## 52 0.7359842 -0.5055166 0.3769070 0.1798478 263.3967 0.08994347    124
## 53 0.7023129 -0.5067384 0.3770119 0.1789188 264.4585 0.08070598    147
## 54 0.8209234 -0.4967894 0.3777177 0.1884293 261.5470 0.11053637     78
## 55 0.7403817 -0.5063253 0.3771678 0.1794532 263.0943 0.09250481    120
## 56 0.9692640 -0.5389920 0.3773198 0.1531552 260.4670 0.11271084     19
## 57 0.7503649 -0.5084149 0.3776022 0.1781639 262.7469 0.09600741    115
## 58 0.6085098 -0.5097583 0.3783982 0.1779326 266.0503 0.06843670    221
## 59 0.8408018 -0.4964521 0.3784020 0.1895302 261.2004 0.11543923     69
## 60 0.4945734 -0.5984257 0.3911345 0.1260231 265.4707 0.07376411    441
## 61 0.5007001 -0.6036538 0.3905129 0.1221535 265.2686 0.07528644    418
## 62 0.5625083 -0.5566999 0.3802808 0.1432164 265.6558 0.07155830    280
## 63 0.7649382 -0.5051196 0.3783549 0.1818627 262.3186 0.10056744    106
## 64 0.5974622 -0.5180208 0.3787911 0.1714488 265.9704 0.06905898    234
## 65 0.6187894 -0.5066641 0.3781863 0.1803361 266.0064 0.06873446    211
## 66 0.5833339 -0.5349607 0.3793051 0.1584304 265.8941 0.06971427    253
## 67 1.0000000 -0.5775646 0.3798403 0.1283738 259.9346 0.11802281     15
## 68 0.6716625 -0.5120791 0.3772255 0.1746262 265.1978 0.07471850    170
## 69 0.6775102 -0.5101280 0.3771553 0.1761938 265.0655 0.07580136    166
## 70 0.4935545 -0.5978536 0.3912211 0.1264695 265.4811 0.07367561    445
## 71 0.5265872 -0.5890873 0.3846715 0.1256697 265.1924 0.07546894    344
## 72 0.5348963 -0.5817300 0.3833347 0.1291276 265.2856 0.07464232    326
## 73 0.5730036 -0.5459614 0.3796935 0.1504624 265.7953 0.07048477    266
## 74 0.5118322 -0.5997654 0.3877856 0.1219496 265.1329 0.07620111    381
## 75 0.5444649 -0.5735617 0.3819687 0.1332021 265.3917 0.07369804    308
## 76 0.5286425 -0.5874836 0.3843751 0.1264102 265.2162 0.07527445    340
## 77 0.5065967 -0.6040701 0.3893425 0.1207787 265.1715 0.07598270    399
## 78 0.5805092 -0.5384435 0.3795224 0.1559750 265.8447 0.07009334    257
## 79 0.5769910 -0.5450311 0.3798401 0.1513167 265.7670 0.07066774    262
## 80 0.6344057 -0.5047784 0.3775073 0.1811784 265.9406 0.06918550    198
## 81 0.7158739 -0.5098283 0.3770513 0.1763287 264.0129 0.08460015    138
## 82 0.8729049 -0.4850844 0.3776534 0.1989776 261.4398 0.11367554     56
## 83 0.7278175 -0.5081005 0.3770907 0.1778442 263.5684 0.08831295    129
## 84 0.5591198 -0.5610635 0.3806387 0.1404801 265.5708 0.07219313    285
## 85 0.7578611 -0.5044826 0.3777201 0.1816804 262.6839 0.09671241    111
## 86 0.8093389 -0.5024353 0.3784001 0.1842480 261.3630 0.11119135     83
## 87 0.5322193 -0.5842115 0.3837015 0.1278670 265.2377 0.07504680    331
## 88 0.7091154 -0.5065483 0.3771542 0.1792460 264.2272 0.08274396    143
## 89 0.6851592 -0.5091062 0.3771666 0.1770744 264.9732 0.07652120    161
## 90 0.6132570 -0.5086968 0.3782506 0.1786681 266.0585 0.06836790    216
## 91 0.5242608 -0.5910601 0.3851369 0.1248636 265.1783 0.07560234    349
## 92 0.7222670 -0.5107136 0.3773932 0.1759705 263.6051 0.08782567    133
## 93 0.4901815 -0.5973059 0.3914811 0.1270698 265.5265 0.07332377    459
## 94 0.5426159 -0.5752218 0.3822653 0.1323829 265.3711 0.07390462    312
```

```
## 95 0.7984502 -0.5043019 0.3781231 0.1823029 261.6175 0.10869535     88
## 96 0.6972257 -0.5066324 0.3771920 0.1792175 264.6594 0.07911367    152
## 97 0.6909754 -0.5071891 0.3771423 0.1786828 264.8299 0.07773397    156
## 98 0.6590249 -0.5102355 0.3771584 0.1761063 265.5427 0.07214115    179
##      deltaAIC
## 1  6.06384092
## 2  1.07283952
## 3  5.21930167
## 4  6.09138983
## 5  5.75071254
## 6  5.61464750
## 7  5.59412345
## 8  2.14546616
## 9  5.27396801
## 10 5.42178161
## 11 5.56024342
## 12 5.56201590
## 13 5.17340468
## 14 5.31137910
## 15 5.22129985
## 16 5.81488442
## 17 5.56463165
## 18 5.40622413
## 19 0.56547967
## 20 1.18174108
## 21 1.68117485
## 22 5.81838216
## 23 5.22804281
## 24 6.07153193
## 25 5.42751139
## 26 0.21025215
## 27 1.45113532
## 28 5.87880562
## 29 5.34240589
## 30 5.61870141
## 31 5.92084808
## 32 1.38298266
## 33 5.24277108
## 34 5.92416041
## 35 6.00410053
## 36 1.63773524
## 37 5.70615624
## 38 5.25821476
## 39 6.09094542
## 40 2.40874726
## 41 5.25524141
## 42 0.84709003
## 43 1.83961699
## 44 5.24159917
## 45 0.04705413
## 46 5.39794119
## 47 5.21604910
## 48 5.48184621
## 49 5.29679193
```

```
## 50 5.22762490
## 51 5.42922238
## 52 3.46207929
## 53 4.52385166
## 54 1.61236148
## 55 3.15972748
## 56 0.53233871
## 57 2.81230075
## 58 6.11568455
## 59 1.26573705
## 60 5.53606548
## 61 5.33395251
## 62 5.72118844
## 63 2.38394409
## 64 6.03582374
## 65 6.07180321
## 66 5.95947068
## 67 0.00000000
## 68 5.26319725
## 69 5.13091354
## 70 5.54651095
## 71 5.25775727
## 72 5.35097662
## 73 5.86072933
## 74 5.19831415
## 75 5.45705689
## 76 5.28159484
## 77 5.23688891
## 78 5.91007632
## 79 5.83239442
## 80 6.00594709
## 81 4.07823938
## 82 1.50514370
## 83 3.63374669
## 84 5.63618797
## 85 2.74925915
## 86 1.42838230
## 87 5.30303575
## 88 4.29259857
## 89 5.03859318
## 90 6.12383574
## 91 5.24369539
## 92 3.67046348
## 93 5.59191131
## 94 5.43651252
## 95 1.68286041
## 96 4.72474169
## 97 4.89529416
## 98 5.60806084
```

8. Figure 1:

```
## Calculate mean eastimate and se of predictions with hab_qual = "unmanaged" and "managed":
```

```
fig1$dts_cat <- as.factor(fig1$dts_cat)
fig1 <- as.data.table(fig1)
fig1_mean <- fig1[, list("mean_fit" = mean(fit), "mean_se" = mean(se.fit)), by = c(colnames(fi
g1[, 3:7]))]

## Change dts_cat level names:
levels(fig1_mean$dts_cat) <- c("close to human settlement", "far from human settlement")

ggplot(fig1_mean, aes(x = vd_0to5, y = rep_succ, fill = dts_cat, color = dts_cat)) +
geom_jitter(size = 2, na.rm = TRUE, width = 0, height = 0.01) +
geom_line(aes(x = vd_0to5, y = mean_fit, lty = dts_cat), size = 2) +
geom_ribbon(aes(ymin = mean_fit - mean_se, ymax = mean_fit + mean_se), colour = NA, alpha = 0.
1) +
scale_color_manual(breaks = c("close to human settlement", "far from human settlement"), value
s = c("red", "blue")) +
scale_fill_manual(breaks = c("close to human settlement", "far from human settlement"), values
 = c("red", "blue")) +
ylab("probability of succesful reproduction") +
xlab("understory density") +
theme_classic(15) +
theme(legend.position = c(0.8, 0.2), legend.title = element_blank(), legend.key.size = unit(2,
 'lines'))
```

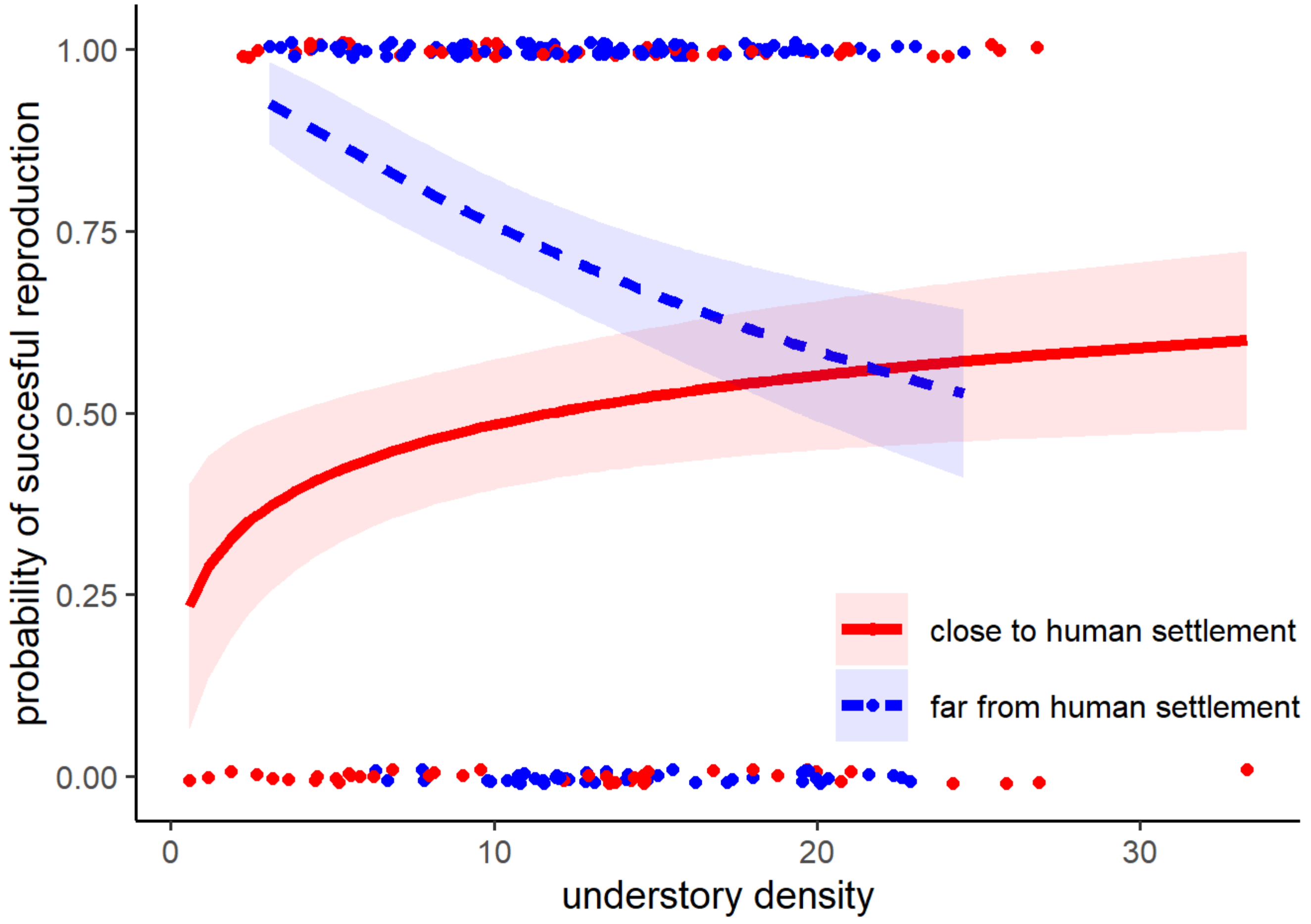

9. Figure 2:

```
## Calculate cor values used for vertical lines below:
```

```
vert_lin <- r.all_rad$cor[r.all_rad$radius %in% c(78, 202, 450)]

ggplot(r.all_rad, aes(x = cor, y = R2m)) +
geom_point(size = 4) +
scale_x_reverse() +
geom_vline(xintercept = vert_lin, color = "black", linetype = "dashed") +
geom_text(aes(x = vert_lin[2] + 0.025, label = "450 m", y = 0.115), angle = 0, size = 5) +
geom_text(aes(x = vert_lin[3] + 0.021, label = "80 m", y = 0.115), angle = 0, size = 5) +
geom_text(aes(x = vert_lin[1] + 0.025, label = "200 m", y = 0.115), angle = 0, size = 5) +
scale_color_grey(start = 0.1, end = 0.5) +
scale_fill_grey(start = 0.1, end = 0.5) +
xlab("correlation of ud at nest with ud at radius i around the nest") +
ylab("r-squared with ud at radius i") +
theme_classic(15)
```

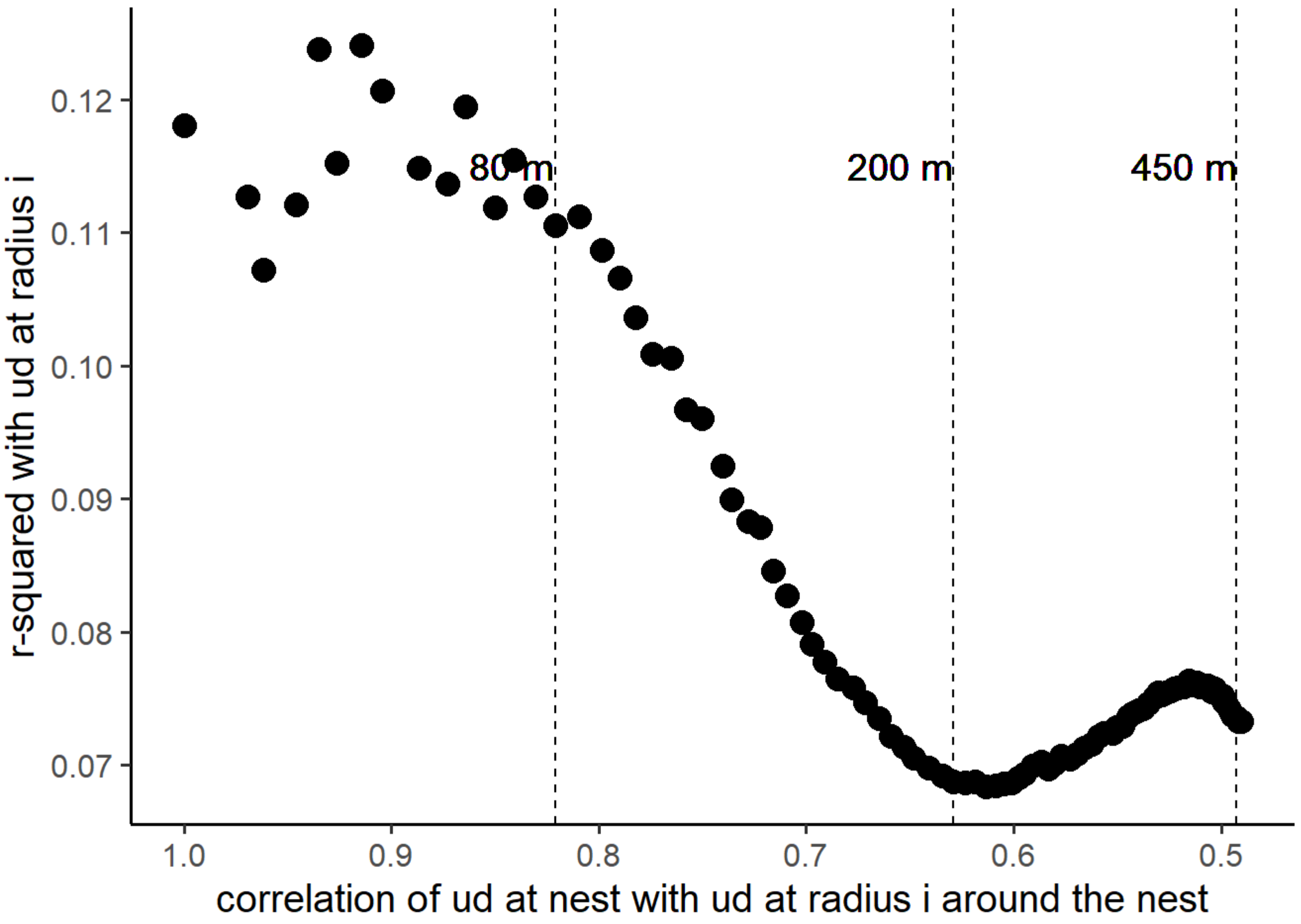

Remotely sensed forest understory density and nest predator occurrence interact to predict suitable breeding habitat and the occurrence of a resident boreal bird species - Part 4

Julian Klein

18 December 2019

Part 4: Comparison between model predictions for breeding success and siberian jay occurrence.

The LiDAR data is based on rasters extracted from .las files with FUSION. Raw Lidar data can be downloaded at http://maps.slu.se or www.lantmateriet.se. The download is not free unless you have free institutional access.

The raster showing Siberian jay occurrences will have to be required at Bradter et al. (ute.bradter@slu.se)

The raster showing distance to settlements will have to be required at Statistics Sweden (SCB) or http://maps.slu.se

The analysis presented here is based on habitat data for 15 m around the nest. The analysis for 80 m around the nest is exactly the same and correlated with 0.84

Start:

1. Load all packages:

```
library(raster)
library(Metrics)
library(ggplot2)
```

2. Define all functions:

```
## Define the formula for predicting nest success
## Results from rep_succ_analysis with 15m rad
prob_success <- function(x, y) {

  p <- 0.2411 +
    1.0427 * y + ## y = dts_low
    0.3965 * x - ## x = centered(log(vd))
    0.5196/2 - ## /2 for the mean between unmanaged/managed
    1.5703 * y * x

  return(exp(p)/(1+exp(p)))
```

```r
}

## Define dts categorisation distance
R <- 1450

## Define focal rad
rad <- 15
```

3. Load all data:

```r
lp_shape <- shapefile("data/lp_shape.shp") ## Shape of the chosen area
## p.fit.data is the prediction data with the lme4 package produced in part 2 and reimported i
nto part 3 here
p.fit.data <- read.csv("data/p.vd0t5_log_c.csv")
head(p.fit.data)
```

```
##         fit     se.fit rep_succ dts_cat   vd_0to5
## 1 0.8280367 0.06230376        1     far  6.830820
## 2 0.7258035 0.05941166        1     far 11.581750
## 3 0.7961298 0.06050731        1     far  8.217848
## 4 0.7119857 0.06039511        0     far 12.302175
## 5 0.6913071 0.06282923        1     far 13.422980
## 6 0.5500986 0.09096020        1   close 12.609701
```

```r
vd_lp <- raster("data/Dens_05_5_mosaic.tif")*100
## *100 because input between 0 and 1 but formula expects between 0 and 100
plot(vd_lp); plot(lp_shape, add = TRUE)
```

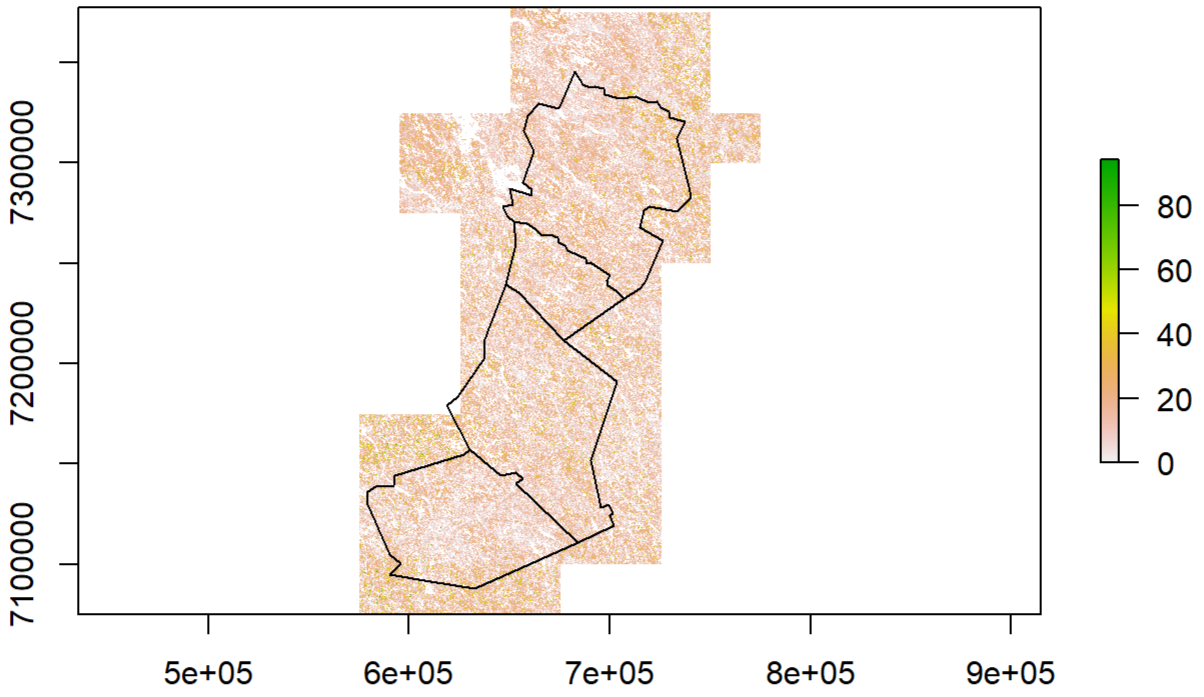


```
dts <- raster("data/dts.tif")
plot(dts); plot(lp_shape, add = TRUE)
```

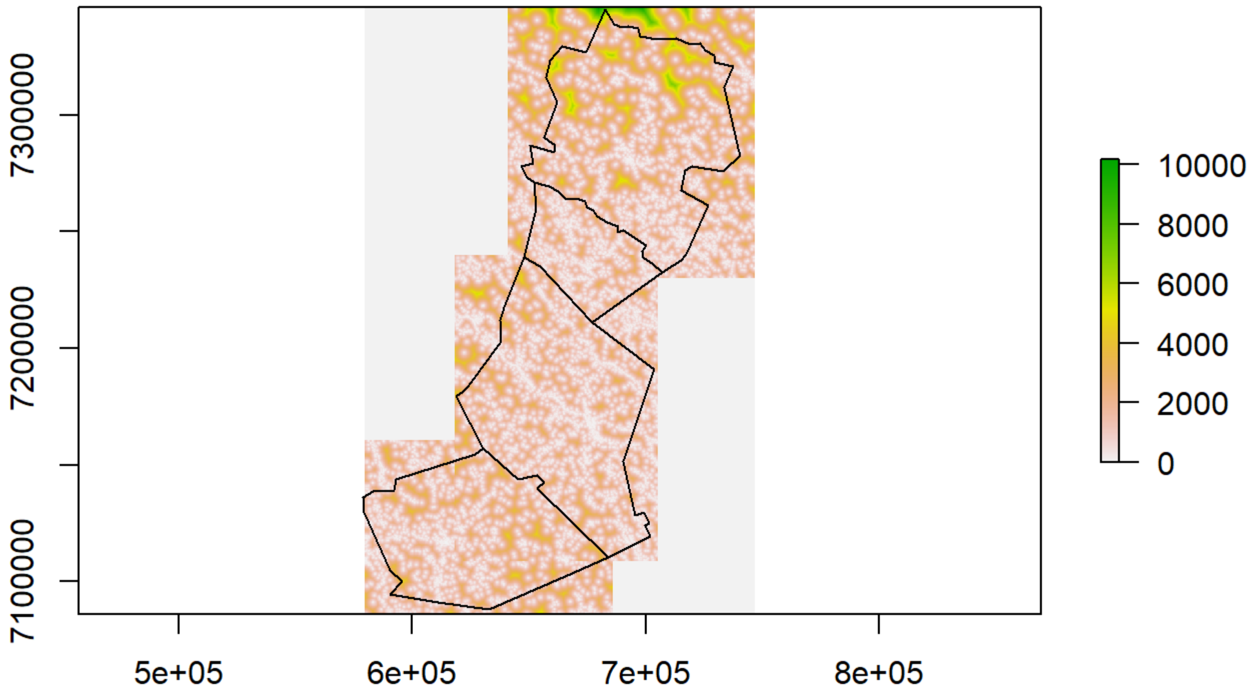


```
sj_occ <- raster("data/SFBestCellNbhMainland_99TM.tif")
plot(sj_occ); plot(lp_shape, add = TRUE)
```

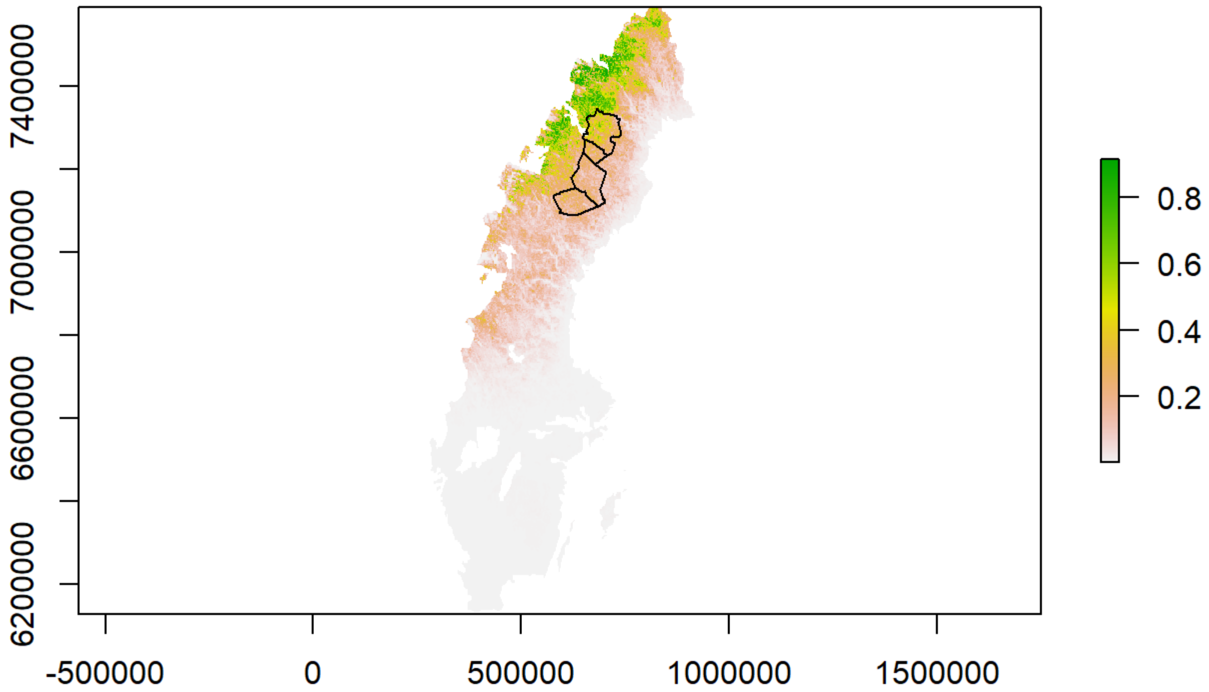

4. Adjust the raster files and predict breeding success for Arvidsjaur, Malå, Lycksele and Åsele

```
## Reduce vd_lp to the extent of dts
vd_lp <- crop(vd_lp, extent(dts))

## Average vd_lp within radius used for the prediction in the study

## Define raster with value = 1 for all cells where understorey is not NA
num_vd_lp <- vd_lp
num_vd_lp[!is.na(num_vd_lp)] <- 1

#Define focal filter
f <- focalWeight(vd_lp, d = rad, type = "circle")
f[f > 0] <- 1

#Calculate sum of raster cells in focal filter
vd_lp_sum <- focal(vd_lp, f, fun = sum, na.rm = TRUE)

#Calculate number of raster cells in focal filter
vd_lp_num <- focal(num_vd_lp, f, fun = sum, na.rm = TRUE)

## Calculate mean within rad
vd_lp_mean <- vd_lp_sum / vd_lp_num

## Calculate centered log of vd_lp_mean
C <-  mean(log(p.fit.data$vd_0to5), na.rm = TRUE) ## Define C:
```

```
vd_lp_log_cent <- log(vd_lp_mean)-C

## Change the resolution of dts
dts <- disaggregate(dts, fact = 4)

## Categorise dts
dts_cat <- dts >= R
```

```
## Predict p(successful reprduction)
## Overlay fastest. Tested against calc and manual calculation. Results of all three the same
t1 <- Sys.time()
lp_out <- overlay(vd_lp_log_cent, dts_cat, fun = prob_success)
t2 <- Sys.time()
t2-t1
```

```
## Time difference of 1.373872 mins
```

```
## All NA's in lp_out become 0 as they are non-habitat and no reproduction is expected there
lp_out[is.na(lp_out)] <- 0
```

```
## Reduce lp_out to range found in study
range_far <- c(min(p.fit.data$vd_0to5[p.fit.data$dts_cat == "far"]), max(p.fit.data$vd_0to5[p.
fit.data$dts_cat == "far"]))
range_close <- c(min(p.fit.data$vd_0to5[p.fit.data$dts_cat == "close"]), max(p.fit.data$vd_0to
5[p.fit.data$dts_cat == "close"]))

## Define rasters that will become NA after the prediction
NA_far <- vd_lp_mean <= range_far[1] | vd_lp_mean >= range_far[2]
NA_close <- vd_lp_mean <= range_close[1] | vd_lp_mean >= range_close[2]
```

```
## Set non study range predictions to 0. Not selected => no reproduction
lp_out[dts_cat == 1 & NA_far] <- 0
lp_out[dts_cat == 0 & NA_close] <- 0
```

5. Compare P(succ_repr) with p(Occurence):

```
## Resample sj_occ to resolution of lp_out
lp_out_resamp <- resample(lp_out, sj_occ)

## Reduce both to the Kommun borders of Arvidsjaur, Åsele. Malå and Lycksele
lp_out_resamp <- mask(lp_out_resamp, lp_shape)
sj_occ <- mask(sj_occ, lp_shape)

## Compare lp_out with sj_occ:
cor.test(sj_occ[], lp_out_resamp[])
```

```
##
##  Pearson's product-moment correlation
##
## data:  sj_occ[] and lp_out_resamp[]
```

```
## t = 29.702, df = 4529, p-value < 2.2e-16
## alternative hypothesis: true correlation is not equal to 0
## 95 percent confidence interval:
##  0.3791164 0.4278658
## sample estimates:
##       cor
## 0.4037777
```

```
## Look at disagreement after normalisation of both rasters

lp_norm <- (lp_out_resamp - min(lp_out_resamp[], na.rm = TRUE))/(max(lp_out_resamp[], na.rm =
TRUE) - min(lp_out_resamp[], na.rm = TRUE))
sjo_norm <- (sj_occ - min(sj_occ[], na.rm = TRUE))/(max(sj_occ[], na.rm = TRUE) - min(sj_occ[]
, na.rm = TRUE))

disag <- sjo_norm - lp_norm

plot(crop(disag, extent(lp_shape)), col = bpy.colors())
```

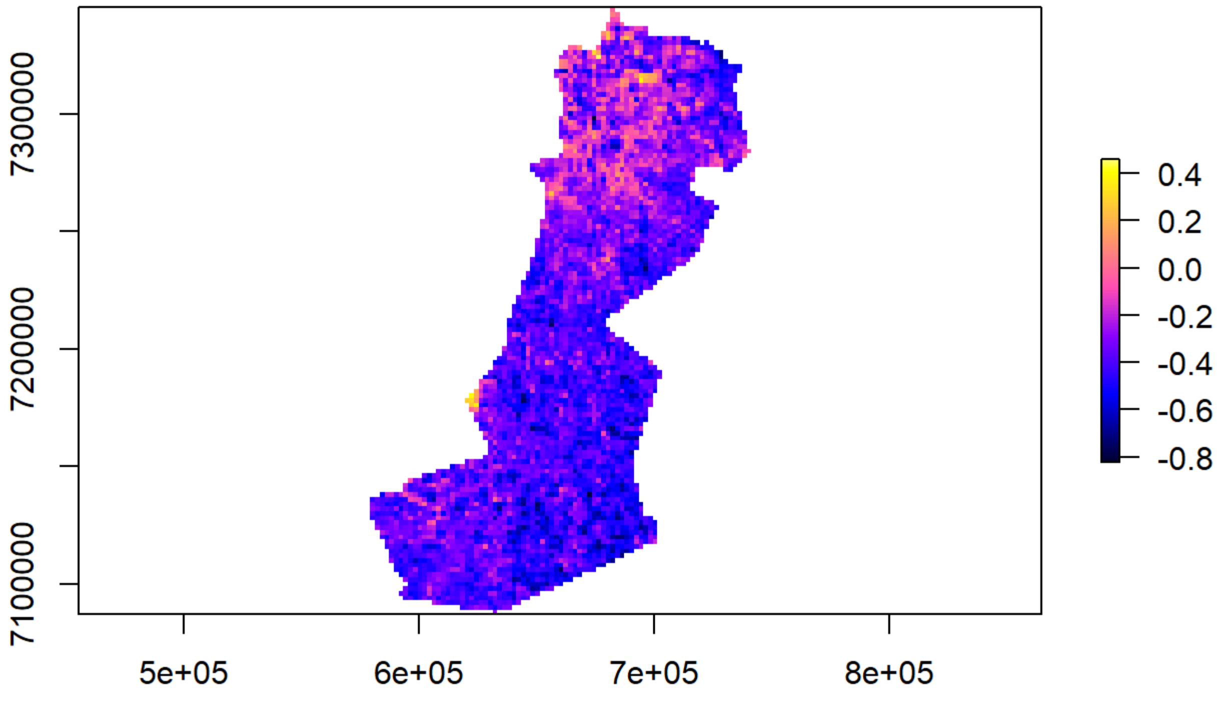


```
## Create a figure showing the density of different disagreement values

bdg <- rbind(expand.grid("disag" = na.omit(mask(disag, lp_shape[lp_shape$KnNamn == "Arvidsjaur
", ])[]),
                         "county" = "Arvidsjaur"),
             expand.grid("disag" = na.omit(mask(disag, lp_shape[lp_shape$KnNamn == "Malå", ])[
```

```
]),
                         "county" = "Malå"),
             expand.grid("disag" = na.omit(mask(disag, lp_shape[lp_shape$KnNamn == "Lycksele",
 ])[]),
                         "county" = "Lycksele"),
             expand.grid("disag" = na.omit(mask(disag, lp_shape[lp_shape$KnNamn == "Åsele", ])
[]),
                         "county" = "Åsele"))

## Compare the counties
pairwise.t.test(bdg$disag, bdg$county, p.adjust.method = "bonferroni")
```

```
##
##  Pairwise comparisons using t tests with pooled SD
##
## data:  bdg$disag and bdg$county
##
##          Arvidsjaur Malå   Lycksele
## Malå     <2e-16     -      -
## Lycksele <2e-16     <2e-16 -
## Åsele    <2e-16     <2e-16 0.038
##
## P value adjustment method: bonferroni
```

```
ggplot(bdg) +
geom_vline(xintercept = c(0.25, 0, -0.25, -0.5, -0.75), linetype = "dashed", color = "grey") +
geom_density(aes(x = disag, color = county, linetype = county), size = 1) +
scale_x_continuous(breaks = c(0.25, 0, -0.25, -0.5, -0.75)) +
xlab("disagreement values") +
labs(color = "counties from north to south", linetype = "counties from north to south") +
theme_classic(15) +
theme(legend.position = c(0.75, 0.8), legend.key.size = unit(1, 'lines'))
```

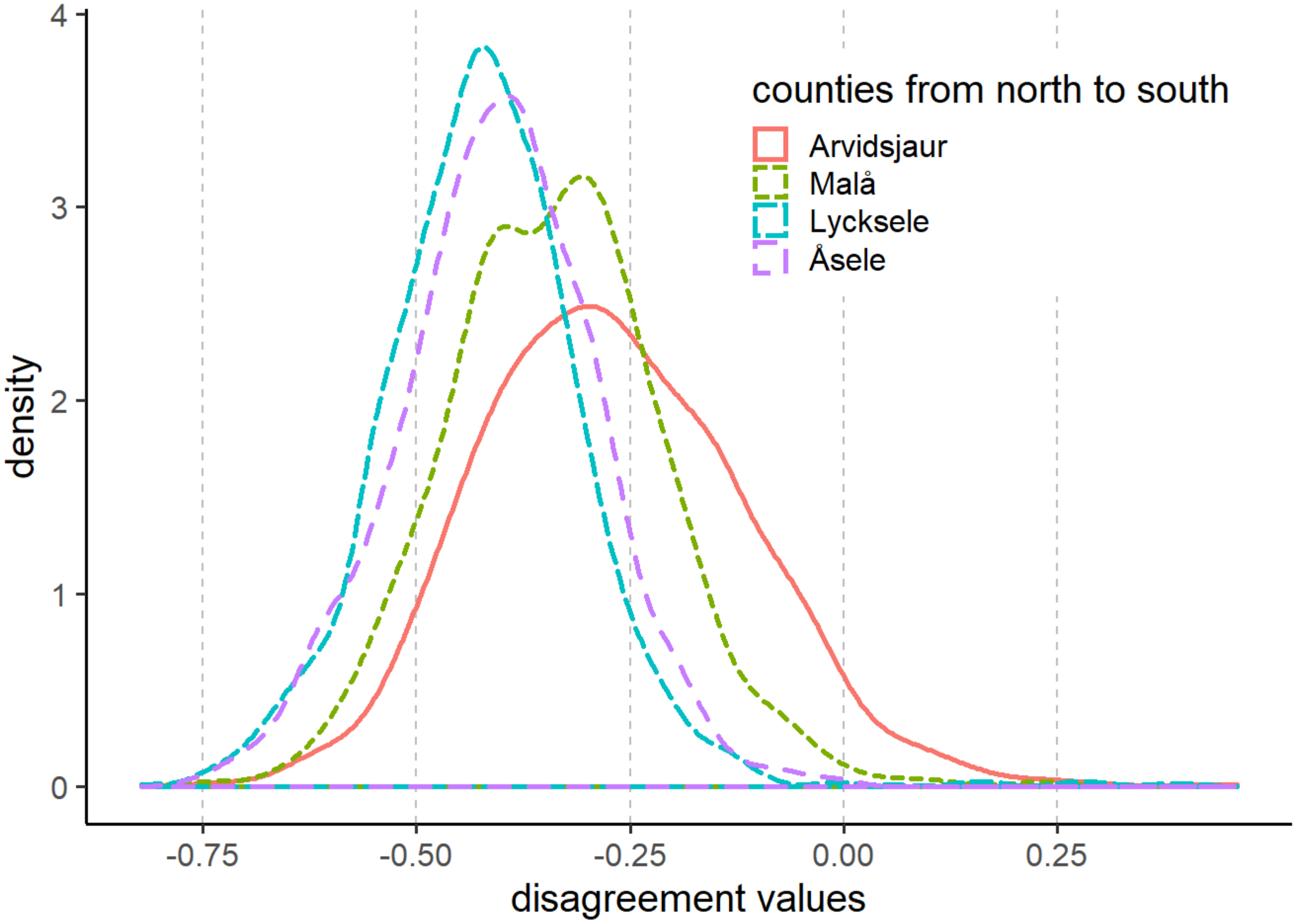
counties from north to south
Arvidsjaur
Malå
Lycksele
Åsele
density
disagreement values
4
3
2
1
0
-0.75
-0.50
-0.25
0.00
0.25
